# The bacteria of a fig community

**DOI:** 10.1128/spectrum.03013-24

**Published:** 2026-03-30

**Authors:** Gavin C. Woodruff, Kimberly A. Moser, John Wang

**Affiliations:** 1 School of Biological Sciences, University of Oklahoma 6187, Norman, Oklahoma, USA; 2 Biodiversity Research Center, Academia Sinica 38017, Taipei City, Taiwan; Brigham Young University, Provo, Utah, USA

**Keywords:** figs, fig wasps, microbial ecology, *Caenorhabditis*, *C. elegans*

## Abstract

Understanding the biotic drivers of diversity is a major goal of microbial ecology. One approach toward tackling this issue is to interrogate relatively simple communities that are easy to observe and perturb. Figs (syconia) of the genus *Ficus* represent such a system. Here, we describe the microbial communities of *Ficus septica* figs, which are associated with the nematode *Caenorhabditis inopinata* (the sister species of the *C. elegans* genetic model system). In 2019, 38 *Ficus septica* figs (across 12 plants in Taiwan) were dissected, and metadata, such as foundress wasp number and nematode occupancy, were collected for each fig. Suspensions derived from interior fig material and fig surface washes were prepared for 16S microbial metabarcoding. Over 2,000 ASVs were detected, and microbial communities were dominated by members of *Proteobacteria*, *Bacteroidota*, and *Actinobacteriota*. Although microbial communities of fig exteriors and interiors can be distinguished, levels of microbial alpha diversity were comparable across these areas of the fig. A joint analysis of fig and previously published *C. elegans* substrate microbiomes revealed similarities and differences among the biotic environments of these nematode sister species. Fig microbial community composition was driven in large part by variation among individual plants. Conversely, nematodes had no detectable impact on microbial community composition or alpha diversity. A handful of ASVs (associated with the genera *Ochrobactrum* and *Stenotrophomonas*) revealed potential differential abundance among figs varying in nematode occupancy. Additionally, foundress wasp number was negatively correlated with microbial alpha diversity. These findings set the stage for future studies that directly test the role of nematode and wasp occupancy on microbial communities, as well as investigations that probe nematode-microbe interactions through laboratory experiments. Taken together, these results constitute a fundamental step in characterizing the natural microbial communities of figs and *Caenorhabditis* nematodes.

**IMPORTANCE:**

Unraveling why different species live in different places is a longstanding open question in ecology. It is clear that interspecific interactions among species are a major contributor to species distributions. *Ficus* figs are a useful system for ecological studies because they are relatively simple microcosms where characterizing animal community composition of multiple samples is straightforward. Additionally, *Caenorhabditis inopinata*, a close relative of the *C. elegans* genetic model system, thrives in *Ficus septica* figs. Here, we tie 16S microbial metabarcoding to nematode and wasp occupancy data to understand the causes of bacterial community composition in *F. septica* figs. We found that microbial composition, but not total diversity, varies among fig surface and interiors. Additionally, microbial diversity is driven in large part by individual plant of origin. Likewise, we found that nematode occupancy does not appear to impact microbial composition. Moreover, we show that as the number of foundress wasps increases, the microbial α-diversity decreases. Finally, we identified ASVs that are potentially associated with nematode occupancy. Taken together, these results represent a key step in describing a community wherein ecological genetic hypotheses can be tested, as well as one that can potentially reveal the roles of uncharacterized genes in established model systems.

## INTRODUCTION

Ecology seeks to explain species distributions. Biotic interactions are critical drivers of such distributions, as predator/prey, host/parasite, and interspecific competitive relationships (among others) contribute to the ability of an organism to thrive in a given place. However, while demonstrating that a given interaction causes species occupancy is notoriously difficult (e.g., demonstrating a host–parasite interaction has a large effect on a species distribution), even demonstrating the existence of an interspecific interaction in itself can be a challenge (1, 2). Furthermore, simply noting the presence of an interaction is insufficient—the frequency and effect size of such interactions must be estimated to understand if they are relevant to species occupancy patterns. Moreover, manipulative experiments are often required to demonstrate causation, and manipulatable ecological systems are usually expensive (3). Thus, one avenue toward tackling ecological questions is through the use of systems where species interactions are easy to assess, quantify, and manipulate.

Figs (also known as syconia) of the genus *Ficus* represent such a system. Although known in the common parlance as fruit (and mature figs are fruits indeed) (4), figs are more appropriately described as an inflorescence with the flowers facing inward (5). Figs are pollinated by fig wasps, and fig wasps, in turn, lay their eggs in fig ovules (4). The flowers are accessible to pollinators only via a specialized opening called the ostiole (6). The ostiole is protected by a series of modified leaves (i.e., bracts) that presumably hinder animal penetration (6, 7). These bracts can apparently cause the loss of antennae, wings, and appendages of pollinating wasp foundresses (4, 8). While an extensive literature regarding the fig/fig wasp mutualism exists (9–13), more species interact with figs than pollinating wasps alone (although multiple species of fig wasp have been reported to pollinate a single fig) (14). Non-pollinating fig wasps are common, with more than one species of wasp frequently parasitizing a single fig species (9). Moreover, non-pollinating wasps can fill a variety of ecological niches within the fig community, living as gallers, wasp parasitoids, kleptoparasites, and seed eaters (9). Various species of fungi have been observed in figs (15). Ants are also associated with figs and can exert protective effects (16). Likewise, moth larvae can prey on fig tissue, potentially impacting community members (17). Additionally, mites (18), nematodes (12), single-celled eukaryotes, and bacteria also contribute to the fig habitat (19, 20).

Additionally, this complex community is also amenable to ecological work. Figs are small, discrete, individualized communities, where species occupancies and abundances can be easily ascertained. Additionally, multiple figs from a single individual plant can be easily sampled and observed, affording reproducibility and large sample sizes (although variation among individual plants is ample). Fitness proxies for multiple community members can be assessed (e.g., seed count, wasp progeny number) (4). While the community is complex, the species composition of figs is not so extreme as to make the number of possible interactions intractable—that is, it strikes a balance between system complexity and observational feasibility. Furthermore, this system can be experimentally manipulated in various ways. For instance, nets can be placed over figs to prevent wasp entry (21). Alternatively, antibiotic, antifungal, or anthelmintic compounds could plausibly be added to figs to manipulate bacterial, fungal, or nematode occupancy. Additionally, as wasp (9) and nematode (22, 23) demographics and occupancy can change across fig development, theories of ecological succession (24) can also be applied to and tested with the fig system. For these reasons, figs are well suited for ecological studies.

Nematodes are a particularly attractive subject of fig biology because of the widespread ecological diversity of fig nematodes (12). Nematode diversification has been extensive even within a single fig species (eight species of nematodes were observed within *F. burkei* (25)). Nematode fig parasites (e.g., *Schistonchus*, *Martinema*, and *Ficophagus*) (26), wasp parasites (*Parasitodiplogaster* and *Teratodiplogaster*) (27, 28), fungal feeders (*Bursaphelenchus* [29]), bacterivores (*Caenorhabditis*, *Pristionchus*, and *Acrostichus* [22, 25, 30]), and nematode predators (*Pristionchus*) all thrive in the fig microcosm. Additionally, all are thought to disperse on fig wasps to enable propagation in fresh figs. Consistent with this extensive ecological diversity, nematodes have evolved to live in figs at least eight times independently (12). The nematode *Caenorhabditis inopinata*, associated with *F. septica*, is of particular interest because it is the closest known relative of the widely used genetic model system, *C. elegans* (30).

Despite its 50-year status as a genetic model (31), hundreds of *C. elegans* genes remain uncharacterized (32). Presumably, one reason so many genes remain cryptic in their function is because this system is frequently studied in the laboratory, removed from its natural context (32), which is typically rotting plant detritus in the wild (33, 34). In the past decade, the natural microbial environment of *C. elegans* has been characterized (35–38), and this information could be used to enable laboratory experiments aimed at discovering novel gene functions (39).

16S amplicon sequencing can be used to describe the extent of bacterial diversity in a sample (40). As *Caenorhabditis* nematodes are bacterivores that can likewise experience myriad beneficial and detrimental effects via microbial interactions (41), we seek to understand the extent of microbial diversity in the natural environment of *C. inopinata* (whose natural food source is undescribed). Moreover, as bacterivores may be expected to impact microbial community members by consuming them as food, the microbes that nematodes eat should vary in abundance depending on nematode presence. Beyond this, microbes have profound impacts across community trophic levels in general (42), and microbial diversity is expected to impact nematode, wasp (43), and fig (44) physiology and fitness. Nothing is known about the microbial communities of *Ficus septica* figs. Here, we aim to describe the natural microbial environment of *C. inopinata* to (i) harness the fig microcosm to understand the ecological drivers of organismal occupancy and diversity and (ii) provide a comparative context to enable laboratory and field studies aimed at understanding how host/microbe interactions evolve. To this end, we performed 16S microbial metabarcoding on fresh *F. septica* figs with variable nematode occupancy and pollinating foundress wasp number to understand whether and how they drive patterns of microbial diversity.

## RESULTS

### 16S metabarcoding reveals bacteria associated with *F. septica* figs

**Fig 1:**
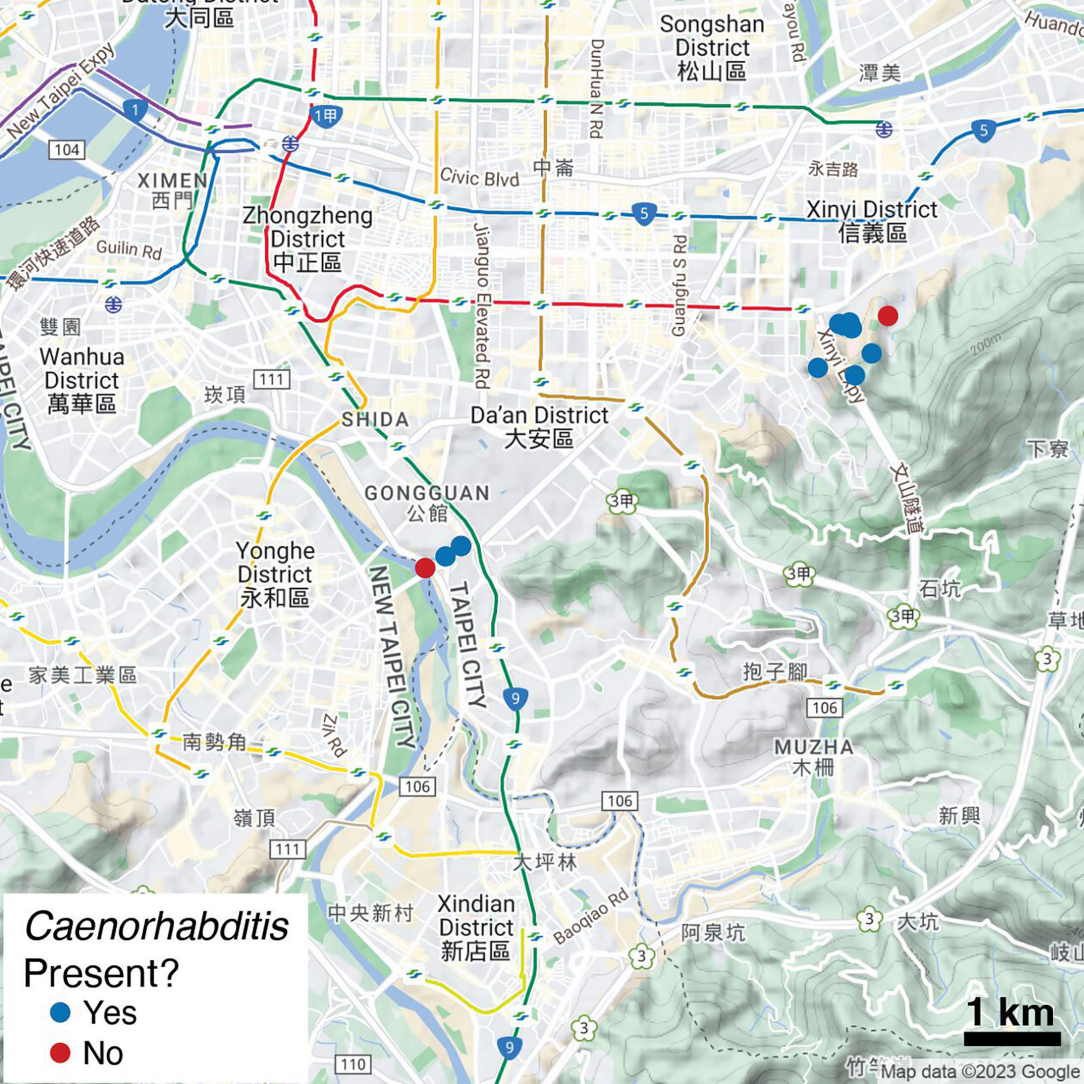
Sampling localities. *F. septica* figs were sampled in southern Taipei, Taiwan. Points represent the locations of individual *F. septica* plants sampled in this study. Points are colored by whether or not any figs observed from a given plant harbored *Caenorhabditis* nematodes. The scale bar notes 1 km. Maps were generated in part with Google Maps and their data sources. 2023 Google (https://about.google/brand-resource-center/products-and-services/geo-guidelines/).

To describe the microbial taxa associated with *C. inopinata*, we performed 16S metabarcoding (see Materials and Methods section) on substrates from this species’ natural environment. Thirty-eight figs from 12 *F*. *septica* plants in Taipei, Taiwan, were sampled in August 2019 (Fig. 1). Two different DNA preparations were generated for each fig: (i) before dissection, the fig was washed in buffer, and this surface wash was then used for downstream sequencing (known herein as “fig surface wash” samples); (ii) the fig was then cut into four pieces in buffer, and a fraction of the resultant fig suspension was used for downstream sequencing (known herein as “fig suspension” samples). Such differences in sample preparation aimed to identify different biologically relevant features of fig microbial communities. Specifically, pollinating wasps and fig nematodes do not thrive on the fig surface, but rather grow and reproduce in the fig lumen (12, 45). We used these two different methodological approaches to identify differences in the surface (surface washes) and interior (fig suspensions) fig microbiomes. Although fig suspensions will necessarily include fig surface microbes, this approach would presumably facilitate the identification of microbes specific to or enriched in the fig interior.

In addition to microbial metabarcoding, additional observations were collected for each dissected fig, including nematode occupancy and foundress pollinating wasp number (see Table S1, Sheet S2 for all fig-specific ecological data). This enabled the testing of hypotheses regarding the impacts of nematodes and wasps on fig microbial diversity. 16S libraries from these samples were prepped and sequenced, resulting in 11,361,994 250-bp paired-end reads (140,272 paired-end reads on average per sample; Fig. S1; Table S1, Sheet S3). Reads were then clustered to infer amplicon sequence variants (ASVs); 9,459,003 paired-end reads were ultimately used to infer microbial communities (see Materials and Methods section; 116,778 paired-end reads on average per sample; Fig. S1). An initial analysis revealed that the reads were dominated by organellar DNA, presumably originating from host tissue (36% of the reads were mitochondrial or chloroplast in origin on average per sample; per-sample range 0–94%; Fig. S2). After excluding these reads, 5,515,057 paired-end reads remained for downstream analyses (68,087 paired-end reads on average per sample; Fig. S1).

**Fig 2:**
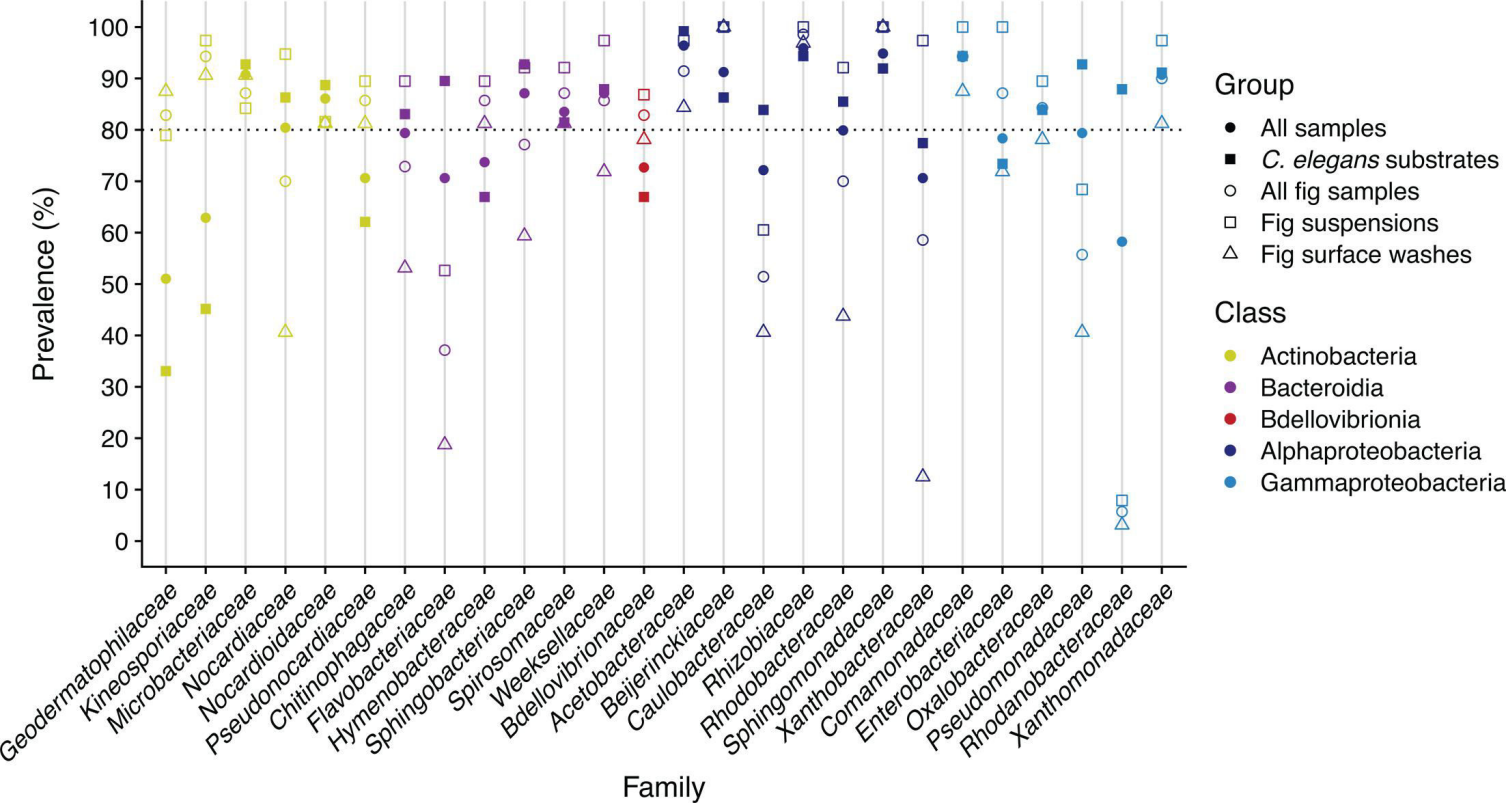
Bacterial family prevalence in *C. inopinata* and *C. elegans* substrates. Families prevalent in 80% of the samples (with respect to a given subset of the samples: all samples, *C. elegans* substrate samples, all fig samples, fig surface washes, or fig suspensions) were plotted. Note that in the Silva taxonomy, the family *Spirosomataceae* is listed as *Spirosomaceae*. In addition, in this taxonomy, *Comamonadaceae* is classified as a member of the class Gammaproteobacteria despite its status as a member of Betaproteobacteria in other sources (51).

Highly prevalent taxa are frequently described as constituting a “core” microbiome for a given host (46). For an initial description of *Ficus septica* microbial communities, we examined the most prevalent taxa in our samples. We detected 2,845 ASVs among 321 genera in our fig samples. Here, no ASVs nor genera were observed in 100% of our fig samples (Fig. S3). Three genera were observed in 97% (68/70) of the fig samples: *Methylobacterium/Methylorubrum* (47); *Allorhizobium/Neorhizobium/Pararhizobium/Rhizobium* (hereafter shortened to *Allorhizobium* (48)); and *Sphingomonas* (49). As these ASV- and genus-level taxonomic ranks typically revealed lower prevalence (median ASV prevalence = 1.4%; median genus prevalence = 4.3%; *N* = 70 fig samples), we proceeded to describe fig microbial diversity at the family level (Fig. 2). Twenty bacterial families were observed in at least 80% of fig samples (which we refer to here as “high prevalence”) when considering either all samples together or within-group prevalence in fig suspensions or fig surface washes alone (Fig. 2). These families are nested within the taxonomic classes Actinobacteria, Bacteroidia, Alphaproteobacteria, and Gammaproteobacteria (Fig. 2). Twelve families revealed high prevalence in all fig samples, including *Acetobacteraceae*, *Beijerinckiaceae*, *Rhizobiaceae*, *Spirosomaceae*, and *Kineosporiaceae* (Fig. 2). Of the eight remaining families, six had high prevalence in fig suspensions but <80% prevalence in fig surface washes (*Nocardiaceae*, *Chitinophagaceae*, *Sphingobacteriaceae*, *Rhodobacteraceae*, *Oxalobacteriaceae*, and *Xanthobacteraceae*; Fig. 2). In contrast, two families had high prevalence in fig surface wash samples but only 71% (*Geodermatophilaceae*) and 68% (*Microbacteriaceae*) prevalence in fig suspension samples (Fig. 2). Ultimately, 2,845 ASVs, 321 genera, and 185 families were identified in our fig samples. Broadly, fig ASVs were largely associated with the Proteobacteria (35.5% of ASVs), Bacteroidota (18.3%), and Actinobacteriota (9.5%) phyla. This suggests that *F. septica* figs harbor a Proteobacteria-rich microbiome, typical of those associated with plants (50).

### *C. elegans* and *C. inopinata* substrates are associated with different bacteria

*F. septica* figs are the natural habitat of *C. inopinata* nematodes (23, 30). As *C. inopinata* is the sister species of the *C. elegans* model system (30), the definition of the microbiome of *F. septica* figs affords a comparison with microbial communities associated with the natural environment of *C. elegans*. To enable such a comparison, we retrieved bacterial 16S amplicon sequencing data from two studies (36, 37) that examined substrates associated with *C. elegans* nematodes (Table S1, Sheets S4 and S5; Dirksen et al. substrates, *N* = 64; Samuel et al. substrates, *N* = 60). These substrates include compost (*N* = 62, all from Dirksen *et al*. [36]), rotting fruits (*N* = 52, mostly apples from Samuel et al. [37]), rotting stems (*N* = 6, all from Samuel et al. [37]), and invertebrate vectors (*N* = 4, all from Samuel et al., 2016 [37]). These reads, together with those from our fig samples, were then analyzed with the same workflow to compare the bacteria associated with *C. elegans* and *C. inopinata* substrates. This necessitated using only the forward reads of the paired-end sequence data from our fig samples and that from (36), as the (37) data set consisted only of forward reads (see Materials and Methods section). Thus, there are slight differences among the inferences of fig microbiomes in this joint analysis compared with analyses including all paired-end reads. In this section, only the analysis including both fig samples and *C. elegans* substrates (with forward reads only) will be discussed.

Like our *C. inopinata* substrates, no single ASV was observed in all *C. elegans* substrates. The three most prevalent ASVs across the *C. elegans* substrates included an *Allorhizobium* ASV (76%), a member of *Xanthomonadaceae* (75%), and a *Stenotrophomonas* ASV (73%). Two genera were observed in >90% of *C. elegans* substrate samples (*Pseudomonas*, 93%; *Allorhizobium*, 91%). Nineteen families were highly prevalent (>80%) in the *C. elegans* samples, and 14 of these overlapped with highly prevalent families among some subset of our fig samples (Fig. 2). Four families exhibited high prevalence in the *C. elegans* substrates but not our fig samples (Fig. 2; *Rhodanobacteraceae*, *Flavobacteriaceae*, *Caulobacteraceae*, *Pseudomonadaceae*); seven families exhibited high prevalence among some subset of the fig samples but not in the *C. elegans* substrates (Fig. 2; *Geodermatophilaceae*, *Pseudonocardiaceae*, *Kineosporiaceae*, *Hymenobacteraceae*, *Enterobacteriaceae*, *Bdellovibrionaceae*, and *Xanthobacteraceae*). Thus, there was a considerable degree of overlap among the highly prevalent families in the bacterial communities of substrates associated with *C. elegans* and *C. inopinata*.

Despite this overlap at the family level, far more ASVs were detected in the *C. elegans* substrates (58,457) compared to the *C. inopinata* fig substrates alone (3,662). The vast majority of these were associated with the compost samples interrogated in the Dirksen et al. (36) study (49,467 ASVs). Consistent with this, within-sample diversity was far higher in *C. elegans* substrates compared to *C. inopinata* substrates using three measures of α-diversity (Fig. S4; number of ASV, Shannon diversity, and phylogenetic diversity; Cohen’s *d* effect size range = 1.21–1.27; Wilcoxon rank-sum test *P* < 0.001 for all diversity measures). This result was not impacted after accounting for the non-independence of figs sampled from the same plant (Fig. S25). However, this was largely driven by high diversity in the compost samples in the Dirksen et al. study (Fig. S5; Cohen’s *d* effect size range = 3.02–3.49 when comparing the Dirksen et al. samples with fig samples; Dunn test FDR-adjusted *P* < 0.001 for all diversity measures). The rotting apples from the Samuel *et al.* study reveal comparable levels of within-sample diversity compared to our fig samples (Fig. S5; Cohen’s *d* effect size range = −0.11–0.80 when comparing the Samuel et al. samples to fig samples; Dunn test FDR-adjusted *P* = 0.053–0.79). Rarefaction had little impact on these inferences (Fig. S6). Indeed, no clear relationship between read count and α-diversity was observed (Fig. S7), so all analyses included all reads. Taken together, it is clear that *C. elegans* is capable of thriving in environments associated with variable levels of microbial diversity (including highly diverse compost substrates), whereas *C. inopinata* is generally restricted to an environment that is less bacterially diverse.

**Fig 3:**
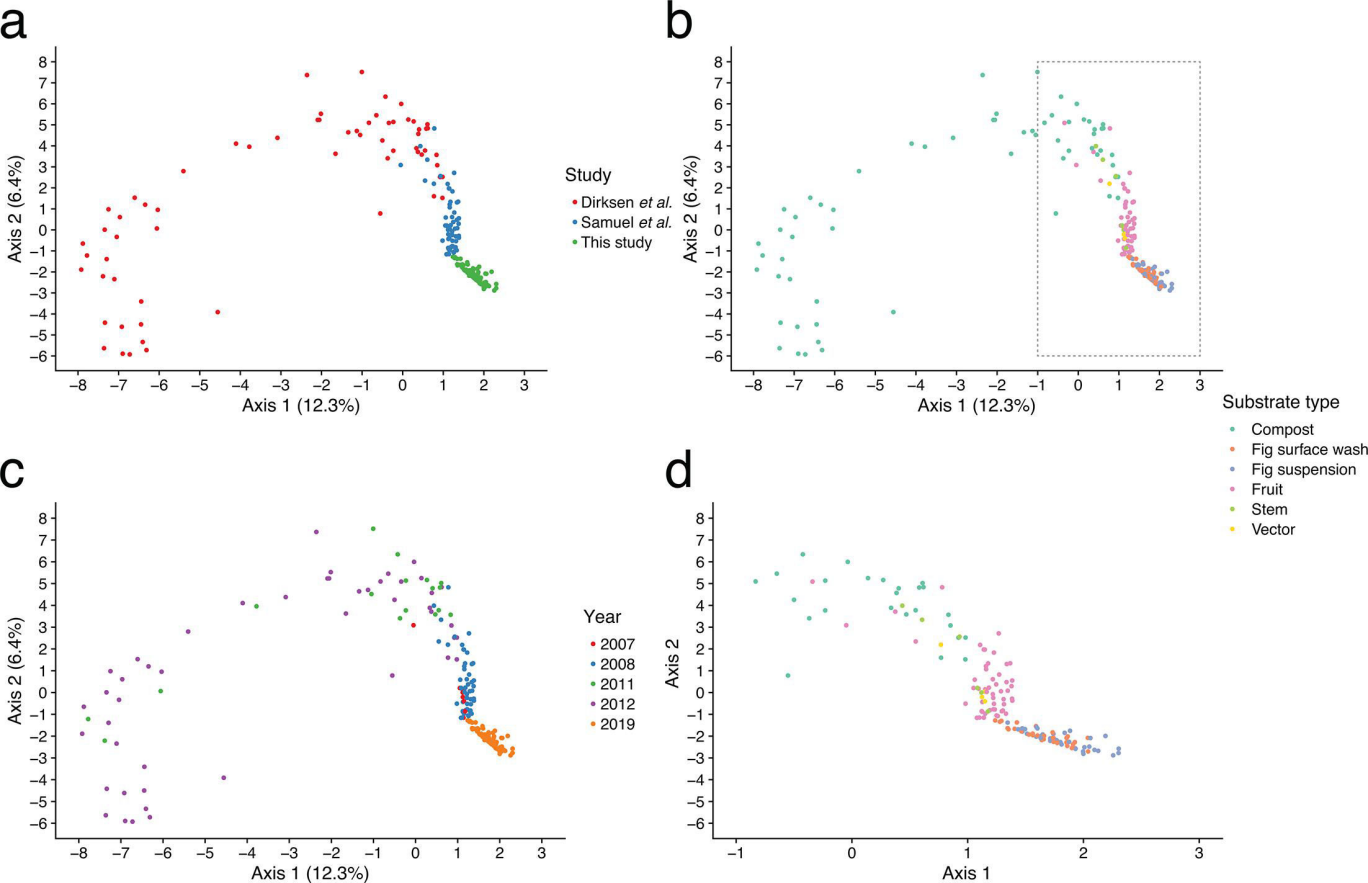
The microbial communities of *C. elegans* and *C. inopinata* substrates can be distinguished. Principal coordinate analysis using Aitchison distances reveals distance gradients among sample types. Each point represents a sample. All panels show the same ordination (with different features emphasized in each panel) (36) and (37) studies examined substrates associated with *C. elegans*. This study examined fig substrates associated with *C. inopinata*. The first axis separates samples by nematode species, study, and sample type. (**a**) Samples colored by study of origin. (**b**) Samples colored by substrate type. Here, “vector” describes four invertebrate carrier substrates (two slugs and two snails) described in Samuel et al. (37). The dotted box is expanded in the next figure panel. (**c**) Samples colored by year of sample isolation. (**d**) Expands the boxed area of panel **b** to visualize separation among non-compost substrate types.

Consistent with these differences in α-diversity among *C. elegans* and *C. inopinata* substrates, the bacterial composition among substrates associated with the two nematode species is distinct (Fig. 3; Table S1, Sheet S6; PERMANOVA *P* = 0.001; *F* = 11.6; pseudo-*r^2^* = 0.057). As with within-sample diversity (Fig. S5), much of the variation between samples was driven by the Dirksen et al. compost samples (Fig. 3; Table S1, Sheet S6). To discover the specific bacterial taxa driving differences among substrates associated with specific nematode species, differential abundance analyses were performed (see Materials and Methods section). Ninety-seven ASVs, 210 genera, and 133 families were found to be differentially abundant among *C. elegans* and *C. inopinata* substrates (Table S1, Sheet S7). More ASVs were overrepresented in *C. inopinata* (63) than in *C. elegans* substrates (34). However, at higher taxonomic ranks, more taxa revealed higher abundance in *C. elegans* substrates (142 genera and 103 families) than those overrepresented in *C. inopinata* substrates (68 genera and 30 families). The most differentially abundant families in *C. elegans* substrates include *Saprospiraceae*, *Pirellulaceae*, and *Rhodanobacteraceae*; those more abundant in *C. inopinata* substrates include *Deinococcaceae*, *Beijerinckiaceae*, and *Enterobacteriaceae* (Fig. S8).

Moreover, ASVs inferred here can be connected to databases to reveal potential functional differences among microbial communities associated with nematode species. FAPROTAX (52) revealed 33 functional categories with differential ASV representation among *C. elegans* and *C. inopinata* substrates (Fig. S9; Table S1, Sheet S7). Functional groups enriched in *C. elegans* samples included “nitrification,” “cellulolysis,” and “xylanolysis,” consistent with the compost samples associated with *C. elegans* populations. Conversely, the groups “photosynthetic_cyanobacteria” and “oxygenic_photoautotrophy” were overrepresented in the *C. inopinata* substrates. The *ampliconTraits* package (53) likewise revealed functional traits differentially associated with substrates of different nematode species (Table S1, Sheet S9; Fig. S10 and S11). For instance, categories connected to anaerobic, spiral-shaped, and gliding bacteria were enriched in *C. elegans* samples (Fig. S10; Table S1, Sheet S9). Conversely, halophilic and psychrotolerant categories were enriched in *C. inopinata* substrates (Fig. S11; Table S1, Sheet S9). Taken together, these analyses reveal that *C. elegans* substrates are associated with distinct, more diverse bacterial communities compared to those of its sister species, *C. inopinata*.

### Plant identity is a major driver of fig microbial diversity

**Fig 4:**
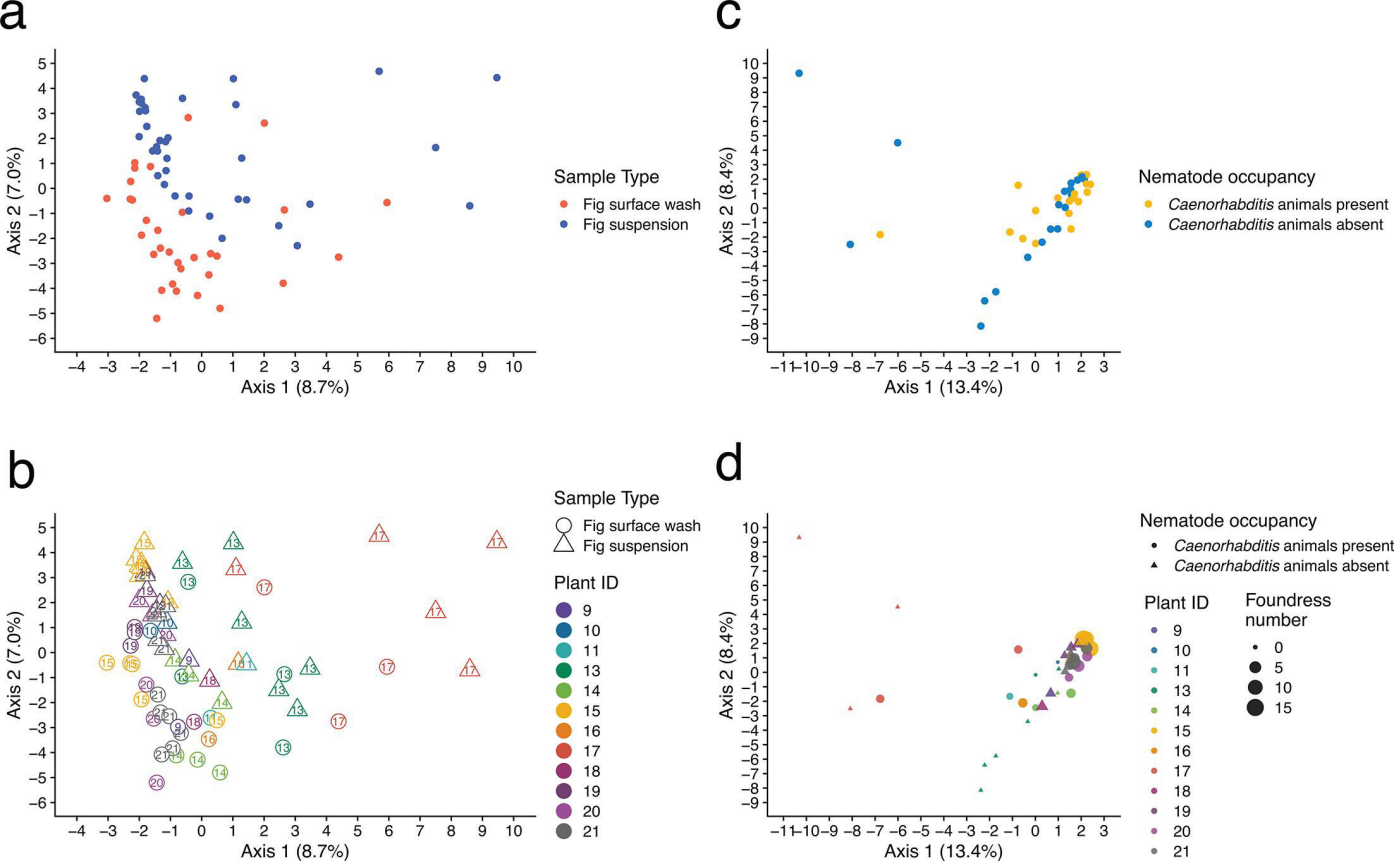
Ordination of *C. inopinata* substrate samples. (**a and b**) Microbial communities can be distinguished among: (**a**) fig surface washes and suspensions and (**b**) figs from different individual plants. Principal coordinate analysis using Aitchison distances reveals distance gradients among sample types. Each point represents a sample. Panels **a** and **b** reveal the same ordination (including both fig surface wash and fig suspension samples), with different features emphasized in the two panels. (**c and d**) A single ordination (distinct from that shown in panels **a** and **b**) including only fig suspension samples reveals (**c**) figs harboring nematodes or not and (**d**) figs harboring variable numbers of foundress pollinating wasps. Principal coordinate analysis using Aitchison distances reveals distance gradients among sample types. Each point represents a sample. Panels (**a**) and (**b**) show the same ordination. Panels (**c**) and (**d**) show a single ordination (distinct from that shown in panels **a** and **b**) including only fig suspension samples.

After comparing substrates associated with *C. elegans* and *C. inopinata* nematodes, we then considered the potential drivers of microbial diversity among our fig samples. We included plant identity, nematode occupancy, foundress wasp number, and fig preparation method (surface wash/fig suspension) as potential predictors of inter-sample microbial diversity (Fig. 4). Particularly, different plants harbor variable genotypes and environmental conditions, which are expected to potentially drive differences in microbial communities among figs. Consistent with this, individual plant identity had the largest impact on beta-diversity among the factors considered (Fig. 4b; pseudo-*R*^2^ = 0.32). This pattern remained even when figs from plant #17 (which was dominated by late-stage male figs) were removed (PERMANOVA *P* < 0.001; *F* = 2.02; pseudo-*R*^2^ = 0.33). In addition, within-sample microbial diversity varied significantly across individual plants (Fig. S13; ANOVA *P* < 0.001, *F* = 4.8–11.0 for three measures of α-diversity: number of ASV, Phylogenetic diversity, and Shannon diversity). Thus, variation among individual plants is a key factor driving microbial differences within and among *F. septica* fig samples.

### Fig exteriors and fig interiors harbor distinct microbial communities

Interior and exterior fig microbial communities are expected to be distinct because only fig interiors harbor pollinating wasps and their associated nematodes. Interior communities are expected to harbor microbes directly associated with *C. inopinata*, as previous field studies have shown that *C. inopinata* thrives in the fig interior and not on its external surface (23). Here, we use fig surface wash samples to capture exterior communities, and we use fig suspension samples to capture interior communities (although such samples will also likely include taxa from fig surfaces as well). The prevalence metrics discussed above suggest differences among the communities of surface washes and fig suspensions (Fig. 2). Consistent with this, microbial communities among fig surface washes and fig suspensions are distinct (Fig. 4a; PERMANOVA *P* = 0.001; *F* = 4.9; pseudo-*r^2^* = 0.050; Table S1, Sheet S10). This result was not impacted after accounting for the non-independence of figs sampled from the same plant (PERMANOVA *P* = 0.001; *F* = 3.7; pseudo-*r^2^* = 0.050; Table S1, Sheet S10). Comparisons of ASV abundances reveal 11 ASVs (Fig. S14), 12 genera (Fig. S15), and six families (Fig. S16) with significant and robust differences in abundance between fig surface washes and fig suspensions (Table S1, Sheet S11). Families enriched on fig surface washes included *Beijerinckiaceae* and *Microbacteriaceae*. Families enriched in fig suspensions included *Rhodobacteraceae*, *Xanthobacteraceae*, and *Enterobacteriaceae* (Fig. S16; Table S1, Sheet S11). However, fig surface washes and fig suspensions revealed no differences in within-sample α-diversity across three measures (Fig. S17; Wilcoxon rank-sum test *P* = 0.29 for number of ASV, *P* = 0.69 for Shannon diversity, and *P* = 0.07 Faith’s phylogenetic diversity). This result was not impacted after accounting for the non-independence of figs sampled from the same plant (Fig. S26). Additionally, co-occurrence analyses were performed to reveal associations between community members (see Materials and Methods section). Across 66 genera associated with the fig samples, 820 significant pairwise associations were detected (Table S1, Sheet S12). The vast majority of these were positive associations (751 genus pairs; 92%), with negative associations being relatively few and weak in comparison (69 genus pairs; 8%; Fig. S18). A community assembly analysis was performed to understand the mechanisms influencing microbiome composition. The iCAMP package models community assembly based on patterns of phylogenetic composition among samples (54). Within this framework, stochastic processes appear to dominate community assembly processes in our fig samples (70% of bin processes; Fig. S19), with drift being the most common mode of community assembly.

Few differences in community assembly were detected among fig surface washes and suspensions, although fig surface communities were found to be slightly more impacted by dispersal limitation than fig suspensions (28% vs 21% of bin processes; Fig. S19). Regardless, taken together, these results reveal distinct microbial species composition among fig exteriors and fig interiors, despite their similar levels of within-sample microbial diversity.

### Nematodes have little impact on fig microbial communities

While collecting samples for this study, we also noted the presence of *Caenorhabditis* nematodes in each fig. Here, we focused on internal fig suspensions as *C. inopinata* nematodes thrive inside figs (23). Surprisingly, after taking into account variation among individual plants, *Caenorhabditis* nematode occupancy and foundress wasp number have no detectable impact on microbial community composition (Fig. 4c and d; PERMANOVA *P* = 0.89; *F* = 0.68; pseudo-*r^2^* = 0.017; Table S1, Sheet S10). This result was not impacted after accounting for the non-independence of figs sampled from the same plant (PERMANOVA *P* = 0.74; *F* = 0.80; pseudo-*r^2^* = 0.023; Table S1, Sheet S10). Consistent with this, nematode occupancy revealed little impact on within-sample α-diversity (Fig. S20a; Wilcoxon rank-sum test *P* = 0.59 for number of ASV, 0.51 for Shannon diversity, and 0.67 Faith’s phylogenetic diversity). This result was not impacted after accounting for the non-independence of figs sampled from the same plant (Fig. S27). However, one ASV revealed significant and robust differential abundance among figs with and without *Caenorhabditis* nematodes (assigned to the genus *Ochrobactrum*; log_2_ fold change = 1.074 higher in figs with nematodes; Wald test, FDR-adjusted *P* = 0.031; Fig. S21). No ASVs (or other taxa regardless of rank) revealed significant and robust differential abundance among figs with and without *Caenorhabditis* nematodes. Of taxa that did not pass the ANCOM-BC2 (55) sensitivity analysis are considered, 70 ASVs, 40 genera, and 23 families were differentially abundant among fig suspensions with and without *Caenorhabditis* nematodes (Table S1, Sheet S13; Wald test, FDR-adjusted *P* < 0.05). Thus, while nematodes may have some influence on a few individual microbial taxa, they appear to promote little observable impact on microbial composition and within-sample diversity.

### Fig microbial diversity is negatively correlated with foundress wasp number

Figs can be pollinated by more than one wasp, and foundress number can have profound influences on the fig microenvironment. For instance, as foundress number increases, wasp progeny sex ratios become less biased (56–59). In addition, as foundress number increases, the probability of fig nematode occupancy increases (23), likely because nematodes travel on foundress pollinators (23, 60). Indeed, figs harboring nematodes tended to have more foundress wasps (Fig. S22). Because of these effects of foundress number on fig biology, it was natural to suspect that this could also impact fig microbial diversity. Indeed, foundress number is negatively correlated with within-sample phylogenetic α-diversity (Faith’s phylogenetic diversity; asymptotic regression model *P* = 0.00045, *A_sym_*= 3.03; *R_0_* = 2.5 × 10^−11^; *lrc* = −0.64) and the number of ASVs (asymptotic regression model *P* = 0.029, *A_sym_*= 2.3; *R_0_* = 7.2; *lrc* = −0.049; Fig. S20b). However, no significant relationship between within-sample Shannon diversity and foundress number was detected (an asymptotic model fit did not reach convergence; Fig. S20b). Additionally, samples with high pollinator burdens tend to cluster in ordination space (Fig. 4d). Thus, while nematode occupancy has little impact on fig microbial diversity, wasp pollinators appear to have a larger influence on these communities.

## DISCUSSION

### The microbial community of *Ficus septica* figs

Here, we used high-throughput metabarcoding to understand the microbial communities associated with *Ficus septica* figs. We identified over 2,000 ASVs across over 300 genera and nearly 200 families. Among these taxonomic groups, *Methylobacterium/Methylorubrum* species were found in nearly all of our fig samples. Notable for being able to grow on single-carbon substrates such as methanol or methylamine, these species have been frequently isolated from plants (47, 61). Moreover, *Methylobacterium/Methylorubrum* species have been shown to be beneficial to plant hosts—*Methylobacterium mesophilicum* can rescue urease mutants in soybean (61), and certain strains of *Methylobacterium extorquens* can increase the germination rate of sugarcane (62). *Sphingomonas* was another highly prevalent *Alphaproteobacteria* genus in our fig samples. *Sphingomonas* species are likewise commonly found in plant substrates (63) and also promote growth in tomatoes (64) and *Arabidopsis* (65). In addition, *Allorhizobium*-related species (48) were also highly prevalent in our fig samples. These bacteria are almost exclusively plant-associated, but they are also often plant pathogens (48). *Kineococcus* species were also abundant in our samples; these *Actinobacteria* have also been isolated from diverse substrates including soil (66), desert sand (66), angiosperm roots (67), and human-built environments (68). *Acinetobacter* species were present in all fig suspension samples (Fig. S5). Notable because of their capability to promote opportunistic infection in humans, these bacteria have also been isolated from diverse environments, including soil, water, and arthropods (69, 70). Other relatively dominant genera in our samples, such as *Spirosoma* (71) and *Chryseobacterium* (72), have also been isolated from a diverse array of similar environments, including soil, roots, and other plant-associated substrates. Taken together, the microbial community of *Ficus septica* harbors taxa that have been previously shown to be plant-associated (some with demonstrated plant-beneficial effects) or have been otherwise isolated from diverse habitats.

### The microbes of *Ficus* species and their associated wasps

Other studies have characterized fig microbial diversity—how do our results compare? While there is some overlap with other studies, the microbial communities of *Ficus septica* appear largely divergent from those reported in *Ficus hirta* and *Certaosolen* and *Eupristina* fig wasps. Liu et al. examined male and female *Ficus hirta* syconia (73). Their samples were dominated by *Wolbachia*, *Ralstonia*, and *Burkholderia* species (73). In our study, only two samples harbored *Wolbachia*, and no samples harbored *Ralstonia* or *Burkholderia*. Moreover, in our study, 11 (out of 12) male figs revealed no *Wolbachia* ASVs. It is unclear why so few *Wolbachia* reads were found in our samples. Future work directly characterizing pollinating *Ceratosolen* and parasitic *Philotrypesis* wasps associated with *F. septica* figs (23) would be more likely to discover wasp-associated endosymbionts. Two other studies examined the bacterial communities associated with fig wasps. A study examining *Ceratosolen* fig wasps (including those associated with *Ficus hispida*, known to harbor *Caenorhabditis* nematodes [74]) reported samples dominated by *Proteobacteria* (including both Gammaproteobacteria and Alphaproteobacteria), *Firmicutes*, and *Bacteroides* (20). Another study examined *Eupristina* fig wasps associated with *Ficus altissima* and *Ficus microcarpa*. Here, the dominant genera included *Propionibacterium*, *Streptococcus*, *Acinetobacter*, *Staphylococcus*, and *Klebsiella* (19). Two of these (*Acinetobacter* and *Klebsiella*) were also common in our samples. Additionally, these other studies do not report many of the most common taxa in our samples, including *Methylobacterium*, *Allorhizobium*, and *Sphingomonas*. Additionally, although other studies have looked at the fungal communities of figs (15, 19), our data cannot speak to the extent and constituents of fungal diversity in these samples. Regardless, it is then possible that there is great diversity in the microbial communities across *Ficus* fig microcosms.

### The microbes of *C. inopinata* and *C. elegans* substrates

A major goal of this work was to discover the microbes associated with the bacterivore *C. inopinata* to enable comparisons with the *C. elegans* model system. To this end, we directly compared the microbes of *C. elegans*-associated substrates with the *F. septica* fig substrates of *C. inopinata* (Fig. 2 and 3). Like *C. elegans*, substrates associated with *C. inopinata* were rich in *Proteobacteria* and were associated with ASVs in the *Actinobacteria* and *Bacteroides* phyla (Fig. 2 and 3). More specifically, out of 26 families found to be highly prevalent (>80%) in at least one sample type, nine such families were highly prevalent across all sample types (Fig. 2). These included families in the classes *Alphaproteobacteria* (*Acetobacteraceae*, *Beijerinckiaceae*, *Rhizobiaceae*, and *Sphingomonadaceae*), *Gammaproteobacteria* (*Comamonadaceae* and *Xanthomonadaceae*), *Actinobacteria* (*Microbacteriaceae* and *Nocardioidaceae*), and *Bacteroida* (*Spirosomaceae*; Fig. 2). Despite this, the microbiomes of fig substrates and *C. elegans*-associated substrates were clearly compositionally distinct (Fig. 3). Indeed, some families were far more abundant in fig samples (*Deinococcae* and *Kineosporiaceae*; Fig. S8), whereas others were more abundant in *C. elegans* substrates (*Rhodanobacteraceae* and *Pirellulaceae*; Fig. S8). Taken together, these seemingly radically divergent ecological niches (rotting plant detritus and fresh figs) reveal surprising overlap in their microbial communities while harboring clear differences in their most prevalent and abundant constituents. Previous studies have noted divergent microbial communities associated with different *Caenorhabditis* species (36, 75), suggestive of species-specific ecological niches. The divergent *C. inopinata*-associated microbial community is consistent with the notion that microbial environments are important drivers of species divergence. However, vastly more ecological work is required to determine whether *Caenorhabditis* species occupancy and divergence are driven by the composition of microbial environments. Additionally, other studies have examined the microbial communities of nematodes themselves (apart from their associated substrates) (36). Here, we only examined substrates (either fig suspensions or surface washes), so we are unable to disentangle nematode-associated microbes from fig-associated microbes *per se*. Future work examining nematodes isolated from their environmental sources will be able to identify such taxa.

### Fig microbes, ecology, and the genetics of model systems

Here, we describe the bacteria associated with *C. inopinata* substrates. It may not be immediately obvious how this work can inform genetic hypotheses related to the *C. elegans* model system. *C. elegans* has been the subject of intensive genetic research for over 50 years (31), and it has proven instrumental in advancing our understanding of myriad subjects, including programmed cell death, small RNA biology, and cell signaling (76). Despite this, only 43% of the 19,984 genes of the *C. elegans* genome have a known phenotypic function (Fig. S23) (32). As conventional genetic approaches (such as forward and reverse screens in standard laboratory conditions) yield diminishing returns, new background information must be generated to inform novel genetic hypotheses. Here, we suggest that comparative genomics, together with an ecology-informed genetics, can drive the discovery of new hypotheses. Comparative genomics has enabled myriad discoveries, and patterns of genomic conservation and divergence can reveal functional genomic regions (77–79). And, as the vast majority of *C. elegans* genetic work has been performed in laboratory conditions far removed from this animal’s ecological context, genetic experiments informed by the natural environment of *C. elegans* will likely prove critical in enabling the discovery of novel gene functions (32). Such a comparative ecological framework will require a description (and comparison) of the natural environments of the organisms involved. This is exactly the kind of work shared here—a comparison of the critical microscopic and biotic constituents of the environments of two *Caenorhabditis* sister species.

For further clarity on this point, we relate a specific hypothetical example. For instance, *C. elegans* harbors hundreds of genes encoding G-protein coupled receptors (GPCRs) and F-box proteins. GPCRs are frequently implicated in the transduction of environmental sensory information (80), whereas F-box proteins have been implicated in nematode innate immune responses (39). From the vantage point of comparative genomics, it is clear that these genes are part of dynamic gene families that contract and expand across phylogeny (81). Indeed, *C. elegans* has more than triple the number of GPCRs compared to *C. inopinata* (1,329 vs 382 serpentine 7TM-GPCRs (30)). At the same time, as we have shown through descriptive comparisons of microbial environments, *C. elegans* populations are frequently associated with microbial communities that are far more diverse than those usually encountered by *C. inopinata* (Fig. 3; Fig. S4 to S7). Thus, *C. elegans* may harbor species-specific GPCR genes (or orthologs that *C. inopinata* does not have) that are important for sensing specific microbe-associated molecules in these diverse environments. Considering such environments also appear to harbor specific kinds of microbes (Fig. S8), these observations can then inform specific laboratory experiments to test the functional roles of such genes. That is, one provisional hypothesis might be that a novel GPCR gene facilitates the sensation of a microbe specific to the environment of *C. elegans* (or that a novel F-box gene enables an immune response to a harmful pathogen that is restricted to the diverse environment of *C. elegans*). To know if an environment (and its constituent microbes) is specific to *C. elegans*, one must examine the environments of its close relatives. Thus, the work shared here represents a key piece of background information that can enable the generation of future hypotheses in model systems genetics.

Moreover, as *C. inopinata* is the sister species of *C. elegans*, many of the genetic tools of *C. elegans* are transferable to its close relative (30). Thus, with the ecological information shared here, this work not only can inform the comparative genetics of *C. elegans*, but also be used to generate testable and ecologically informed genetic hypotheses in *C. inopinata*. Our recent work describing how *C. inopinata* responds to microbes isolated from the natural fig environment (82), performed concurrently with the work shared here, extends these ideas by showing how ecological field work can generate the raw material for novel environments in laboratory genetic experiments.

### The drivers of fig microbial diversity

Here, we found that a major driver of fig microbiome diversity is the individual plant from which a fig was picked (Fig. 4b), with one male plant in particular promoting higher within-sample microbial diversity than the others (Fig. S13). Thus, it is likely that genetic and environmental variation among individual plants is a key contributor to microbiome diversity and composition in *F. septica* figs. Genetic (83–85) and environmental (86–88) variation have been shown to be a key driver of microbiome diversity across multiple systems. There is ample genetic variation in *F. septica* (89, 90). *F. septica* also exhibits environmental plasticity across diverse environments (91). It is then unsurprising that plant of origin is a major driver of fig microbial diversity.

In addition to detecting the impacts of individual plants on microbial diversity, we showed that the microbial communities of fig interiors (suspensions) were distinct from those of fig exteriors (surface washes; Fig. 4a). As fig nematodes thrive in the *interior* lumen of the fig (12, 23), they are likely not to be associated with these microbial taxa that are restricted to the fig surface. Thus, taxa such as these might be less likely to interact with species like *C. inopinata*. However, we detected no unique genera to the fig surface, although some were enriched in abundance on the fig surface relative to fig suspensions (Fig. S15). In addition, no differences in microbial diversity among fig suspensions and fig surface washes were detected (Fig. S17). If anything, there is a trend of *increased* microbial diversity in fig suspensions compared to fig surface washes (Fig. S17). This was somewhat unexpected, as the fig lumen has strong barriers to entry (6), and we reasoned that as the fig interiors were less exposed, they might have less complex microbiomes. As this was not the case, it is possible that simply the increased biotic material in these samples (compared to surface washes) contributed to this potentially increased microbial diversity. Along these lines, fig suspensions contained nematode, wasp, and fig tissue that was not present in the fig surface wash samples. This is likely the most parsimonious explanation of the diversity observed in fig interiors.

Additionally, we observed a trend of declining microbial diversity with foundress wasp number (Fig. S20b). Foundress wasps are known to change the fig environment: they introduce pollen, promoting seed development, and they lay eggs in fig ovules. They carry nematodes and microbes that have the potential to influence the fig environment. Indeed, the probability of nematode occupancy increases as foundress number increases in these figs (23). Thus, it is perhaps unsurprising that the microbial community likewise changes with foundress number. It is unclear why microbial diversity should *decrease* with foundress number. One possibility is that bacterivorous nematode load increases with foundress number, which leads to a reduction in microbial diversity (due to microbe consumption by nematodes). However, we saw no influence of nematode occupancy on microbial diversity as such (Fig. S20a). Additionally, foundress wasps may carry bacterial taxa that dominate the fig upon colonization, leading to decreased diversity. Consistent with this, the Smith and Wilson’s Evar evenness index (92) likewise exhibits a negative relationship with foundress number (although other evenness metrics do not reveal such a relationship; Fig. S24). Alternatively, increased pollination, oviposition, or both may induce changes in the fig environment that impact microbial diversity. Regardless, future studies with a larger range of phenotypic conditions, in addition to manipulative field experiments, will be needed to disentangle these possibilities.

We also detected no impact of nematode occupancy on microbial diversity (Fig. 4c; Fig. S20a). This is surprising because *C. elegans* occupancy has been shown to have an influence on substrate communities (37), with nematode abundance promoting a reduction of diversity (and with reproductive and dispersal stage-biased populations harboring differing microbial communities [37]). It is unclear why no influence was observed—one possibility is that *C. inopinata*, despite its bacterivorous status, simply does not have a large effect on fig microbial communities. This may particularly be true if *C. inopinata* does not eat much bacteria, but rather more fungi, protists, or other particles in the fig. Indeed, the natural food source of *C. inopinata* has not yet been determined. Despite this, it is likely that the natural diet of *C. inopinata* includes bacteria, as this species descended from bacterivorous ancestors and can be reared on bacteria under laboratory conditions (30). However, it is also possible that we do not yet have the power to detect the impact of *C. inopinata* on fig microbial communities, and that analyzing more figs, obtaining more reads, and sampling a broader range of fig conditions (i.e., figs with a greater range in nematode loads or nematode stages) would reveal greater variation in fig microbial communities. Despite this, we did detect at least one ASV associated with nematode occupancy (an *Ochrobactrum* ASV; Fig. S21). Ongoing metagenomic work on individual nematodes from the field, as well as ongoing experimental work with fig-derived microbes, will be invaluable toward informing these possibilities.

### Microbial communities, complexity, and the *C. inopinata* niche

At least one study has been performed on the fig microbiome ([73]; see above). Here, they found differences in microbial composition between male and female *Ficus hirta* figs (73), but beyond this, the impact of specific bacteria (or microbial communities) on fig physiology and fitness remains an open question. However, bacterial diversity has widespread impacts on nutrient uptake, disease resistance, stress tolerance, and growth regulation across plants (44). Moreover, myriad bacterial species have negative impacts on plant physiology (93). It is then likely that both beneficial and deleterious microbes are included among those described here. In addition, at least one study has examined the fungal diversity among six *Ficus* species (not including *F. septica*) (15). In that report, diversity in fungal community composition across species and plant tissues (syconia, leaves, and stems) was detected (15). In addition, different *Ficus* species varied in fungal community composition (15). Our approach here did not address fungal taxa. Despite this, like bacteria, fungi have profound impacts on plant physiology (94). Moreover, fungi-bacterial interactions exist and can have cascading impacts on other community members (95). Thus, an open question is the extent of fungal diversity in *F. septica* figs and its broad impacts on community biology. Along similar lines, unicellular eukaryote diversity has not been addressed in any *Ficus* system (to the best of our knowledge), despite their presence in these communities. Regardless, the work described here is descriptive and represents a first step in characterizing functionally and physiologically relevant interactions among fig microbial community members.

In addition, we found that foundress wasp number revealed modest impacts on fig microbial community composition (Fig. 4d; Fig. S20b), while nematodes barely influenced such communities (Fig. 4c). Does this suggest that the fig microcosm, with its collection of meiofauna (including wasps and worms, among other small animals), is perhaps a poor model of ecosystem complexity? That is, if nematode occupancy does not reliably predict microbial community features, these organisms do not meaningfully interact. Indeed, our results suggest that nematodes are, at most, a minor driver of microbial communities (Fig. 4d). *C. inopinata* occupancy did impact the abundance of at least one bacterial taxon (an *Ochrobactrum* ASV, Fig. S21). However, the field data associated with our fig samples were quite coarse—we merely assayed *Caenorhabditis* nematode occupancy. It is possible that various other features that we did not properly assess here impact microbial communities. For instance, there were a number of features that we did not measure at all, such as nematode abundance and nematode stage distribution. In addition, a novel nematode species associated with *Ficus septica* was recently described (*Aphelenchoides epiphyticus* [96]), and detailing its occupancy and abundance in tandem with *C. inopinata* may inform impacts on microbial communities. Moreover, our fig data were largely biased toward young, female figs without parasitic wasps. Thus, we were not able to properly assess the impacts of fig developmental stage, fig sex, and parasitic wasp impact on fig microbial communities. An obvious next step toward understanding the relationship between fig microbes and other fig species would be taking metrics of fig, wasp, and nematode fitness while simultaneously assessing microbial community composition. Thus, while *Caenorhabditis* nematode occupancy alone may not drive much microbial variation, there may be interactions to discover associated with other traits and phenotypes that could be measured in future studies.

Along these lines, the specific ecological role of *C. inopinata* has yet to be determined. That is, the exact nutritional resources that *C. inopinata* consumes in *F. septica* figs are not known. *Caenorhabditis* nematodes are bacterivores, in general (97). *C. inopinata* can be reared in laboratory conditions on bacterial food sources (30, 98). For these reasons, *C. inopinata* is expected to eat bacteria in the fig environment. We did find one bacterial taxon whose abundance covaried with *C. inopinata* occupancy (an *Ochrobactrum* ASV, Fig. S21). This may represent a potential food source. Ongoing studies examining the microbial communities of surface-sterilized individual nematodes from the fig environment will be invaluable toward understanding what exactly *C. inopinata* eats. However, a possibility remains that *C. inopinata* is not a bacterivore, or that *C. inopinata* consumes food beyond bacteria alone. There may be other microscopic particles in figs that *C. inopinata* consumes, which may include material derived from plant, fungal, wasp, or unicellular eukaryote material. However, this is unlikely, as *C. inopinata* fitness is increased in laboratory conditions when reared on bacteria derived from the fig environment (30, 82). The bacteria that *C. inopinata* consumes are likely to be included among those taxa described in this work, and discovering and characterizing the natural food sources of *C. inopinata* remains a line of active research.

### Implications for the biology of *C. inopinata*

*C. inopinata* is an ecologically and morphologically exceptional *Caenorhabditis* species (30). In a genus largely characterized by profound phenotypic stasis across large genetic distances (e.g., *C. briggsae* and *C. elegans* are nearly morphologically indistinguishable despite harboring genetic divergence comparable to that of human and mouse [99]), the current sister species of *C. elegans* is an outlier. *C. inopinata* is large in size, grows slowly, and thrives in a novel environment (23, 30, 98, 100). Here, we set out to better understand this microenvironment to better inform how its ecological context may influence its phenotypic divergence. While we did not detect any impacts of nematode occupancy on fig microbial composition (Fig. 4c) or within-sample diversity (Fig. S20a), differentially abundant ASVs by nematode occupancy were detected (Fig. S21). Indeed, ASVs were detected that were both enriched (e.g., ASVs from the genera *Ochrobactrum* and *Stentotrophomonas*; Fig. S21) and depleted (e.g., ASVs from the genera *Sphingobacteriales* and *Spirosoma*; Fig. S21). Thus, these taxa *may* interact with *C. inopinata* in some way—either as food, pathogens, or otherwise beneficial or detrimental species. These taxa were also found among the *C. elegans* substrates we examined, and these two genera connected to differentially abundant ASVs in figs with *Caenorhabditis* nematodes (*Ochrobactrum* and *Stentotrophomonas*) have been included in the CeMbio resource, a simplified, standardized natural *C. elegans* microbiota for experimental studies (101). *Ochrobactrum* species, in particular, have been frequently associated with the *C. elegans* microbiome (85, 102). Additionally, this species has also been found in association with natural *C. briggsae* and *C. remanei* samples (36). The findings here suggest that members of this bacterial genus may represent a highly conserved and ancient constituent of *Caenorhabditis* nematode microbiomes. This supports the notion that these taxa represent the natural food of (or may otherwise interact with) *C. inopinata*. Alternatively, these patterns may emerge via indirect consequences of nematode occupancy via their potential impacts on other community members. Conversely, these presumptive nematode-responsive taxa may represent false positives. Additionally, we also detected a decline of microbial diversity with foundress wasp number (Fig. S20b). As the probability of worm occupancy increases with foundress number (Fig. S22) (i.e., nematodes travel on wasps) (12, 23), it is reasonable to suspect that within-fig nematode population sizes increase as foundress number increases. Thus, nematodes may be more likely to be exposed to microbial communities associated with crowded figs. Nematodes may experience more frequent (or stronger) selection in these environments, and microbial taxa enriched in high foundress number figs may represent better proxies for a core microbial community of *C. inopinata*. For instance, *Enterobacterales* bacteria were highly abundant in a fig with 19 foundresses, suggesting that this family may interact more frequently with *C. inopinata*. Indeed, microbes frequently associated with *C. elegans* in nature can modulate fitness-related traits in laboratory conditions (36, 37). Continued sampling of high foundress number figs, the sequencing of individual *C. inopinata* nematodes isolated from natural environments, together with targeted microbial culture strategies, will build out the resources required to understand the novel environment of *C. inopinata* and how it influences its divergent phenotypes.

### Caveats

Limitations to this work exist. As mentioned above, although the goal of this study is to characterize the microbial communities associated with *C. inopinata*, it cannot delineate the *C. inopinata* microbiome as such. As we used whole fig suspensions, we cannot distinguish between microbes that directly interact with *C. inopinata* from those that do not. Indeed, many figs were also occupied by wasps and fungi, and some were occupied by other animals (such as mites, non-wasp insect larvae, and ants). Of course, our samples were dominated by fig tissue. Thus, these results are best interpreted as a description of the *F. septica* fig community, and not one of the microbiomes of a specific fig community member. To understand nematode microbiomes, we will need to sequence individual nematodes. Surface-sterilized nematodes have been used to potentially interrogate the gut microbiome (30, 36), which is of broad interest. Connected to this is the large number of organellar reads observed in our data (Fig. S2). High fractions of organellar reads have been observed before in plant amplicon sequencing data (up to 92% in kelp samples) (103). Presumably, these are derived from the host and other eukaryotic community members. This suggests that these results may need to be approached with caution, as rarer microbial taxa were perhaps not detected, given that most reads were covered by uninformative non-bacterial sources. Yet, future studies using alternative library preparation methods to limit organellar DNA amplification (104) may prove useful in saving costs due to the generation of such uninformative sequences (and to mitigate any biases these sequences may introduce). We also found that individual plants are a major driver of fig microbial diversity (Fig. 4B). As many of our analyses assumed independence among individual figs, some of our ecological inferences may likewise instead be driven by variation at the individual plant level. Additionally, our experience in analyzing these results leads us to suspect that taxonomy has an outsized influence—results and interpretation can vary depending on the taxonomic system implemented. There may be similarities between our work and previous studies that we have overlooked due to differences in taxonomic system; likewise, there may have been important differences among studies that we may have overlooked for the same reason. Finally, metabarcoding provides little information regarding the biochemical functional roles of microbial community members. Future work assembling whole bacterial genomes associated with these figs will be required to delineate functional roles among community members to understand if they likewise covary with nematode occupancy.

### Concluding thought

Here we described the microbes of the natural environment of the sister species of a key model genetic system. This sets the stage for future work aimed at generating hypotheses regarding the functions of uncharacterized genes and the evolution of ecological divergence. The promise of the *C. inopinata* system lies in the potential to test molecular genetic hypotheses regarding the causes of ecological divergence. To even begin to do this, we must properly describe its divergent environment. That is what we have done here.

## MATERIALS AND METHODS

### Sampling

*Ficus septica* figs were sampled in Taipei, Taiwan, on August 27, 2019. Figs were then stored at 4°C. One to two days after picking (in the John Wang lab at Academia Sinica), each individual fig was placed in a sterile petri dish and washed with 500 μL of sterile M9 buffer. This surface wash was then transferred to a sterile 1.5 mL Eppendorf tube and stored at −80°C. Then, that same fig was sliced into four pieces in 4 mL of sterile M9 buffer. The fig and suspension were then observed to assess: the approximate fig developmental stage (using the scheme of [105]); the number of foundress wasps; the presence of *Caenorhabditis* nematodes (determined by pharynx morphology and noted by the “worms_present” column in Table S1, Sheet S2); the presence of other nematode morphotypes; the presence of reproductive stage or dauer-like *Caenorhabditis* nematodes; and the presence of non-pollinating wasps. Then, 500 μL of fig homogenate was transferred to a sterile 1.5 mL Eppendorf tube and stored at −80°C.

### DNA extraction, library preparation, and sequencing

For DNA extraction, samples (250 μL) were added to a 2 mL screw-cap tube with 750 μL of Qiagen PowerBead Solution, 60 μL of Qiagen SL Solution, and approximately 30 1.4 mm ceramic beads. The samples were homogenized at 1,600 rpm using an MP Biomedicals FastPrep-96 for 2.5 min. Samples were then digested overnight (~16 h) with 10 μL of Proteinase K (20 mg/mL) at 56°C. Samples were extracted using the MagAttract PowerSoil DNA Kit (Qiagen) following the manufacturer’s protocol on a KingFisher Flex (Thermo Scientific) and eluted into 100 μL. The library was prepared using the Earth Microbiome Project 16S primer set (106) (forward primer 515F: GTGYCAGCMGCCGCGGTAA; reverse primer 806R: GGACTACNVGGGTWTCTAAT; Table S1, Sheets S1 and S2). PCR was performed with Platinum II Hot-Start 2× PCR Master Mix (Invitrogen). A SimpliAmp thermal cycler (Applied Biosystems) was used with the following cycling conditions: initial denaturation at 94°C for 2 min; 35 cycles of denaturation at 94°C for 15 s, annealing at 54°C for 15 s, extension at 68°C for 7 s; and a final extension step at 68°C for 3 min. PCR amplification was confirmed through visualization on a 2% agarose gel. Samples were then purified and normalized to approximately 1.25–1.50 ng/μL using the Just-a-Plate PCR Normalization kit (Charm Biotech). A volume of 10 μL from each sample was pooled before being purified and concentrated using a volume of 1.2× of homemade SPRI beads. The final library was checked for concentration using a Qubit 1× dsDNA High Sensitivity Kit (Thermo Fisher Scientific). Purity and fragment size were confirmed using a Bioanalyzer High Sensitivity DNA Kit (Agilent Technologies). Multiple negative and positive controls were prepared throughout the extraction and library preparation process: three M9 buffer negative controls (i.e., samples that went through the entire experimental workflow *sans* fig substrate material); two DNA extraction negative controls; one DNA extraction positive control; two PCR negative controls; and two PCR positive controls (ZymoBIOMICS Microbial Community DNA Standard). Paired-end 150 bp reads were then generated with the Illumina MiSeq platform.

### Read processing, ASV assignment, microbial abundance, and statistical inference

Raw sequence data were vetted for quality with FastQC (107). *C. elegans* substrate sequences (36, 37) were retrieved from GenBank (accession numbers can be found in Table S1, Sheet S5). Because one study (37) inferred microbial communities with single-end sequencing, only forward reads were included for the joint analysis of *C. elegans* substrate and *C. inopinata*-associated fig samples. For analyses examining fig samples alone, both forward and reverse reads were included. ASVs and their counts were resolved with DADA2 (108) implemented in the Qiime2 package (version 2024.10.1) (109). For the joint analysis of *C. elegans* substrates and fig samples, reads were imported into Qiime2 using *qiime tools import* (options --type 'SampleData[SequenceWithQuality]'). For the analysis of fig sample reads alone, reads were imported into Qiime2 using *qiime tools import* (options --type 'SampleData[PairedEndSequencesWithQuality]' --input-format CasavaOneEightSingleLanePerSampleDirFmt).

DADA2 was run within Qiime2 using *qiime dada2 denoise-single* (options --p-trunc-len 150; including *C. elegans* samples) or *qiime dada2 denoise-paired* (options --p-trunc-len-f 150 --p-trunc-len-r 150; fig samples alone). Pre-formatted full-length sequences and taxonomic assignments from the SILVA database were retrieved from the Qiime2 website (version 2022.2; https://data.qiime2.org/2022.2/common/silva-138-99-seqs-515-806.qza and https://data.qiime2.org/2022.2/common/silva-138-99-tax-515-806.qza) (51, 110, 111). Taxonomic identities were assigned to ASVs using *qiime feature-classifier classify-consensus-vsearch* (with default parameters) (112). In addition, a phylogenetic tree was generated with representative ASV sequences using *qiime phylogeny align-to-tree-mafft-fasttree* (using default parameters) (113, 114). The feature table, taxonomy, and phylogeny were then converted from the QZA file format to CSV, TSV, and NWK formats, respectively, using *qiime tools export* with using default parameters for downstream analysis.

Statistical analyses were performed in R (115) with the aid of the *phyloseq* package (116, 117). Because DADA2 was run with default parameters, singleton ASVs were excluded in all analyses (i.e., ASVs must have had a read count >1 to be included in downstream analyses) (108). For downstream analyses, ASVs associated with mitochondria and chloroplasts were excluded. To do this, the *subset_taxa*() function in the *Phyloseq* R package was used with the options "!Family %in% "Mitochondria" & !Order %in% "Chloroplast"". In addition, ASVs defined in control samples were also excluded. These ASVs were included for a visualization that included controls (Fig. S12). Details of these controls are described in the section “DNA extraction, library preparation, and sequencing”. To exclude ASVs observed in control samples, the *prune_samples* function in the *Phyloseq* R package was used (in the form "*prune_samples*(!(*sample_names*(phyloseq_object) %in% c(vector_of_control_sample_ids), phyloseq_object)"). Ordinations (Fig. 3 and 4; Fig. S12) were performed using Aitchinson distances (118). Feature table (i.e., ASV count) data were center log-ratio (CLR) transformed for ordinations using the *transform* function (with option transform="clr") in the *microbiome* R package. Ordinations were performed with the *ordinate* function (with option method="RDA") in the *phyloseq* R package and were subsequently visualized (Fig. 3 and 4; Fig. S12).

For PERMANOVA, distance matrices were constructed from CLR-transformed abundances using the *phyloseq* function *distance* (with options method = "euclidean"). PERMANOVA was then performed with the *vegan* function *adonis2*, using the formula (microbiome distance matrix ~ “nematode species” + “study” + “substrate type”) for the joint analysis of *C. elegans* substrates and fig samples. For the analysis of all fig samples alone, this model formula was used: (microbiome distance matrix ~ “surface or interior” + “plant label” + “foundress number” + “worms present”). As nematodes thrive inside figs (and not on their exterior surface) (23), a PERMANOVA was also performed on fig suspension samples only, using the model formula (microbiome distance matrix ~ “plant label” + “foundress number” + “worms present”). The resultant summaries of these models can be found in the supplemental material (Table S1, Sheets S6 and S10).

For differential abundance analyses, the *ANCOM-BC2* R package (55), which attempts to account for the compositional nature of microbiome data in differential abundance estimation, was used. The *ancomb2* function (with options p_adj_method="BH", global=TRUE, pairwise=TRUE, and dunnet=TRUE) was used to identify differentially abundant taxa among various sample types. Differential abundance analyses were performed on all taxonomic ranks, and the *tax_glom* function in the *phyloseq* R package was used to agglomerate taxa of the same type. CLR-transformed abundances were also used for visualizations of differential abundance of various taxa (Fig. S8, S14 to S16 and S21).

For measures of within-sample alpha diversity (Fig. S4 to S7, S13, S17 and S20a), two samples with low non-organellar read counts were removed (samples GW20, GW34). The number of unique ASVs per sample was inferred with the *phyloseq* function *estimate_richness* (option *measures* = "Observed"). Shannon diversity was estimated with the *phyloseq* function *estimate_richness* (option *measures* = "Shannon"). Faith’s phylogenetic diversity was calculated using the *picante* function *pd* (with default parameters), which sums the branch lengths associated with the subset of the phylogenetic tree (inferred with Qiime2 as described above) associated with a given sample. To examine the impact of rarefaction on within-sample alpha diversity, reads were rarified to 9,056 reads per sample with the *phyloseq* function *rarefy_even_depth* (with options *rngseed* = *N*, *replace* = FALSE). Reads were rarefied 1,000 times (each with different random seeds *N*), and the average diversity metric for each sample across the 1,000 rarefied replicates was determined and plotted (Fig. S6 and S7).

Co-expression analyses (i.e., the inference of positively or negatively associated microbial taxa among samples) were performed with fig samples via the *microeco* (119) and *mecoentcomp* (120) packages. Pearson correlation-based networks were performed with the functions *trans_network$new* (with options *cor_method* = "spearman", *filter_thres* = 0.001); *cal_network* (with options *COR_p_thres* = 0.05, *COR_cut* = 0.05, *COR_p_adjust* = "FDR"); and *cal_module* (with the option *method* = "cluster_fast_greedy"). Correlation coefficients among genus pairs were extracted from networks and plotted in Fig. S18.

Functional analyses were performed with FAPROTAX (52) and the *amplicontraits* database (53, 121). For FAPROTAX (version 1.2.11), the script *collapse_table.py* was used on feature tables with default parameters, resulting in output that reports the proportion of ASV belonging to each functional group in each sample (52; Fig. S9). For *amplicontraits*, ASV sequences are queried against a database to connect ASVs with phenotypic information. Here, this phenotypic information was then used to determine differential abundances among phenotypic classes with the *ANCOM-BC2* R package (55) as described above (*ANCOM-BC2*-inferred log fold changes are reported in Fig. S10 and S11). All data and code associated with this study have been deposited in GitHub (https://github.com/gcwoodruff/F_septica_16S_microbial_ecology_2024).

The following R packages were used in this study: *ANCOMBC* (55); *ape* (122); *cowplot* (123); *data.table* (124); *dplyr* (125); *DT* (126); *effsize* (127); *ggforce* (128); *ggmap* (129); *ggplot2* (130); *ggpubr* (131); *iCAMP* (54); *lemon* (132); *meconetcomp* (120); *microbiome* (133); *microbiomeutilities* (134); *patchwork* (135); *phyloseq* (116); *picante* (136); *RColorBrewer* (137); *reshape2* (138); *vegan* (139); and *UpSetR* (140).

## Data Availability

Data and code associated with this study have been deposited in GitHub (https://github.com/gcwoodruff/F_septica_16S_microbial_ecology_2024). FASTQ files have been submitted to the NCBI Sequence Read Archive (http://www.ncbi.nlm.nih.gov/sra) under BioProject ID PRJNA1170329.

## SUPPLEMENTAL MATERIAL

Supplemental figures spectrum.03013-24-s0001.pdf Figures S1 to S27.

Supplemental tables and sheets spectrum.03013-24-s0002.xlsx Table S1 and Sheets S1 to S13.

ASM does not own the copyrights to Supplemental Material that may be linked to, or accessed through, an article. The authors have granted ASM a non-exclusive, world-wide license to publish the Supplemental Material files. Please contact the corresponding author directly for reuse.

## References

[1] Hortal J, de Bello F, Diniz-Filho JAF, Lewinsohn TM, Lobo JM, Ladle RJ. 2015. Seven shortfalls that beset large-scale knowledge of biodiversity. Annu Rev Ecol Evol Syst 46:523–549. doi:10.1146/annurev-ecolsys-112414-054400

[2] Morales-Castilla I, Matias MG, Gravel D, Araújo MB. 2015. Inferring biotic interactions from proxies. Trends in Ecology & Evolution 30:347–356. doi:10.1016/j.tree.2015.03.014 25922148

[3] Hanson PJ, Walker AP. 2020. Advancing global change biology through experimental manipulations: where have we been and where might we go? Glob Chang Biol 26:287–299. doi:10.1111/gcb.14894 31697014 PMC6973100

[4] Borges RM. 2021. Interactions between figs and gall-inducing fig wasps: adaptations, constraints, and unanswered questions. Front Ecol Evol 9. doi:10.3389/fevo.2021.685542

[5] Su Z-H. 2008. Breakdown of the one-to-one rule in Mexican fig-wasp associations inferred by molecular phylogenetic analysis. Symbiosis 45:73.

[6] Castro-Cárdenas N, Vázquez-Santana S, Teixeira SP, Ibarra-Manríquez G. 2022. The roles of the ostiole in the fig-fig wasp mutualism from a morpho-anatomical perspective. J Plant Res 135:739–755. doi:10.1007/s10265-022-01413-9 36264520

[7] Verkerke W. 1989. Structure and function of the fig. Experientia 45:612–622. doi:10.1007/BF01975678

[8] Hatta SKM, Quinnell RJ, Compton SG. 2023. Pollinator attraction in the Ficus deltoidea complex: varietal specificity in a fig wasp that likes to stay close to home. Acta Oecologica 121:103939. doi:10.1016/j.actao.2023.103939

[9] Borges RM. 2015. How to be a fig wasp parasite on the fig-fig wasp mutualism. Curr Opin Insect Sci 8:34–40. doi:10.1016/j.cois.2015.01.011 32846670

[10] Janzen DH. 1979. How to be a fig. Annu Rev Ecol Syst 10:13–51. doi:10.1146/annurev.es.10.110179.000305

[11] Segar ST, Dunn DW, Darwell CT, Cook JM. 2014. How to be a fig wasp down under: the diversity and structure of an Australian fig wasp community. Acta Oecologica 57:17–27. doi:10.1016/j.actao.2013.03.014

[12] Van Goor J, Kanzaki N, Woodruff G. 2023. How to be a fig nematode. Acta Oecologica 119:103916. doi:10.1016/j.actao.2023.103916

[13] Weiblen GD. 2002. How to be a fig wasp. Annu Rev Entomol 47:299–330. doi:10.1146/annurev.ento.47.091201.145213 11729077

[14] Su Z-H, et al.. 2008. Breakdown of the one-to-one rule in Mexican fig-wasp associations inferred by molecular phylogenetic analysis, p 73–81. In Symbiosis. Philadelphia, PA.

[15] Martinson EO, Herre EA, Machado CA, Arnold AE. 2012. Culture-free survey reveals diverse and distinctive fungal communities associated with developing figs (Ficus spp.) in Panama. Microb Ecol 64:1073–1084. doi:10.1007/s00248-012-0079-x 22729017

[16] Jandér KC. 2015. Indirect mutualism: ants protect fig seeds and pollen dispersers from parasites. Ecol Entomol 40:500–510. doi:10.1111/een.12215

[17] Sugiura S, Yamazaki K. 2004. Moths boring into Ficus syconia on Iriomote Island, South‐western Japan. Entomological Science 7:113–118. doi:10.1111/j.1479-8298.2004.00056.x

[18] Jauharlina J, Lindquist EE, Quinnell RJ, Robertson HG, Compton SG. 2012. Fig wasps as vectors of mites and nematodes. African Entomology 20:101–110. doi:10.4001/003.020.0113

[19] Dong Y, Zhang Z-R, Mishra S, Wong A-N, Huang J-F, Wang B, Peng Y-Q, Gao J. 2022. Diversity and metabolic potentials of microbial communities associated with pollinator and cheater fig wasps in fig-fig wasp mutualism system. Front Microbiol 13:1009919. doi:10.3389/fmicb.2022.1009919 36466640 PMC9715610

[20] Li J, Wei X, Huang D, Xiao J. 2022. The phylosymbiosis pattern between the fig wasps of the same genus and their associated microbiota. Front Microbiol 12:800190. doi:10.3389/fmicb.2021.800190 35237241 PMC8882959

[21] Liu C, Yang D-R, Compton SG, Peng Y-Q. 2013. Larger fig wasps are more careful about which figs to enter – with good reason. PLoS One 8:e74117. doi:10.1371/journal.pone.0074117 24086315 PMC3781092

[22] Susoy V, Herrmann M, Kanzaki N, Kruger M, Nguyen CN, Rödelsperger C, Röseler W, Weiler C, Giblin-Davis RM, Ragsdale EJ, Sommer RJ. 2016. Large-scale diversification without genetic isolation in nematode symbionts of figs. Sci Adv 2:e1501031. doi:10.1126/sciadv.1501031 26824073 PMC4730855

[23] Woodruff GC, Phillips PC. 2018. Field studies reveal a close relative of C. elegans thrives in the fresh figs of Ficus septica and disperses on its ceratosolen pollinating wasps. BMC Ecol 18:26. doi:10.1186/s12898-018-0182-z 30129423 PMC6102938

[24] Chang CC, Turner BL. 2019. Ecological succession in a changing world. Journal of Ecology 107:503–509. doi:10.1111/1365-2745.13132

[25] Kruger MS, Kanzaki N, Giblin-Davis RM, Greeff JM. 2021. Molecular diversity and relationships of fig associated nematodes from South Africa. PLoS One 16:e0255451. doi:10.1371/journal.pone.0255451 34375357 PMC8354458

[26] Davies KA, Ye W, Kanzaki N, Bartholomaeus F, Zeng Y, Giblin-Davis RM. 2015. A review of the taxonomy, phylogeny, distribution and co-evolution of Schistonchus Cobb, 1927 with proposal of Ficophagus n. gen. and Martininema n. gen. (Nematoda: Aphelenchoididae). Nematol 17:761–829. doi:10.1163/15685411-00002907

[27] Kanzaki N, Giblin-Davis RM, Davies K, Ye W, Center BJ, Thomas WK. 2009. Teratodiplogaster fignewmani gen. nov., sp. nov. (Nematoda: Diplogastridae) from the syconia of Ficus racemose in Australia. Zool Sci 26:569–578. doi:10.2108/zsj.26.569 19719410

[28] Poinar GO, Herre EA. 1991. Speciation and adaptive radiation in the fig wasp nematode, Parasitodiplogaster (Diplogasteridae: Rhabditida) in Panama. Rev nématol 14:361–374.

[29] Kanzaki N, Tanaka R, Giblin-Davis RM, Davies KA. 2014. New plant-parasitic nematode from the mostly mycophagous genus Bursaphelenchus discovered inside figs in Japan. PLoS One 9:e99241. doi:10.1371/journal.pone.0099241 24940595 PMC4062417

[30] Kanzaki N, Tsai IJ, Tanaka R, Hunt VL, Liu D, Tsuyama K, Maeda Y, Namai S, Kumagai R, Tracey A, Holroyd N, Doyle SR, Woodruff GC, Murase K, Kitazume H, Chai C, Akagi A, Panda O, Ke H-M, Schroeder FC, Wang J, Berriman M, Sternberg PW, Sugimoto A, Kikuchi T. 2018. Biology and genome of a newly discovered sibling species of Caenorhabditis elegans. Nat Commun 9:3216. doi:10.1038/s41467-018-05712-5 30097582 PMC6086898

[31] Brenner S. 1974. The genetics of Caenorhabditis elegans. Genetics 77:71–94. doi:10.1093/genetics/77.1.71 4366476 PMC1213120

[32] Petersen C, Dirksen P, Schulenburg H. 2015. Why we need more ecology for genetic models such as C. elegans. Trends Genet 31:120–127. doi:10.1016/j.tig.2014.12.001 25577479

[33] Frézal L, Félix M-A. 2015. C. elegans outside the petri dish. eLife 4:e05849. doi:10.7554/eLife.05849 25822066 PMC4373675

[34] Kiontke KC, Félix M-A, Ailion M, Rockman MV, Braendle C, Pénigault J-B, Fitch DH. 2011. A phylogeny and molecular barcodes for Caenorhabditis, with numerous new species from rotting fruits. BMC Evol Biol 11:339. doi:10.1186/1471-2148-11-339 22103856 PMC3277298

[35] Berg M, Stenuit B, Ho J, Wang A, Parke C, Knight M, Alvarez-Cohen L, Shapira M. 2016. Assembly of the Caenorhabditis elegans gut microbiota from diverse soil microbial environments. ISME J 10:1998–2009. doi:10.1038/ismej.2015.253 26800234 PMC5029150

[36] Dirksen Philipp, Marsh SA, Braker I, Heitland N, Wagner S, Nakad R, Mader S, Petersen C, Kowallik V, Rosenstiel P, Félix M-A, Schulenburg H. 2016. The native microbiome of the nematode Caenorhabditis elegans: gateway to a new host-microbiome model. BMC Biol 14:38. doi:10.1186/s12915-016-0258-1 27160191 PMC4860760

[37] Samuel BS, Rowedder H, Braendle C, Félix M-A, Ruvkun G. 2016. Caenorhabditis elegans responses to bacteria from its natural habitats. Proc Natl Acad Sci USA 113:E3941–E3949. doi:10.1073/pnas.1607183113 27317746 PMC4941482

[38] Zhang F, Berg M, Dierking K, Félix M-A, Shapira M, Samuel BS, Schulenburg H. 2017. Caenorhabditis elegans as a model for microbiome research. Front Microbiol 8:485. doi:10.3389/fmicb.2017.00485 28386252 PMC5362939

[39] Singh A, Luallen RJ. 2024. Understanding the factors regulating host-microbiome interactions using Caenorhabditis elegans. Philos Trans R Soc Lond B Biol Sci 379:20230059. doi:10.1098/rstb.2023.0059 38497260 PMC10945399

[40] Rausch P, Rühlemann M, Hermes BM, Doms S, Dagan T, Dierking K, Domin H, Fraune S, von Frieling J, Hentschel U, et al.. 2019. Comparative analysis of amplicon and metagenomic sequencing methods reveals key features in the evolution of animal metaorganisms. Microbiome 7:133. doi:10.1186/s40168-019-0743-1 31521200 PMC6744666

[41] Schulenburg H, Félix M-A. 2017. The natural biotic environment of Caenorhabditis elegans. Genetics 206:55–86. doi:10.1534/genetics.116.195511 28476862 PMC5419493

[42] Thakur MP, Geisen S. 2019. Trophic regulations of the soil microbiome. Trends Microbiol 27:771–780. doi:10.1016/j.tim.2019.04.008 31138481

[43] Lewis Z, Lizé A. 2015. Insect behaviour and the microbiome. Curr Opin Insect Sci 9:86–90. doi:10.1016/j.cois.2015.03.003 32846714

[44] Trivedi P, Leach JE, Tringe SG, Sa T, Singh BK. 2020. Plant–microbiome interactions: from community assembly to plant health. Nat Rev Microbiol 18:607–621. doi:10.1038/s41579-020-0412-1 32788714

[45] Herre EA, Jandér KC, Machado CA. 2008. Evolutionary ecology of figs and their associates: recent progress and outstanding puzzles. Annu Rev Ecol Evol Syst 39:439–458. doi:10.1146/annurev.ecolsys.37.091305.110232

[46] Neu AT, Allen EE, Roy K. 2021. Defining and quantifying the core microbiome: challenges and prospects. Proc Natl Acad Sci USA 118. doi:10.1073/pnas.2104429118 PMC8713806 34862327

[47] Green PN, Ardley JK. 2018. Review of the genus Methylobacterium and closely related organisms: a proposal that some Methylobacterium species be reclassified into a new genus, Methylorubrum gen. nov. Int J Syst Evol Microbiol 68:2727–2748. doi:10.1099/ijsem.0.002856 30024371

[48] Flores-Félix JD, Menéndez E, Peix A, García-Fraile P, Velázquez E. 2020. History and current taxonomic status of genus Agrobacterium. Syst Appl Microbiol 43:126046. doi:10.1016/j.syapm.2019.126046 31818496

[49] Yabuuchi E, Yano I, Oyaizu H, Hashimoto Y, Ezaki T, Yamamoto H. 1990. Proposals of Sphingomonas paucimobilis gen. nov. and comb. nov., Sphingomonas parapaucimobilis sp. nov., Sphingomonas yanoikuyae sp. nov., Sphingomonas adhaesiva sp. nov., Sphingomonas capsulata comb. nov., and two genospecies of the genus Sphingomonas. Microbiol Immunol 34:99–119. doi:10.1111/j.1348-0421.1990.tb00996.x 2111872

[50] Müller DB, Vogel C, Bai Y, Vorholt JA. 2016. The plant microbiota: systems-level insights and perspectives. Annu Rev Genet 50:211–234. doi:10.1146/annurev-genet-120215-034952 27648643

[51] Yilmaz P, Parfrey LW, Yarza P, Gerken J, Pruesse E, Quast C, Schweer T, Peplies J, Ludwig W, Glöckner FO. 2014. The SILVA and “all-species living tree project (LTP)” taxonomic frameworks. Nucleic Acids Res 42:D643–D648. doi:10.1093/nar/gkt1209 24293649 PMC3965112

[52] Louca S, Parfrey LW, Doebeli M. 2016. Decoupling function and taxonomy in the global ocean microbiome. Science 353:1272–1277. doi:10.1126/science.aaf4507 27634532

[53] Donhauser J, Doménech-Pascual A, Han X, Jordaan K, Ramond J-B, Frossard A, Romaní AM, Priemé A. 2024. Modelling soil prokaryotic traits across environments with the trait sequence database amplicontraits and the R package MicEnvMod. Ecol Inform 83:102817. doi:10.1016/j.ecoinf.2024.102817

[54] Ning D, Yuan M, Wu L, Zhang Y, Guo X, Zhou X, Yang Y, Arkin AP, Firestone MK, Zhou J. 2020. A quantitative framework reveals ecological drivers of grassland microbial community assembly in response to warming. Nat Commun 11:4717. doi:10.1038/s41467-020-18560-z 32948774 PMC7501310

[55] Lin H, Peddada SD. 2020. Analysis of compositions of microbiomes with bias correction. Nat Commun 11:3514. doi:10.1038/s41467-020-17041-7 32665548 PMC7360769

[56] Herre EA. 1985. Sex ratio adjustment in fig wasps. Science 228:896–898. doi:10.1126/science.228.4701.896 17815055

[57] Herre EA. 1987. Optimality, plasticity and selective regime in fig wasp sex ratios. Nature 329:627–629. doi:10.1038/329627a0

[58] Molbo D, Machado CA, Herre EA, Keller L. 2004. Inbreeding and population structure in two pairs of cryptic fig wasp species. Mol Ecol 13:1613–1623. doi:10.1111/j.1365-294X.2004.02158.x 15140104

[59] Hamilton WD. 1967. Extraordinary sex ratios. Science 156:477–488. doi:10.1126/science.156.3774.477 6021675

[60] Shi R, Miao B, Segar ST, Zeng Y, Wang B, Peng Y. 2019. Are nematodes costly to fig tree–fig wasp mutualists? Entomologia Exp Applicata 167:1000–1011. doi:10.1111/eea.12860

[61] Holland MA, Polacco JC. 1994. PPFMs and other covert contaminants: is there more to plant physiology than just plant? Annu Rev Plant Physiol Plant Mol Biol 45:197–209. doi:10.1146/annurev.pp.45.060194.001213

[62] Madhaiyan M, Poonguzhali S, Lee HS, Hari K, Sundaram SP, Sa TM. 2005. Pink-pigmented facultative methylotrophic bacteria accelerate germination, growth and yield of sugarcane clone Co86032 (Saccharum officinarum L.). Biol Fertil Soils 41:350–358. doi:10.1007/s00374-005-0838-7

[63] Asaf S, Numan M, Khan AL, Al-Harrasi A. 2020. Sphingomonas: from diversity and genomics to functional role in environmental remediation and plant growth. Crit Rev Biotechnol 40:138–152. doi:10.1080/07388551.2019.1709793 31906737

[64] Khan AL, Waqas M, Kang S-M, Al-Harrasi A, Hussain J, Al-Rawahi A, Al-Khiziri S, Ullah I, Ali L, Jung H-Y, Lee I-J. 2014. Bacterial endophyte Sphingomonas sp. LK11 produces gibberellins and IAA and promotes tomato plant growth. J Microbiol 52:689–695. doi:10.1007/s12275-014-4002-7 24994010

[65] Luo Y, Wang F, Huang Y, Zhou M, Gao J, Yan T, Sheng H, An L. 2019. Sphingomonas sp. Cra20 increases plant growth rate and alters rhizosphere microbial community structure of Arabidopsis thaliana under drought stress. Front Microbiol 10:1221. doi:10.3389/fmicb.2019.01221 31231328 PMC6560172

[66] Lewin GR, Carlos C, Chevrette MG, Horn HA, McDonald BR, Stankey RJ, Fox BG, Currie CR. 2016. Evolution and ecology of Actinobacteria and their bioenergy applications. Annu Rev Microbiol 70:235–254. doi:10.1146/annurev-micro-102215-095748 27607553 PMC5703056

[67] Duangmal K, Thamchaipenet A, Ara I, Matsumoto A, Takahashi Y. 2008. Kineococcus gynurae sp. nov., isolated from a Thai medicinal plant. Int J Syst Evol Microbiol 58:2439–2442. doi:10.1099/ijs.0.65671-0 18842871

[68] Mhatre S, Singh NK, Wood JM, Parker CW, Pukall R, Verbarg S, Tindall BJ, Neumann-Schaal M, Venkateswaran K. 2020. Description of chloramphenicol resistant Kineococcus rubinsiae sp. nov. isolated from a spacecraft assembly facility. Front Microbiol 11. doi:10.3389/fmicb.2020.01957 PMC7472656 32973710

[69] Doughari HJ, Ndakidemi PA, Human IS, Benade S. 2011. The ecology, biology and pathogenesis of Acinetobacter spp.: an overview. Microbes Environ 26:101–112. doi:10.1264/jsme2.me10179 21502736

[70] Jung J, Park W. 2015. Acinetobacter species as model microorganisms in environmental microbiology: current state and perspectives. Appl Microbiol Biotechnol 99:2533–2548. doi:10.1007/s00253-015-6439-y 25693672

[71] Finster KW, Herbert RA, Lomstein BA. 2009. Spirosoma spitsbergense sp. nov. and Spirosoma luteum sp. nov., isolated from a high Arctic permafrost soil, and emended description of the genus Spirosoma. Int J Syst Evol Microbiol 59:839–844. doi:10.1099/ijs.0.002725-0 19329617

[72] Jung Y, Chhetri G, Kim I, So Y, Park S, Woo H, Lee K-H, Seo T. 2023. Chryseobacterium edaphi sp. nov. and Chryseobacterium gilvum sp. nov., isolated from soil. Int J Syst Evol Microbiol 73:005989. doi:10.1099/ijsem.0.005989 37490399

[73] Liu Y, Fan S, Yu H. 2021. Analysis of Ficus hirta fig endosymbionts diversity and species composition. Diversity (Basel) 13:636. doi:10.3390/d13120636

[74] Jauharlina J, et al.. 2022. Association of fig pollinating wasps and fig nematodes inside male and female figs of a dioecious fig tree in Sumatra, Indonesia. Insects 13:320. doi:10.3390/insects13040320 35447762 PMC9030183

[75] Berg M, Zhou XY, Shapira M. 2016. Host-specific functional significance of Caenorhabditis gut commensals. Front Microbiol 7:1622. doi:10.3389/fmicb.2016.01622 27799924 PMC5066524

[76] Corsi AK, Wightman B, Chalfie M. 2015. A transparent window into biology: a primer on Caenorhabditis elegans. Genetics 200:387–407. doi:10.1534/genetics.115.176099 26088431 PMC4492366

[77] Kvon EZ, Kamneva OK, Melo US, Barozzi I, Osterwalder M, Mannion BJ, Tissières V, Pickle CS, Plajzer-Frick I, Lee EA, Kato M, Garvin TH, Akiyama JA, Afzal V, Lopez-Rios J, Rubin EM, Dickel DE, Pennacchio LA, Visel A. 2016. Progressive loss of function in a limb enhancer during snake evolution. Cell 167:633–642. doi:10.1016/j.cell.2016.09.028 27768887 PMC5484524

[78] Lynch M. 2007. The origins of genome architecture. mitpressbookstore. Sinaur. https://mitpressbookstore.mit.edu/book/9780878934843.

[79] Senevirathne G, Fernandopulle SC, Richard D, Baumgart SL, Christensen AL, Fabbri M, Höppner J, Jüppner H, Li P, Bothe V, Fröbisch N, Simcock I, Arthurs OJ, Calder A, Freilich N, Nowlan NC, Glass IA, Craft A, Capellini TD. 2025. The evolution of hominin bipedalism in two steps. Nature 645:952–963. doi:10.1038/s41586-025-09399-9 40866708 PMC12460174

[80] Spehr M, Munger SD. 2009. Olfactory receptors: G protein-coupled receptors and beyond. J Neurochem 109:1570–1583. doi:10.1111/j.1471-4159.2009.06085.x 19383089 PMC4455932

[81] Ma F, Lau CY, Zheng C. 2021. Large genetic diversity and strong positive selection in F-box and GPCR genes among the wild isolates of Caenorhabditis elegans. Genome Biol Evol 13:evab048. doi:10.1093/gbe/evab048 33693740 PMC8120010

[82] Link AC, Moser KA, Wang J, Woodruff GC. n.d. A novel, fig-associated microbe promotes reproductive success via variable life history mechanisms in C. elegans and C. inopinata. bioRxiv. doi:10.64898/2025.12.16.694684

[83] Hall AB, Tolonen AC, Xavier RJ. 2017. Human genetic variation and the gut microbiome in disease. Nat Rev Genet 18:690–699. doi:10.1038/nrg.2017.63 28824167

[84] Ørsted M, Yashiro E, Hoffmann AA, Kristensen TN. 2022 Population bottlenecks constrain host microbiome diversity and genetic variation impeding fitness. PLoS Genet 18:e1010206. doi:10.1371/journal.pgen.1010206 35604942 PMC9166449

[85] Zhang F, Weckhorst JL, Assié A, Hosea C, Ayoub CA, Khodakova AS, Cabrera ML, Vidal Vilchis D, Félix M-A, Samuel BS. 2021. Natural genetic variation drives microbiome selection in the Caenorhabditis elegans gut. Curr Biol 31:2603–2618. doi:10.1016/j.cub.2021.04.046 34048707 PMC8222194

[86] Johnke J, Dirksen P, Schulenburg H. 2020. Community assembly of the native C. elegans microbiome is influenced by time, substrate and individual bacterial taxa. Environ Microbiol 22:1265–1279. doi:10.1111/1462-2920.14932 32003074

[87] Leech T, McDowall L, Hopkins KP, Sait SM, Harrison XA, Bretman A. 2021. Social environment drives sex and age-specific variation in Drosophila melanogaster microbiome composition and predicted function. Mol Ecol 30:5831–5843. doi:10.1111/mec.16149 34494339

[88] Parizadeh M, Arrieta M-C. 2023. The global human gut microbiome: genes, lifestyles, and diet. Trends Mol Med 29:789–801. doi:10.1016/j.molmed.2023.07.002 37516570

[89] Lin R-C, Yeung CK-L, Li S-H. 2008. Drastic post-LGM expansion and lack of historical genetic structure of a subtropical fig-pollinating wasp (Ceratosolen sp. 1) of Ficus septica in Taiwan. Mol Ecol 17:5008–5022. doi:10.1111/j.1365-294X.2008.03983.x 19120988

[90] Rodriguez LJ, Bain A, Chou L-S, Conchou L, Cruaud A, Gonzales R, Hossaert-McKey M, Rasplus J-Y, Tzeng H-Y, Kjellberg F. 2017. Diversification and spatial structuring in the mutualism between Ficus septica and its pollinating wasps in insular South East Asia. BMC Evol Biol 17:207. doi:10.1186/s12862-017-1034-8 28851272 PMC5576367

[91] Bain A, Chou L-S, Tzeng H-Y, Ho Y-C, Chiang Y-P, Chen W-H, Chio Y-T, Li G-Y, Yang H-W, Kjellberg F, Hossaert-McKey M. 2014. Plasticity and diversity of the phenology of dioecious Ficus species in Taiwan. Acta Oecologica 57:124–134. doi:10.1016/j.actao.2013.10.004

[92] Smith B, Wilson JB. 1996. A consumer’s guide to evenness indices. Oikos 76:70. doi:10.2307/3545749

[93] Venturi V, Fuqua C. 2013. Chemical signaling between plants and plant-pathogenic bacteria. Annu Rev Phytopathol 51:17–37. doi:10.1146/annurev-phyto-082712-102239 23915131

[94] Zeilinger S, Gupta VK, Dahms TES, Silva RN, Singh HB, Upadhyay RS, Gomes EV, Tsui CK-M, Nayak S C. 2016. Friends or foes? emerging insights from fungal interactions with plants. FEMS Microbiol Rev 40:182–207. doi:10.1093/femsre/fuv045 26591004 PMC4778271

[95] Deveau A, Bonito G, Uehling J, Paoletti M, Becker M, Bindschedler S, Hacquard S, Hervé V, Labbé J, Lastovetsky OA, Mieszkin S, Millet LJ, Vajna B, Junier P, Bonfante P, Krom BP, Olsson S, van Elsas JD, Wick LY. 2018. Bacterial-fungal interactions: ecology, mechanisms and challenges. FEMS Microbiol Rev 42:335–352. doi:10.1093/femsre/fuy008 29471481

[96] Kanzaki N, Tanaka R. 2025. Aphelenchoides epiphyticus n. sp. (Tylenchomorpha: Aphelenchoididae) isolated from figs of Ficus septica in Japan. Nematol 27:697–715. doi:10.1163/15685411-bja10416

[97] Sloat SA, Noble LM, Paaby AB, Bernstein M, Chang A, Kaur T, Yuen J, Tintori SC, Jackson JL, Martel A, Salome Correa JA, Stevens L, Kiontke K, Blaxter M, Rockman MV. 2022. Caenorhabditis nematodes colonize ephemeral resource patches in neotropical forests. Ecol Evol 12:e9124. doi:10.1002/ece3.9124 35898425 PMC9309040

[98] Woodruff GC, Willis JH, Phillips PC. 2018. Dramatic evolution of body length due to postembryonic changes in cell size in a newly discovered close relative of Caenorhabditis elegans Evol Lett 2:427–441. doi:10.1002/evl3.67 30283693 PMC6121821

[99] Kiontke K, Gavin NP, Raynes Y, Roehrig C, Piano F, Fitch DHA. 2004. Caenorhabditis phylogeny predicts convergence of hermaphroditism and extensive intron loss. Proc Natl Acad Sci USA 101:9003–9008. doi:10.1073/pnas.0403094101 15184656 PMC428462

[100] Woodruff GC, Johnson E, Phillips PC. 2019. A large close relative of C. elegans is slow-developing but not long-lived. BMC Evol Biol 19:74. doi:10.1186/s12862-019-1388-1 30866802 PMC6416856

[101] Dirksen P. 2020. CeMbio - the Caenorhabditis elegans microbiome resource. G3 (Bethesda) 10:3025–3039. doi:10.1534/g3.120.401309 32669368 PMC7466993

[102] Zimmermann J, Piecyk A, Sieber M, Petersen C, Johnke J, Moitinho-Silva L, Künzel S, Bluhm L, Traulsen A, Kaleta C, Schulenburg H. 2024. Gut-associated functions are favored during microbiome assembly across a major part of C. elegans life. mBio 15:e0001224. doi:10.1128/mbio.00012-24 38634692 PMC11077962

[103] Michelou VK, Caporaso JG, Knight R, Palumbi SR. 2013. The ecology of microbial communities associated with Macrocystis pyrifera. PLoS One 8:e67480. doi:10.1371/journal.pone.0067480 23840715 PMC3686729

[104] Lundberg DS, Yourstone S, Mieczkowski P, Jones CD, Dangl JL. 2013. Practical innovations for high-throughput amplicon sequencing. Nat Methods 10:999–1002. doi:10.1038/nmeth.2634 23995388

[105] Galil J, Eisikowitch D. 1968. Flowering cycles and fruit types of Ficus sycomorus in Israel. New Phytologist 67:745–758. doi:10.1111/j.1469-8137.1968.tb05497.x

[106] Caporaso JG, Lauber CL, Walters WA, Berg-Lyons D, Lozupone CA, Turnbaugh PJ, Fierer N, Knight R. 2011. Global patterns of 16S rRNA diversity at a depth of millions of sequences per sample. Proc Natl Acad Sci USA 108:4516–4522. doi:10.1073/pnas.1000080107 20534432 PMC3063599

[107] Andrews S. 2010. Babraham Bioinformatics. Available from: https://www.bioinformatics.babraham.ac.uk/projects/fastqc/

[108] Callahan BJ, McMurdie PJ, Rosen MJ, Han AW, Johnson AJA, Holmes SP. 2016. DADA2: High-resolution sample inference from Illumina amplicon data. Nat Methods 13:581–583. doi:10.1038/nmeth.3869 27214047 PMC4927377

[109] Bolyen E, Rideout JR, Dillon MR, Bokulich NA, Abnet CC, Al-Ghalith GA, Alexander H, Alm EJ, Arumugam M, Asnicar F, et al.. 2019. Reproducible, interactive, scalable and extensible microbiome data science using QIIME 2. Nat Biotechnol 37:852–857. doi:10.1038/s41587-019-0209-9 31341288 PMC7015180

[110] Ii MSR, et al.. 2021. RESCRIPt: reproducible sequence taxonomy reference database management. PLoS Comput Biol 17:11. doi:10.1371/journal.pcbi.1009581 PMC8601625 34748542

[111] Quast C, Pruesse E, Yilmaz P, Gerken J, Schweer T, Yarza P, Peplies J, Glöckner FO. 2013. The SILVA ribosomal RNA gene database project: improved data processing and web-based tools. Nucleic Acids Res 41:D590–D596. doi:10.1093/nar/gks1219 23193283 PMC3531112

[112] Rognes T, Flouri T, Nichols B, Quince C, Mahé F. 2016. VSEARCH: a versatile open source tool for metagenomics. PeerJ 4:e2584. doi:10.7717/peerj.2584 27781170 PMC5075697

[113] Katoh K, Misawa K, Kuma K, Miyata T. 2002. MAFFT: a novel method for rapid multiple sequence alignment based on fast Fourier transform. Nucleic Acids Res 30:3059–3066. doi:10.1093/nar/gkf436 12136088 PMC135756

[114] Price MN, Dehal PS, Arkin AP. 2009. FastTree: computing large minimum evolution trees with profiles instead of a distance matrix. Mol Biol Evol 26:1641–1650. doi:10.1093/molbev/msp077 19377059 PMC2693737

[115] R Core Team. 2025 R foundation for statistical computing. Available from: https://www.R-project.org/

[116] McMurdie PJ, Holmes S. 2013. Phyloseq: an R package for reproducible interactive analysis and graphics of microbiome census data. PLoS One 8:e61217. doi:10.1371/journal.pone.0061217 23630581 PMC3632530

[117] Sudarshan SA. 2018. Microbial bioinformatics introductory course material 2018. Available from: 10.5281/zenodo.1436630

[118] Quinn TP, Erb I, Richardson MF, Crowley TM. 2018. Understanding sequencing data as compositions: an outlook and review. Bioinformatics 34:2870–2878. doi:10.1093/bioinformatics/bty175 29608657 PMC6084572

[119] Liu C, Cui Y, Li X, Yao M. 2021. Microeco: an R package for data mining in microbial community ecology. FEMS Microbiol Ecol 97:fiaa255. doi:10.1093/femsec/fiaa255 33332530

[120] Liu Chi, Li C, Jiang Y, Zeng RJ, Yao M, Li X. 2023. A guide for comparing microbial co‐occurrence networks. iMeta 2:e71. doi:10.1002/imt2.71 38868345 PMC10989802

[121] Madin JS, Nielsen DA, Brbic M, Corkrey R, Danko D, Edwards K, Engqvist MKM, Fierer N, Geoghegan JL, Gillings M, et al.. 2020. A synthesis of bacterial and archaeal phenotypic trait data. Sci Data 7:170. doi:10.1038/s41597-020-0497-4 32503990 PMC7275036

[122] Paradis E, Schliep K. 2019. Ape 5.0: an environment for modern phylogenetics and evolutionary analyses in R. Bioinformatics 35:526–528. doi:10.1093/bioinformatics/bty633 30016406

[123] Wilke C. 2020. Cowplot: streamlined plot theme and plot annotations for “ggplot2. Available from: https://CRAN.R-project.org/package=cowplot

[124] Barrett T, Dowle M, Srinivasan A, Gorecki J, Chirico M, Hocking T, Schwendinger B, Krylov I, Stetsenko P, Short T, et al.. 2024. Data.table: Extension of “data.frame”. Available from: https://CRAN.R-project.org/package=data.table

[125] Wickham H, François R, Henry L, Müller K, Vaughan D, Girlich M, Smith R, Pedersen T, Hester J, Bryan J, et al.. 2023. Dplyr: a grammar of data manipulation. Available from: https://CRAN.R-project.org/package=dplyr

[126] Xie Y, Cheng J, Tan X. 2024. DT: A Wrapper of the JavaScript Library “DataTables”. Available from: https://CRAN.R-project.org/package=DT

[127] Torchiano M. 2016. Effsize - a package for efficient effect size computation. Available from: 10.5281/ZENODO.1480624

[128] Pedersen T. 2022. Ggforce: accelerating “Ggplot2.” Available from: https://CRAN.R-project.org/package=ggforce

[129] Kahle D, Wickham H. 2013. Ggmap: spatial visualization with ggplot2. R J 5:144. doi:10.32614/RJ-2013-014

[130] Wickham H 2016. Ggplot2: elegant graphics for data analysis. Springer-Verlag.

[131] Kassambara A. 2023. Ggpubr: “ggplot2” Based Publication Ready Plots. Available from: https://CRAN.R-project.org/package=ggpubr

[132] Edwards S. 2020. Lemon: freshing up your “Ggplot2” plots. Available from: https://CRAN.R-project.org/package=lemon

[133] Lahti L, Shetty S. 2012. Introduction to the microbiome R package. Available from: https://microbiome.github.io/tutorials/

[134] Shetty S, Lahti L. 2024. Microbiomeutilities. Available from: https://github.com/microsud/microbiomeutilities

[135] Pedersen T. 2022. Patchwork: the composer of plots

[136] Kembel SW, Cowan PD, Helmus MR, Cornwell WK, Morlon H, Ackerly DD, Blomberg SP, Webb CO. 2010. Picante: R tools for integrating phylogenies and ecology. Bioinformatics 26:1463–1464. doi:10.1093/bioinformatics/btq166 20395285

[137] Neuwirth E. 2022. RColorBrewer: ColorBrewer Palettes. Available from: https://CRAN.R-project.org/package=RColorBrewer

[138] Wickham H. 2007. Reshaping data with the reshape package. J Stat Softw 21:1–20. doi:10.18637/jss.v021.i12

[139] Oksanen J, Simpson G, Blanchet F, Kindt R, Legendre P, Minchin P, O’Hara R, Solymos P, Stevens M, Szoecs E, et al.. 2024. Vegan: community ecology package. Available from: https://cran.r-project.org/web/packages/vegan/index.html

[140] Conway JR, Lex A, Gehlenborg N. 2017. UpSetR: an R package for the visualization of intersecting sets and their properties. Bioinformatics 33:2938–2940. doi:10.1093/bioinformatics/btx364 28645171 PMC5870712

