# Supplementary material for: The bacteria of a fig community
Source: Microbiol Spectr. 2026 Mar 30;14(5):e03013-24. doi: 10.1128/spectrum.03013-24 (PMC13141926; doi:10.1128/spectrum.03013-24)
Supplement: Supplemental figures — Figures S1 to S27. [file spectrum.03013-24-s0001.pdf]

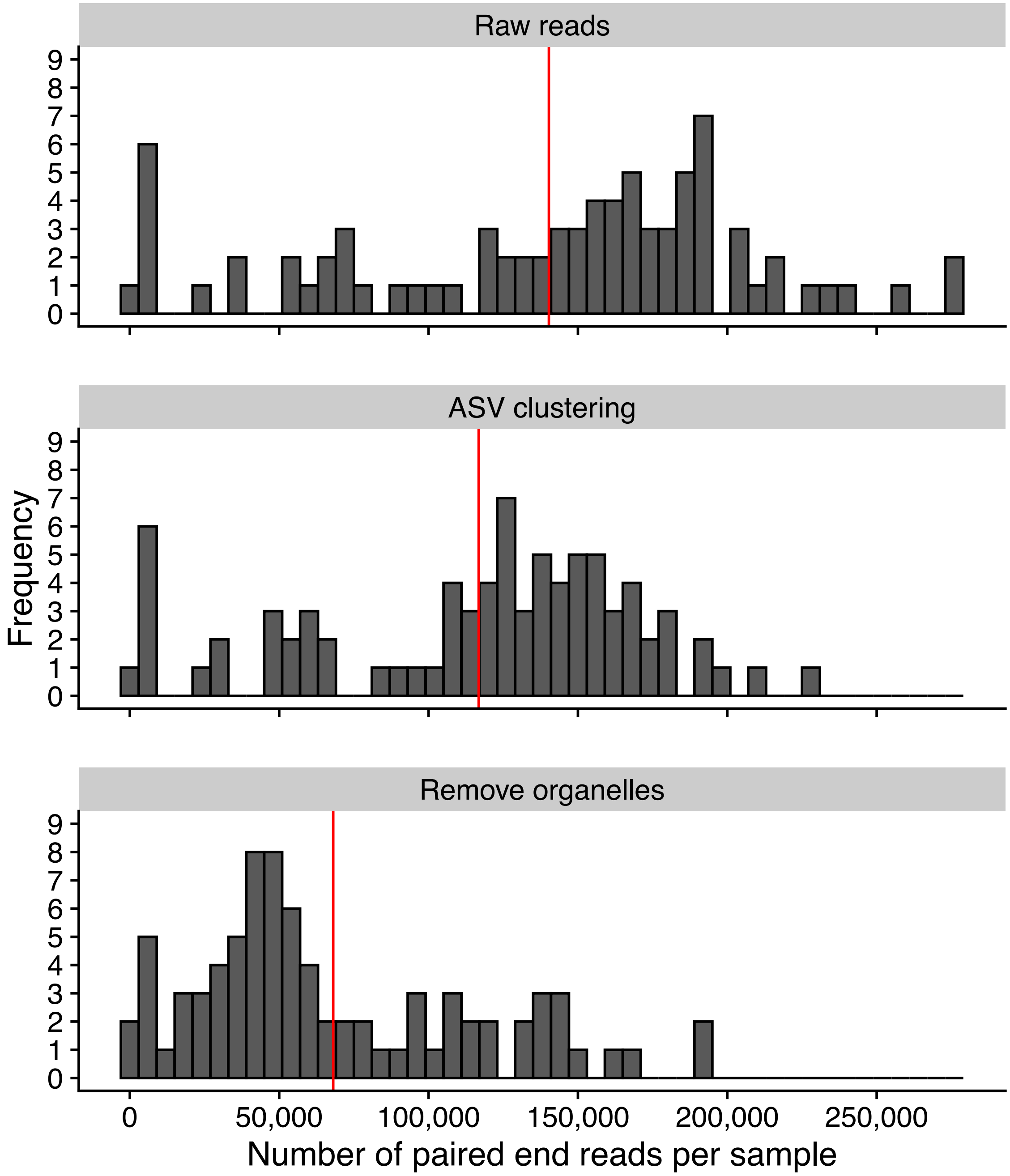

Supplemental Figure 1. Distribution of reads per fig sample. The vertical red line represents the mean in all panels. Top panel, distribution of raw read count per sample. Middle panel, distribution of read count per sample after ASV clustering (i.e., reads that correspond to ASVs after generating a feature table with QIIME2). Bottom panel, distribution of read count per sample after mitochondrion- and chloroplast-aligning reads were removed from the analysis.

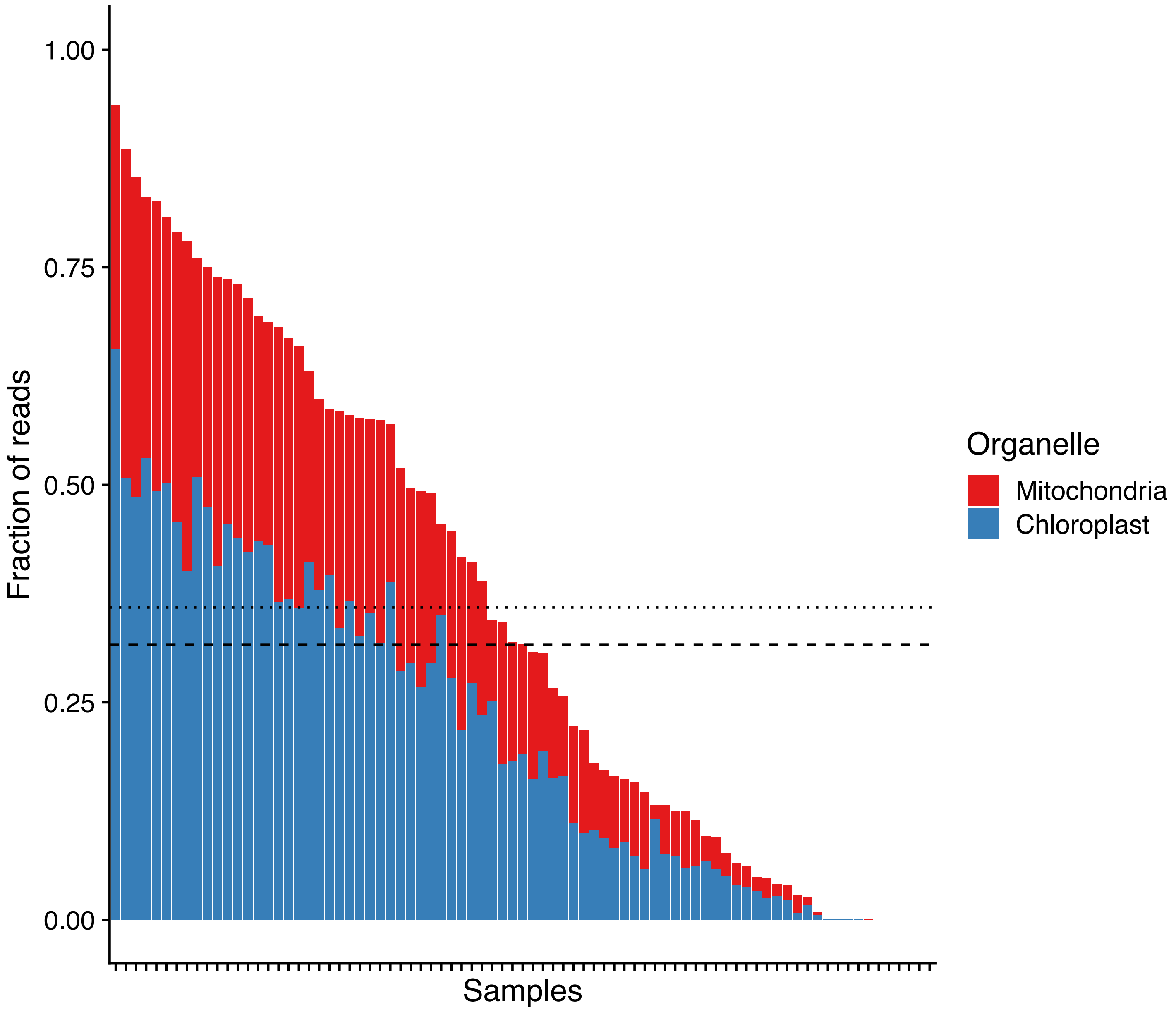

Supplemental Figure 2. Fraction of reads per fig sample aligning to organellar DNA. Dashed line, median fraction; Dotted line, mean fraction.

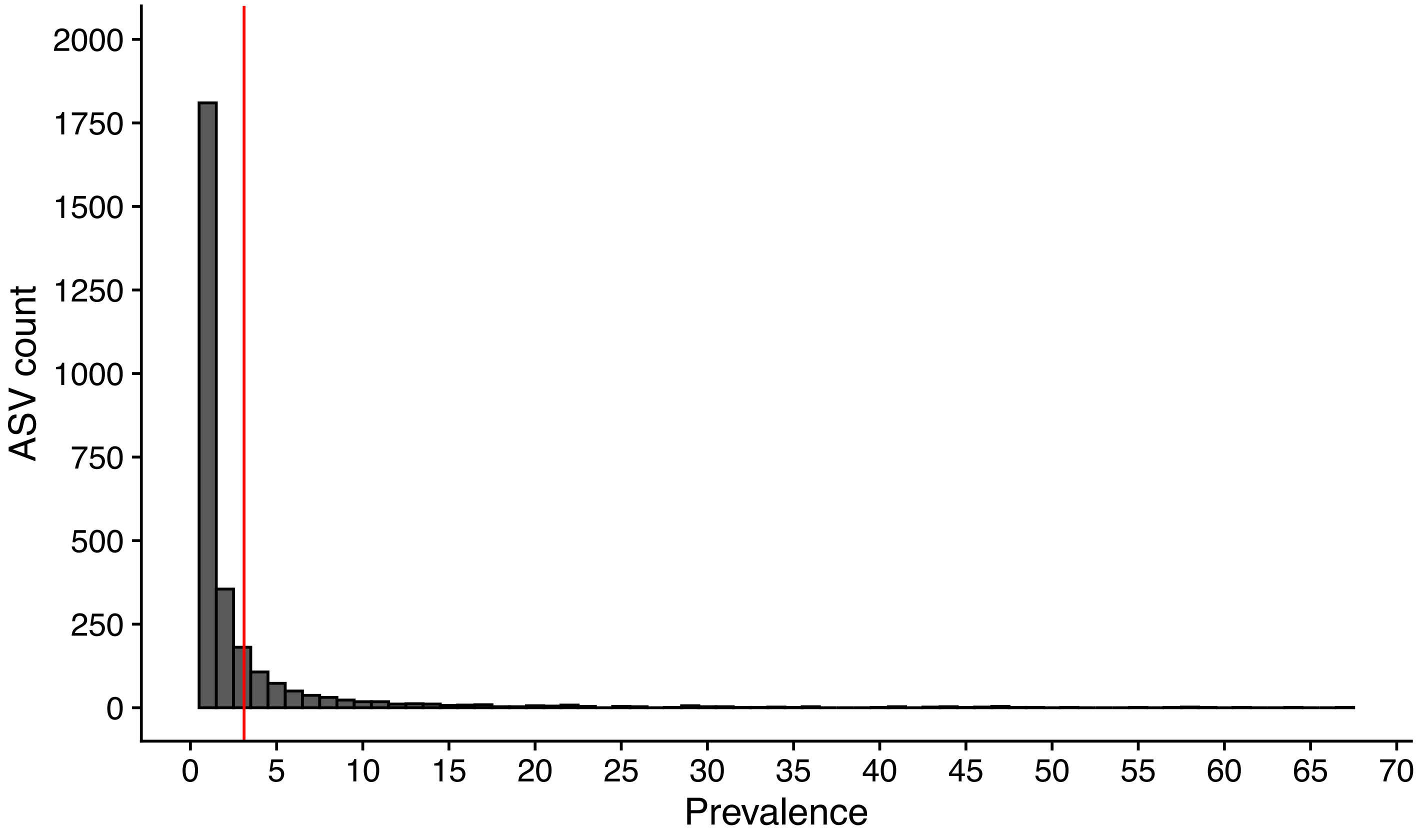

Supplemental Figure 3. Distribution of ASV prevalence in fig samples. The red vertical line represents the mean. No ASV was present in all fig samples.

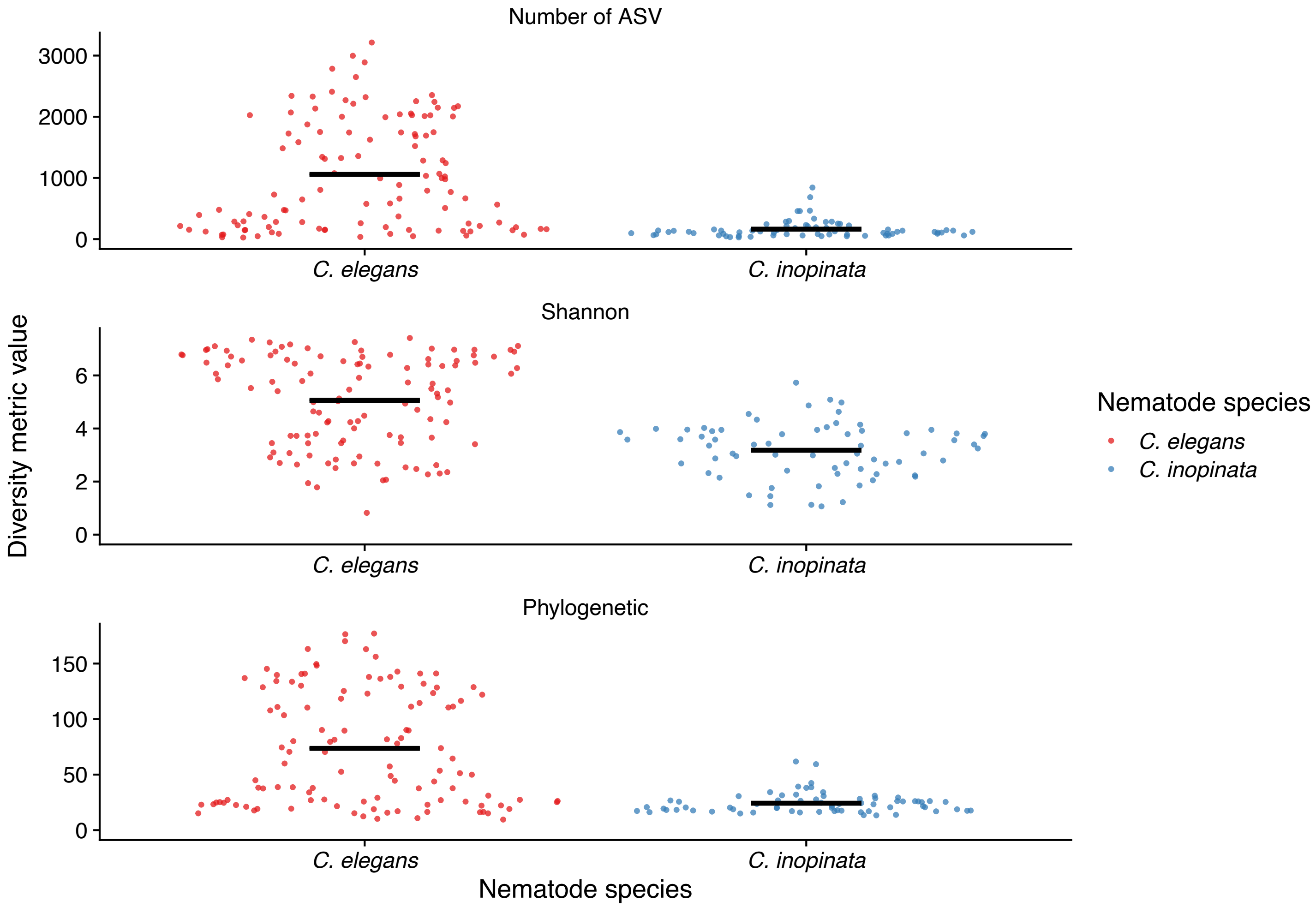

Supplemental Figure 4. α-diversity is higher in *C. elegans* substrates compared to *C. inopinata* substrates.

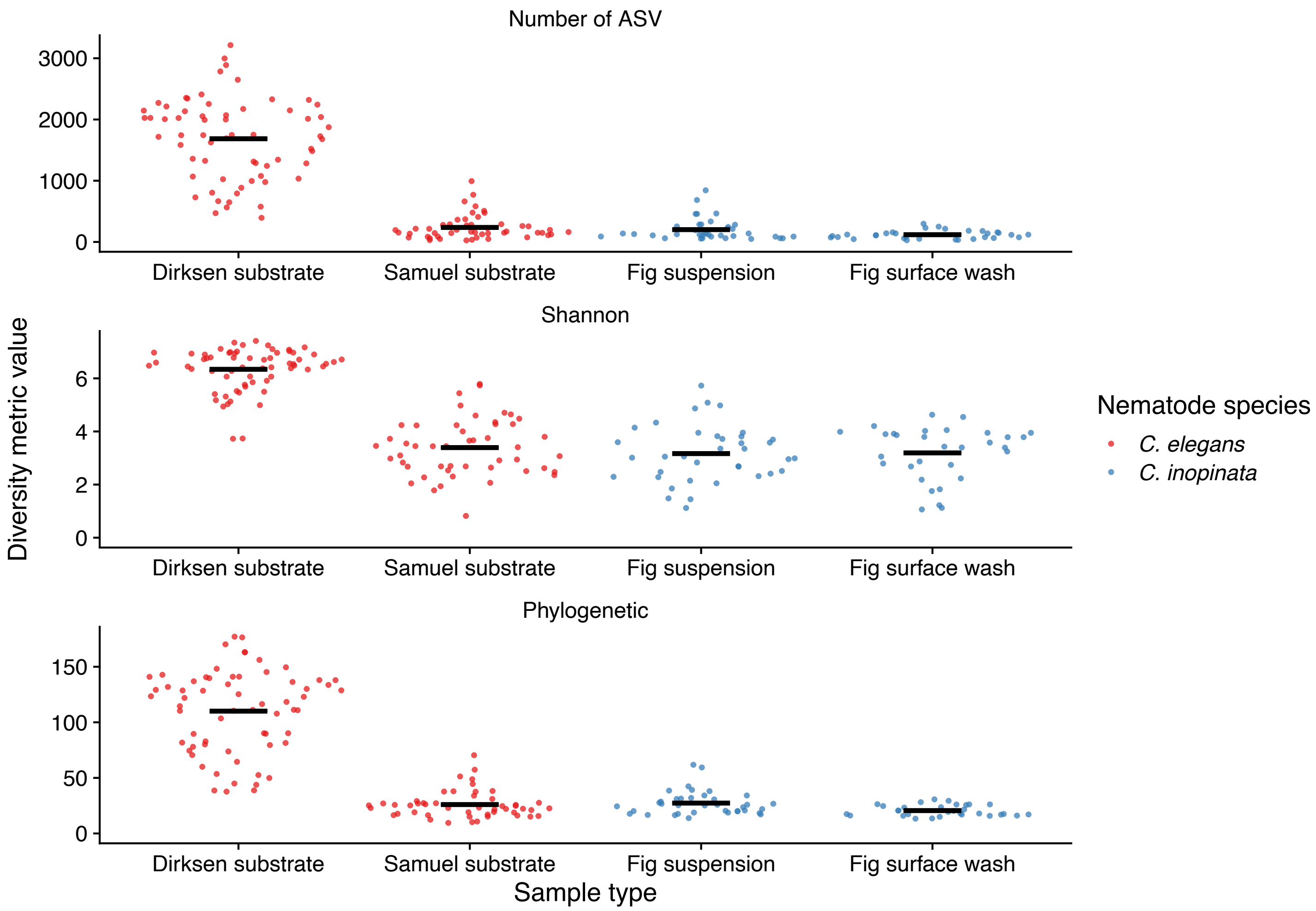

Supplemental Figure 5. α-diversity among four sample types. Across three measures, the compost samples described in Dirksen et al. 2016 harbor the most within-sample diversity.

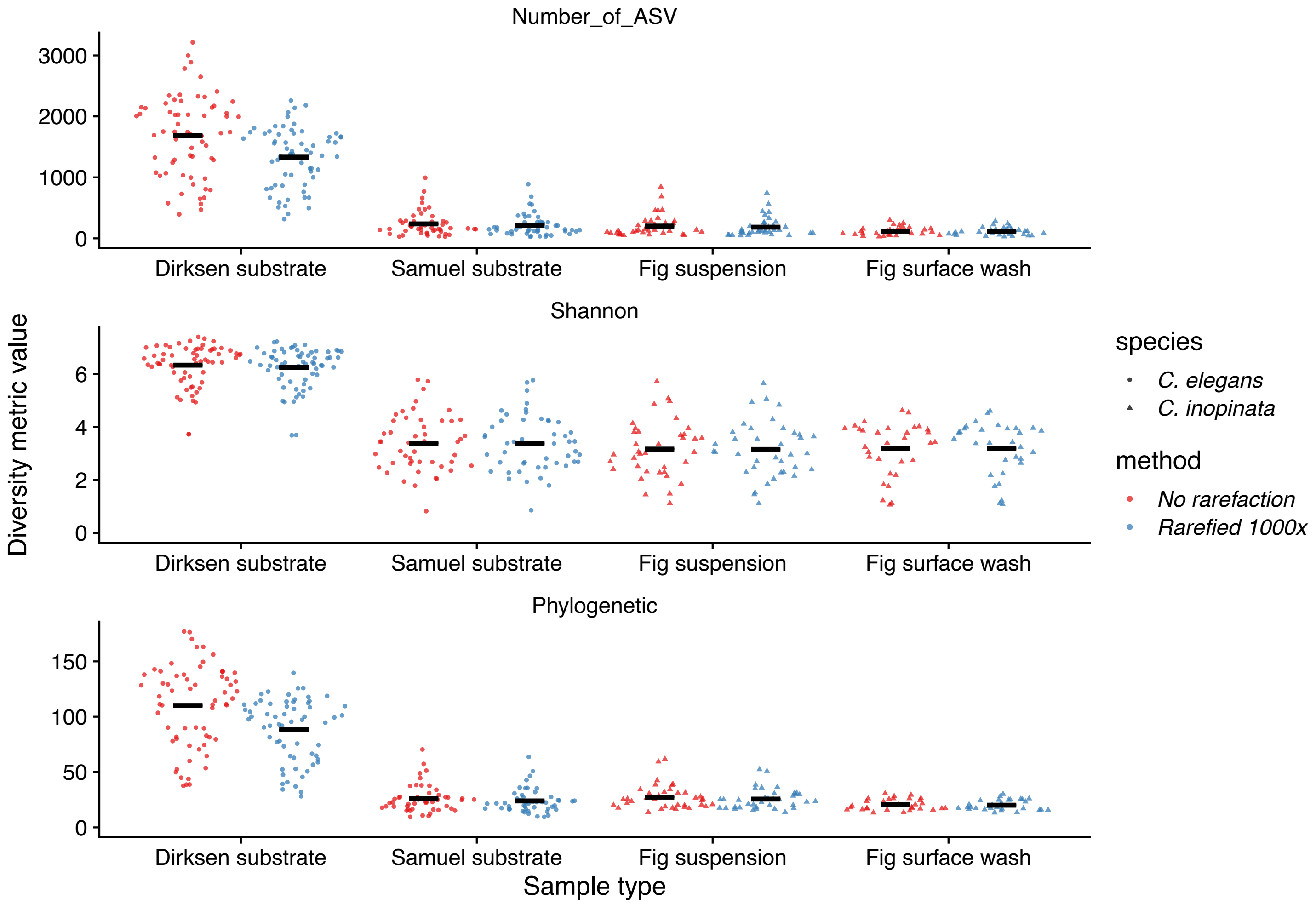

Supplemental Figure 6. Rarefaction impacts only high-diversity samples. For the "Rarefied 1000x" samples, each sample was rarefied one thousand times, and the mean diversity metric per sample is plotted.

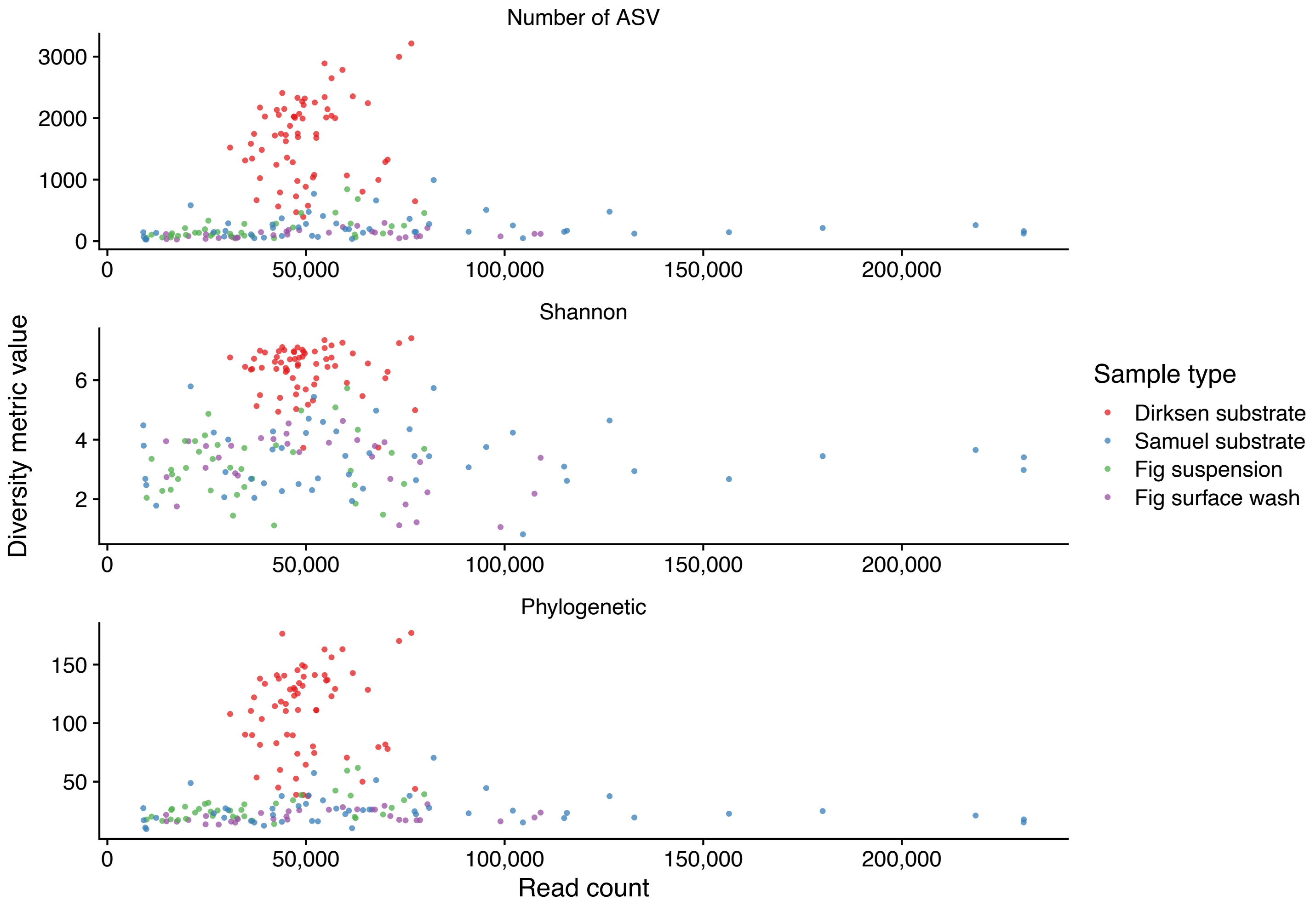

Supplemental Figure 7. There is no clear relationship between within-sample diversity and read count.

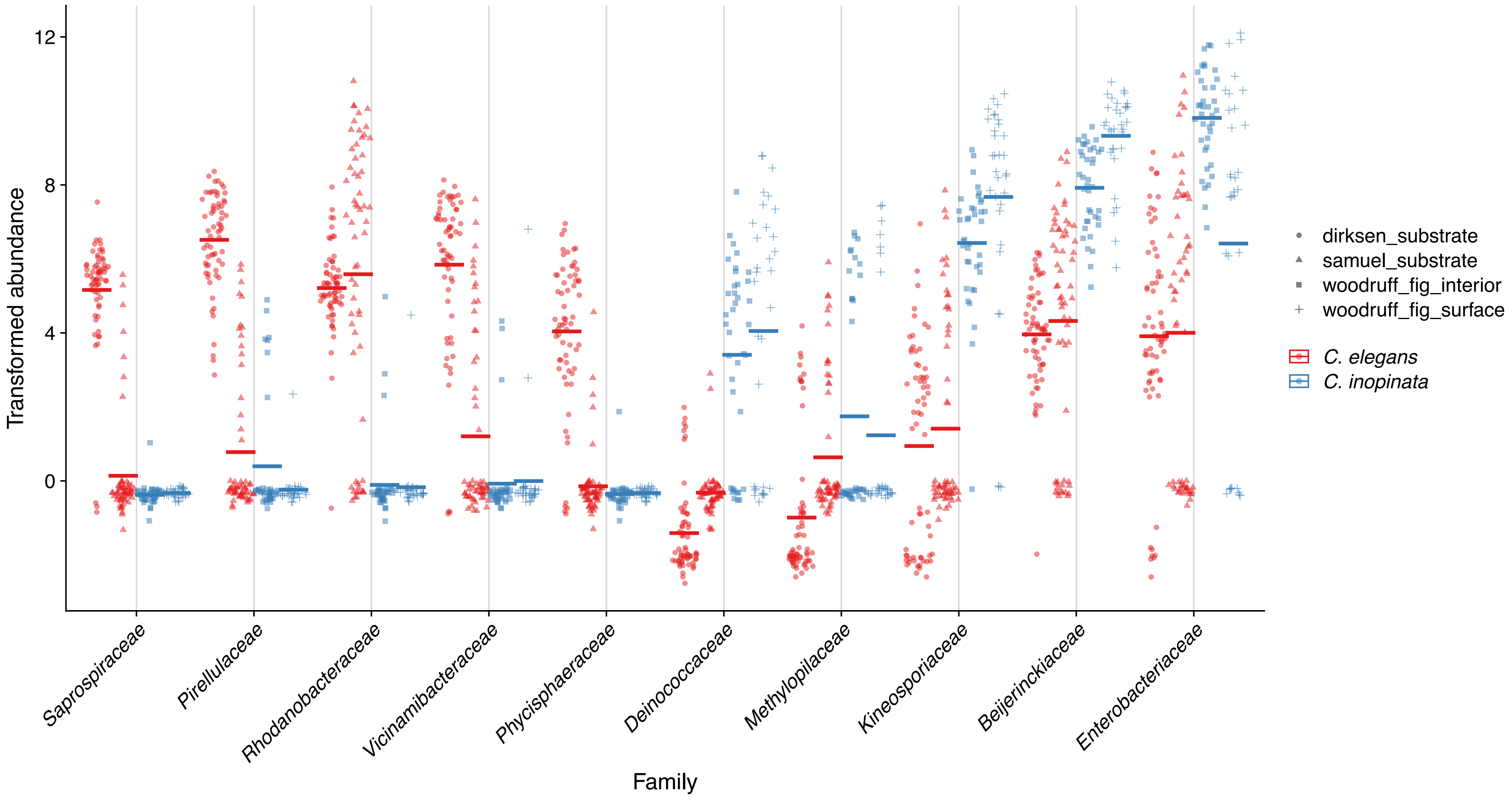

Supplemental Figure 8. Differentially abundant families among *C. elegans* and *C. inopinata* substrate samples.

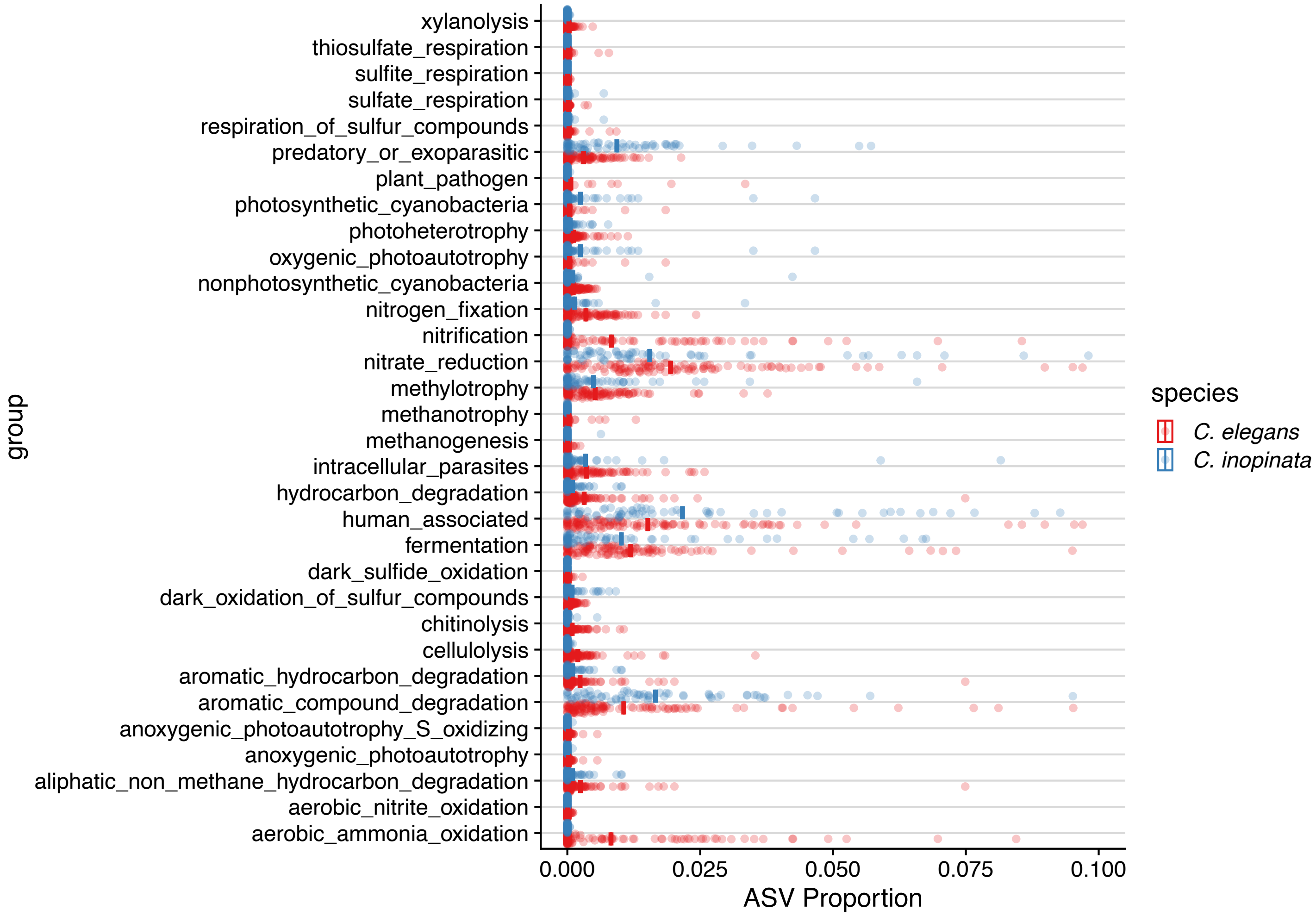

Supplemental Figure 9. FAPROTAX reveals functional differences among the microbial communities of *C. elegans* and *C. inopinata* substrates.

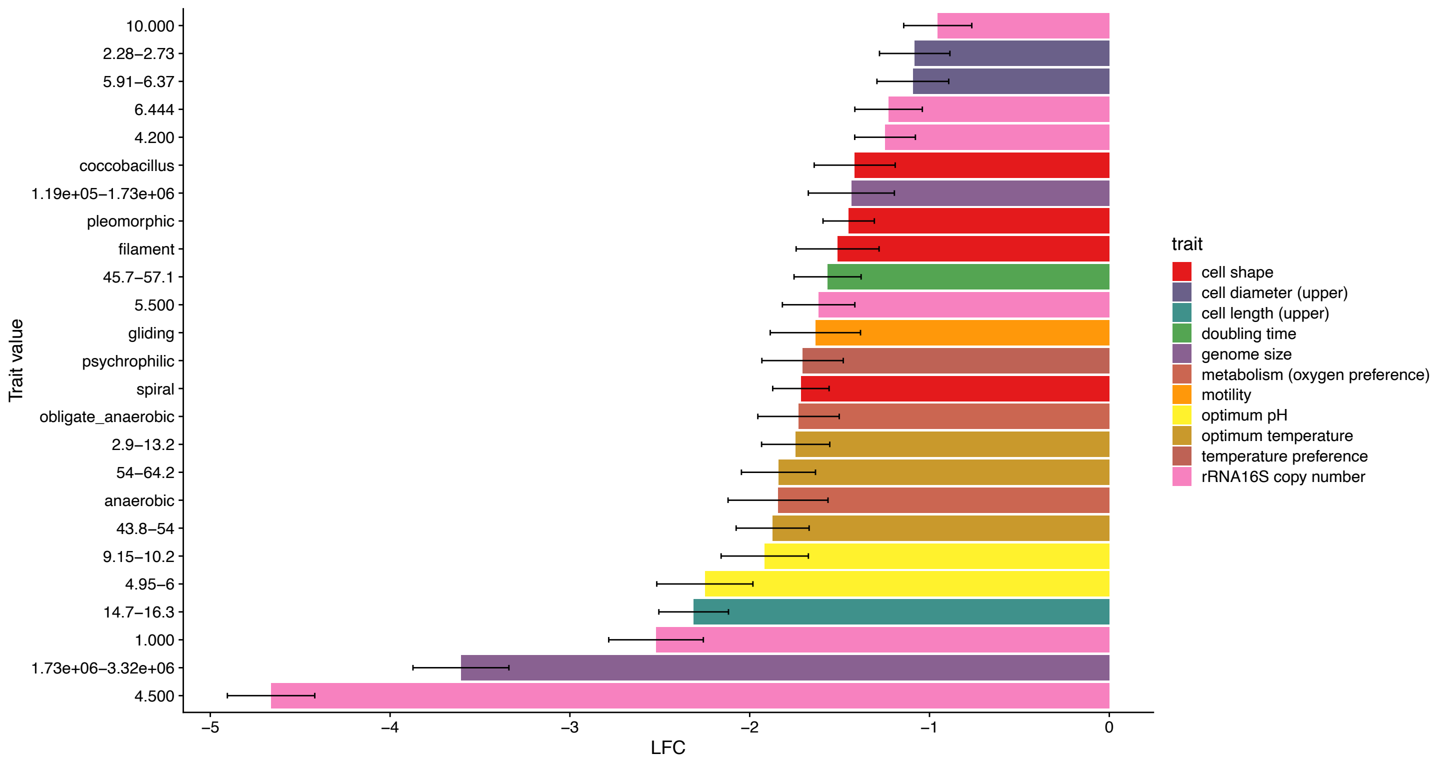

Supplemental Figure 10. *amplicontraits* reveals functional differences enriched in the microbial communities of *C. elegans* substrates (Log Fold Change (LFC) < 0). Details of traits, trait values, and their units can be found in the *amplicontraits* documentation (https://github.com/jdonhauser/ampliconTraits).

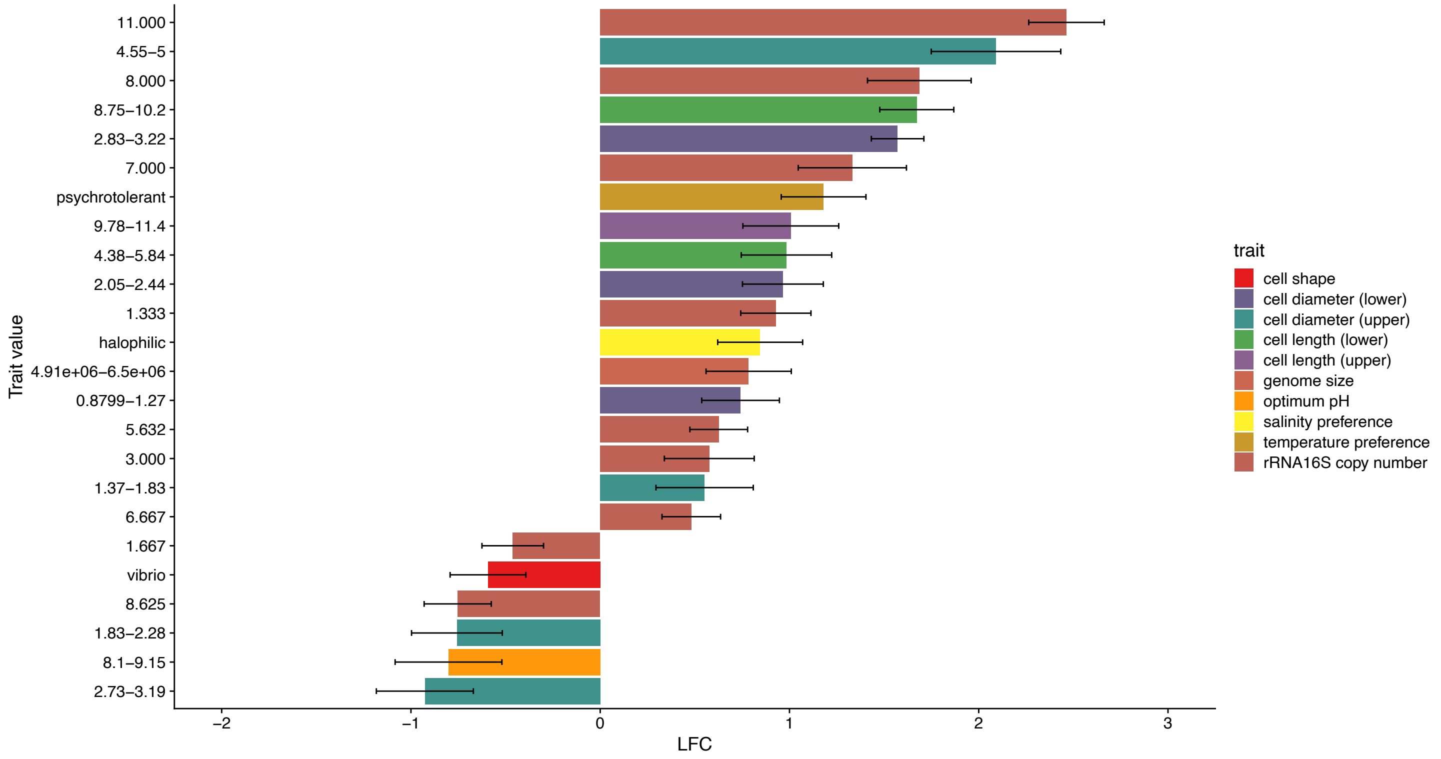

Supplemental Figure 11. *amplicontraits* reveals functional differences among the microbial communities of *C. elegans* (Log Fold Change (LFC) < 0) and *C. inopinata* (LFC > 0) substrates. Details of traits, trait values, and their units can be found in the *amplicontraits* documentation (https://github.com/jdonhauser/ampliconTraits).

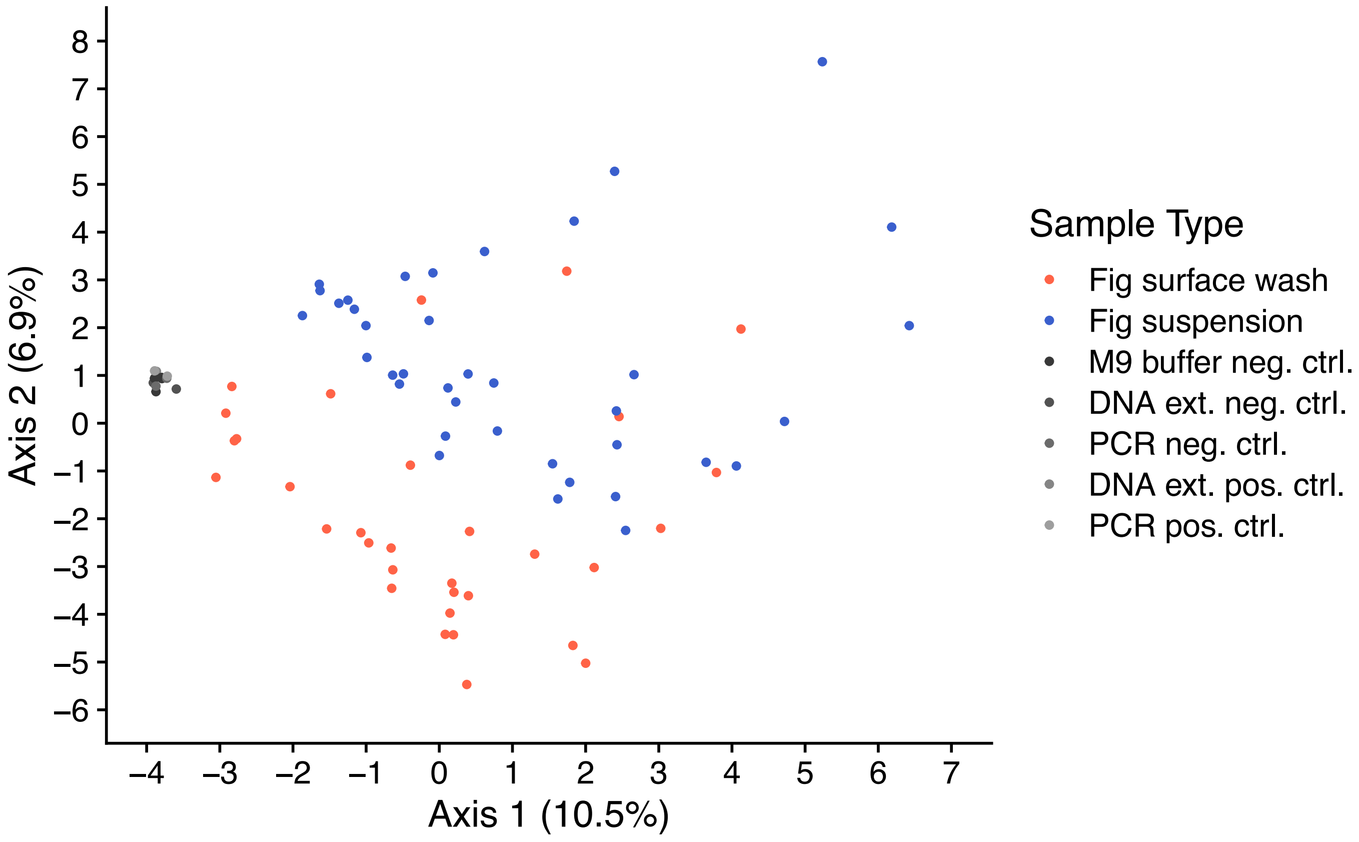

Supplemental Figure 12. Ordination of fig samples including control samples. "neg. ctrl." = negative control; "pos. ctrl." = positive control; "DNA ext." = DNA extraction.

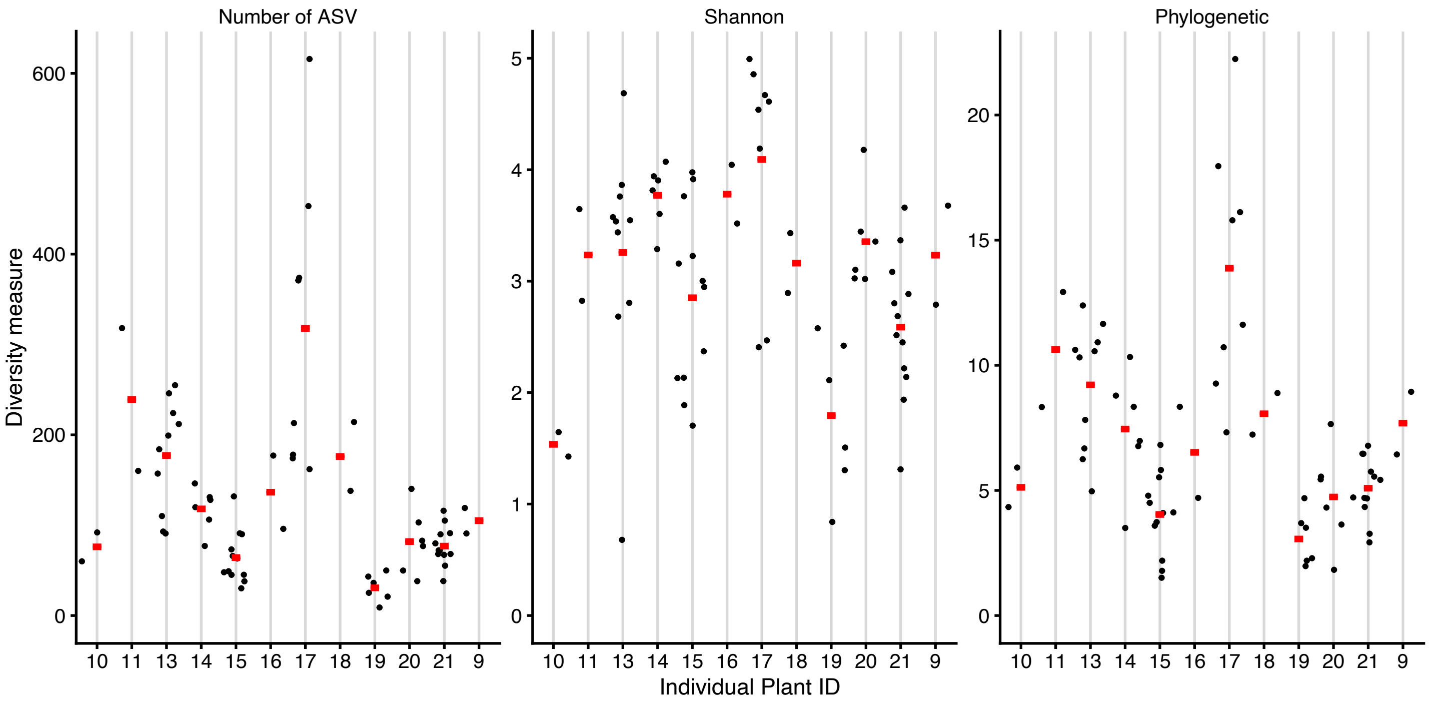

Supplemental Figure 13. Within-sample microbial diversity varies across individual plants.

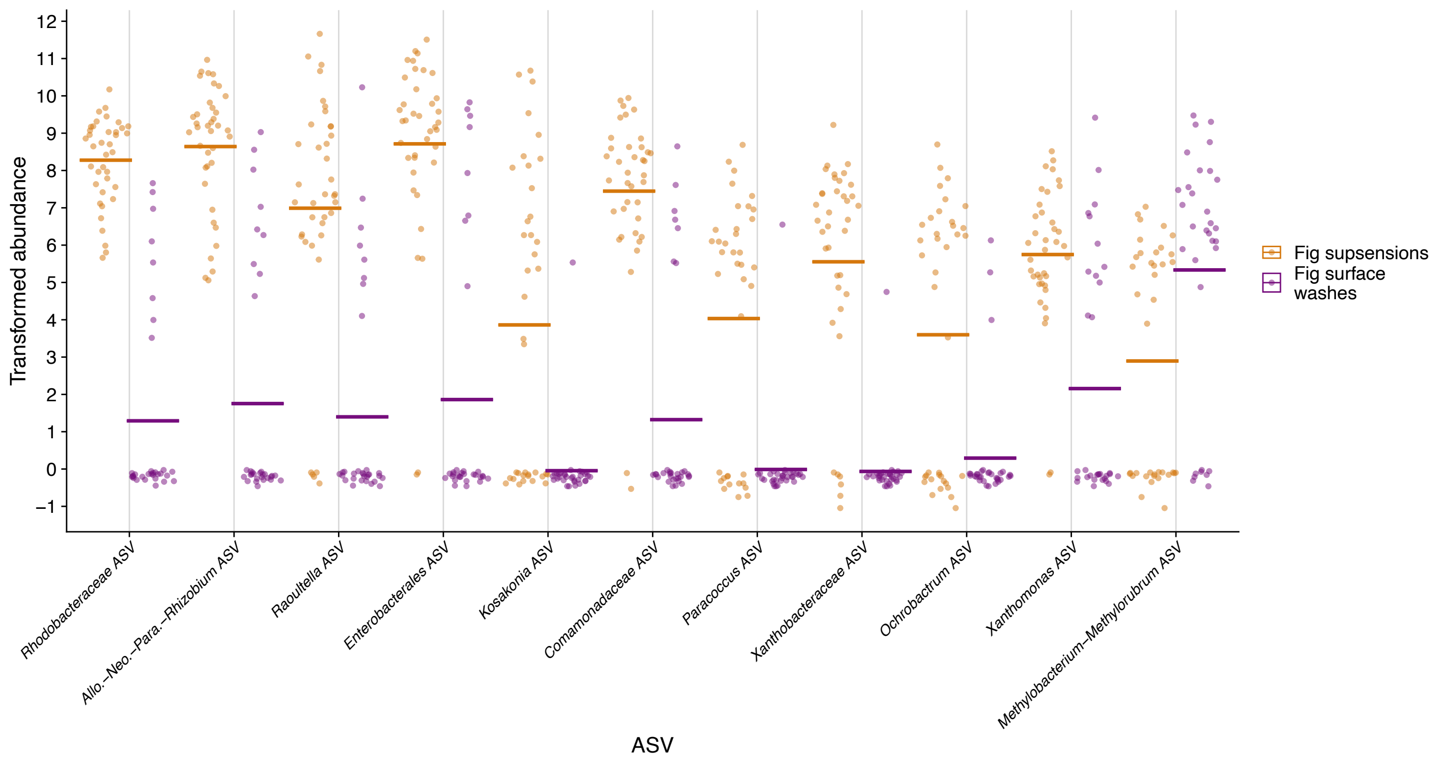

Supplemental Figure 14. ASVs differentially abundant among fig suspensions and fig surface washes. Plotted are centered log-ratio transformed abundances by sample type (fig suspensions or surface washes). The eleven significant ASV are plotted and ordered by effect size. Sina plots are strip charts that take the contours of a violin plot. Horizontal bars represent means.

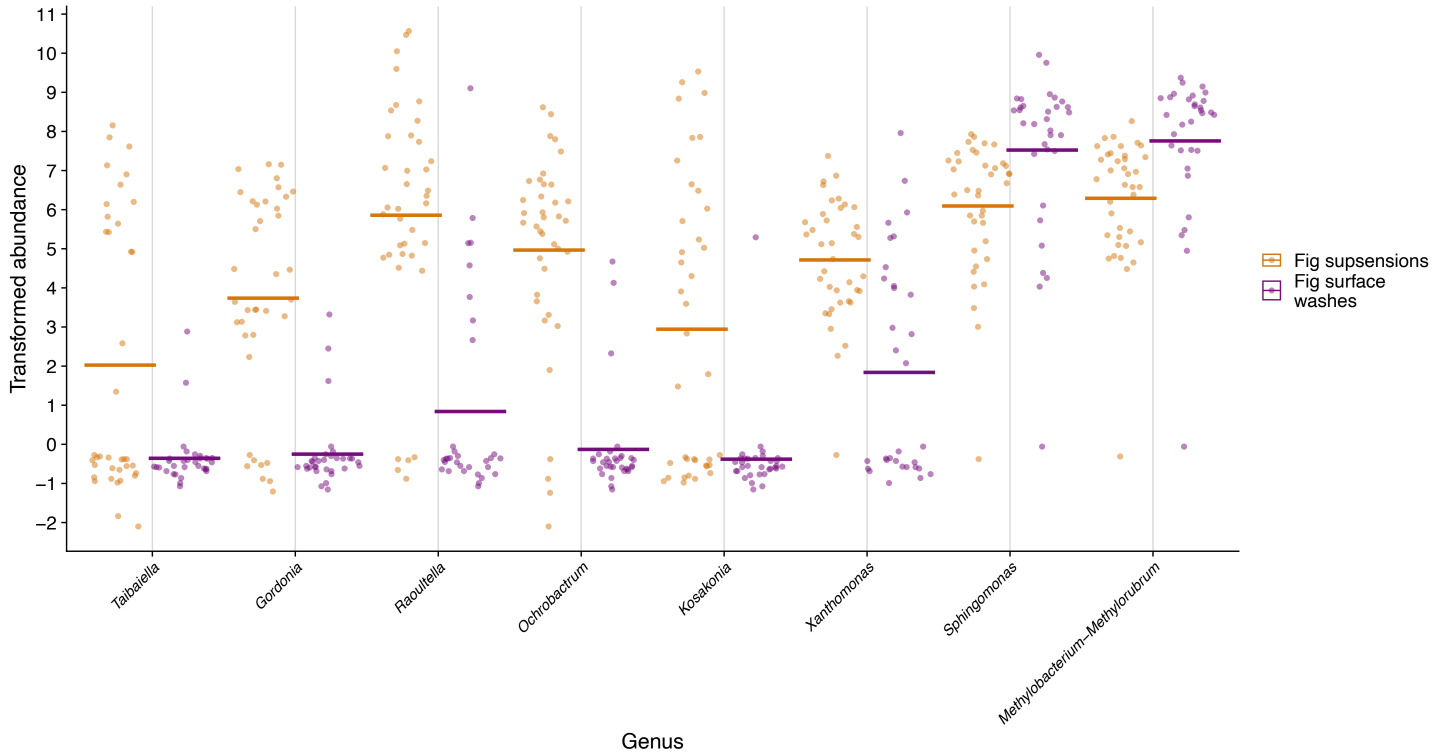

Supplemental Figure 15. Genera differentially abundant among fig suspensions and fig surface washes. Plotted are centered log-ratio transformed abundances by sample type (fig suspensions or surface washes). 8 out of 12 significant genera are plotted and ordered by effect size. Sina plots are strip charts that take the contours of a violin plot. Horizontal bars represent means.

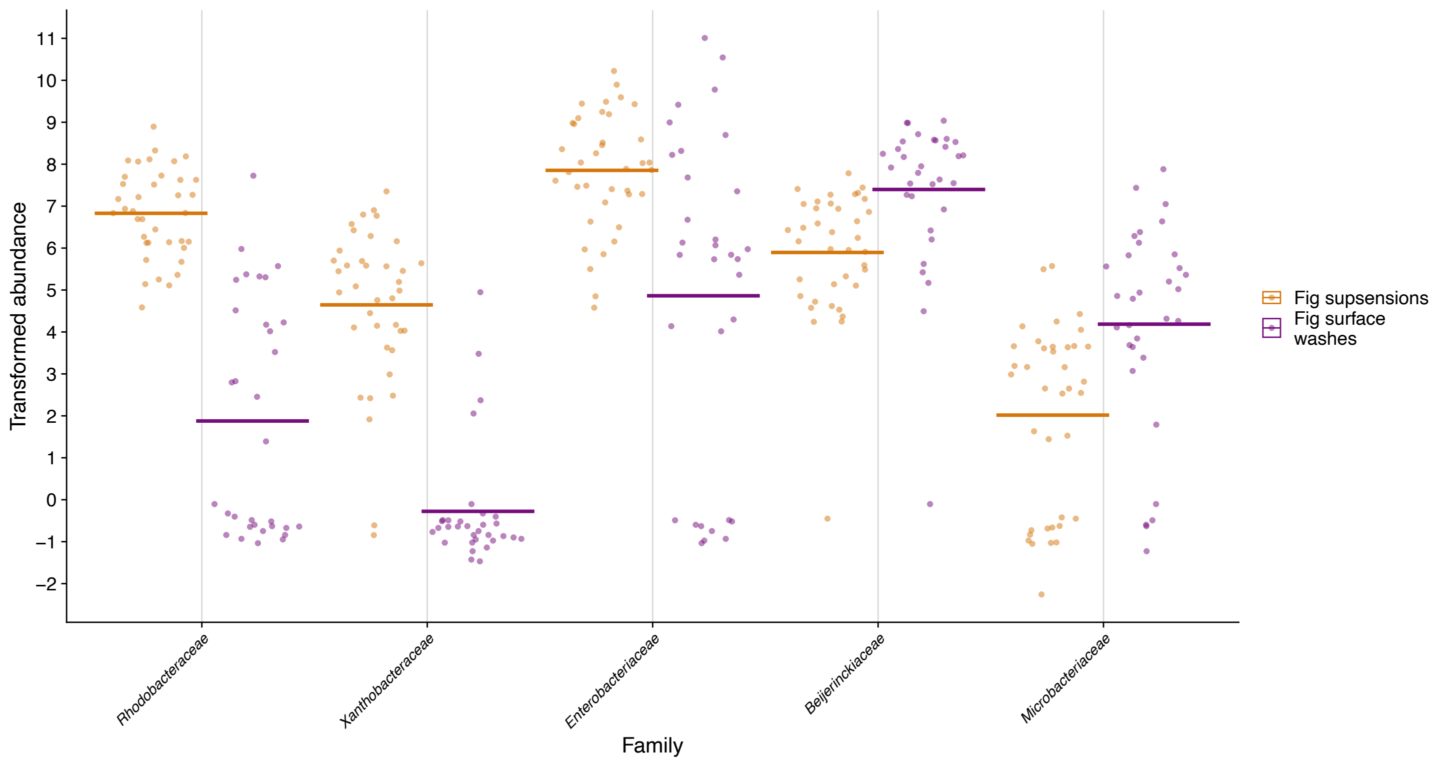

Supplemental Figure 16. Families differentially abundant among fig suspensions and fig surface washes. Plotted are centered log-ratio transformed abundances by sample type (fig suspensions or surface washes). Five significant families (not including one taxonomically unclassified family) are plotted and ordered by effect size. Sina plots are strip charts that take the contours of a violin plot. Horizontal bars represent means.

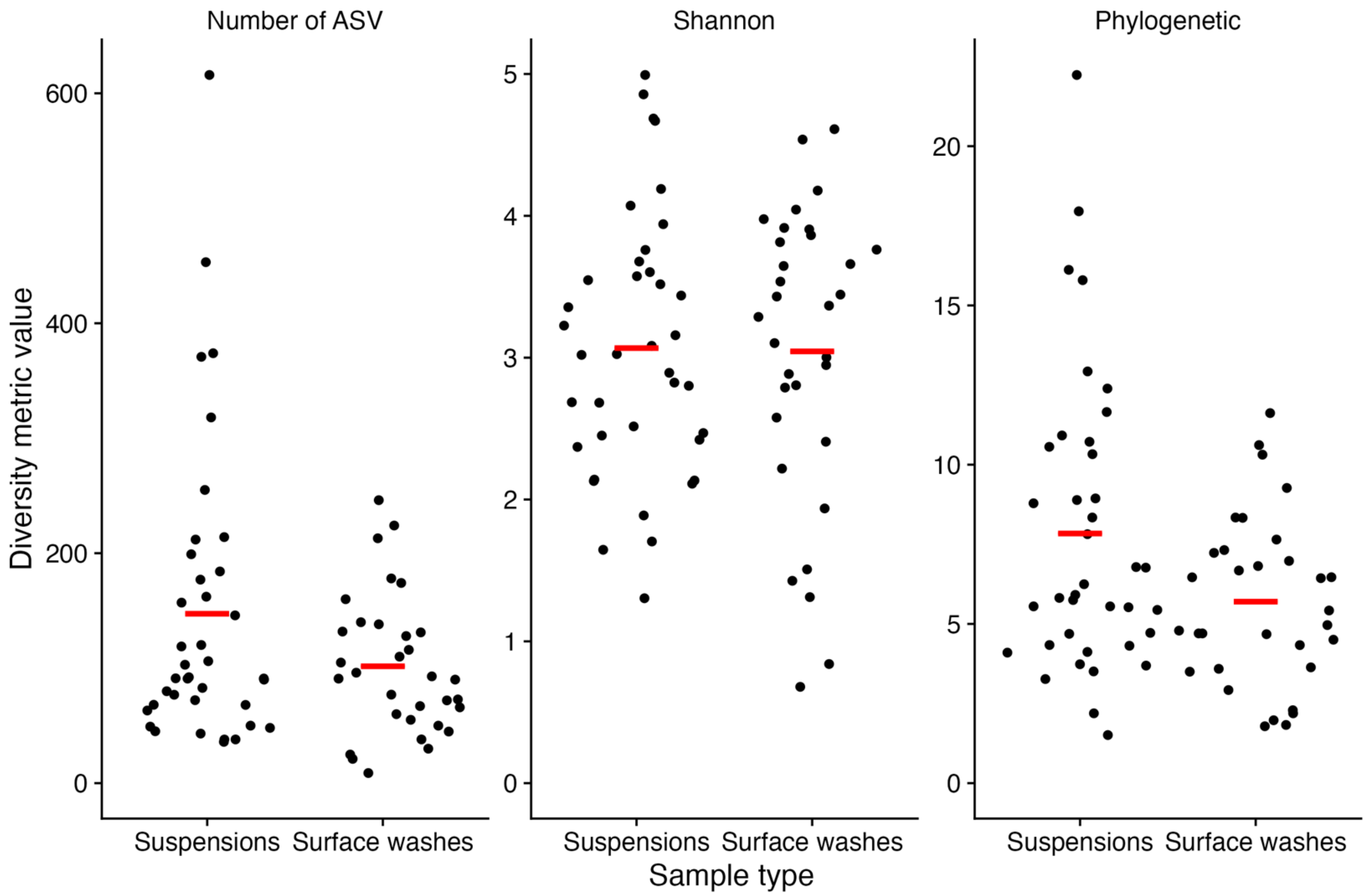

Supplemental Figure 17. Microbial α-diversity is comparable among fig suspensions and fig surface washes. Three diversity metrics (number of ASVs found in each sample; Shannon diversity; and Phylogenetic diversity) are plotted. Sina plots are strip charts with points taking the contours of a violin plot. Horizontal bars represent means.

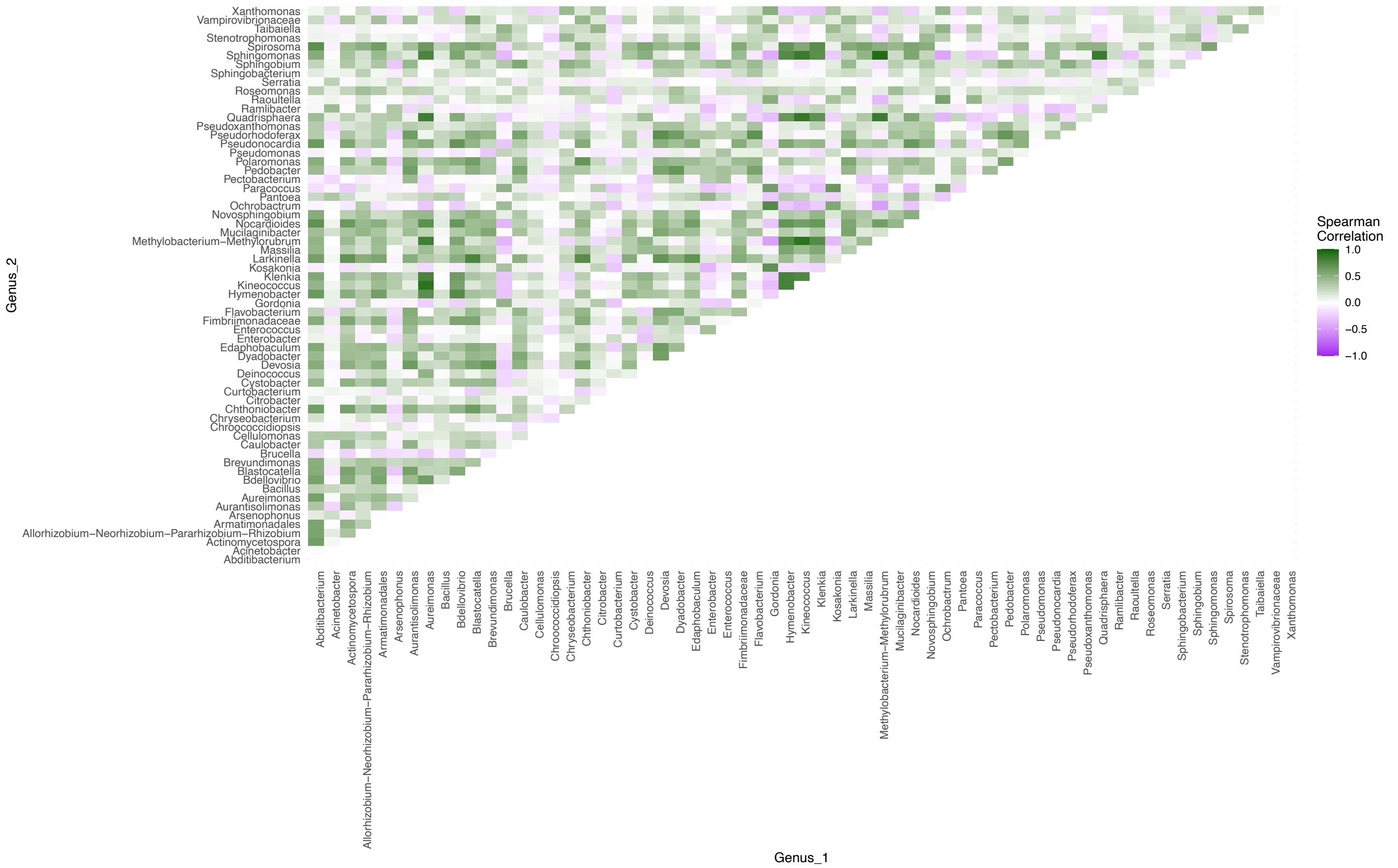

Supplemental Figure 18. Co-occurrence of bacterial genera among fig samples. Green colors represent genera whose abundances are positively correlated among samples; purple colors represent genera whose abundances are negatively correlated among samples.

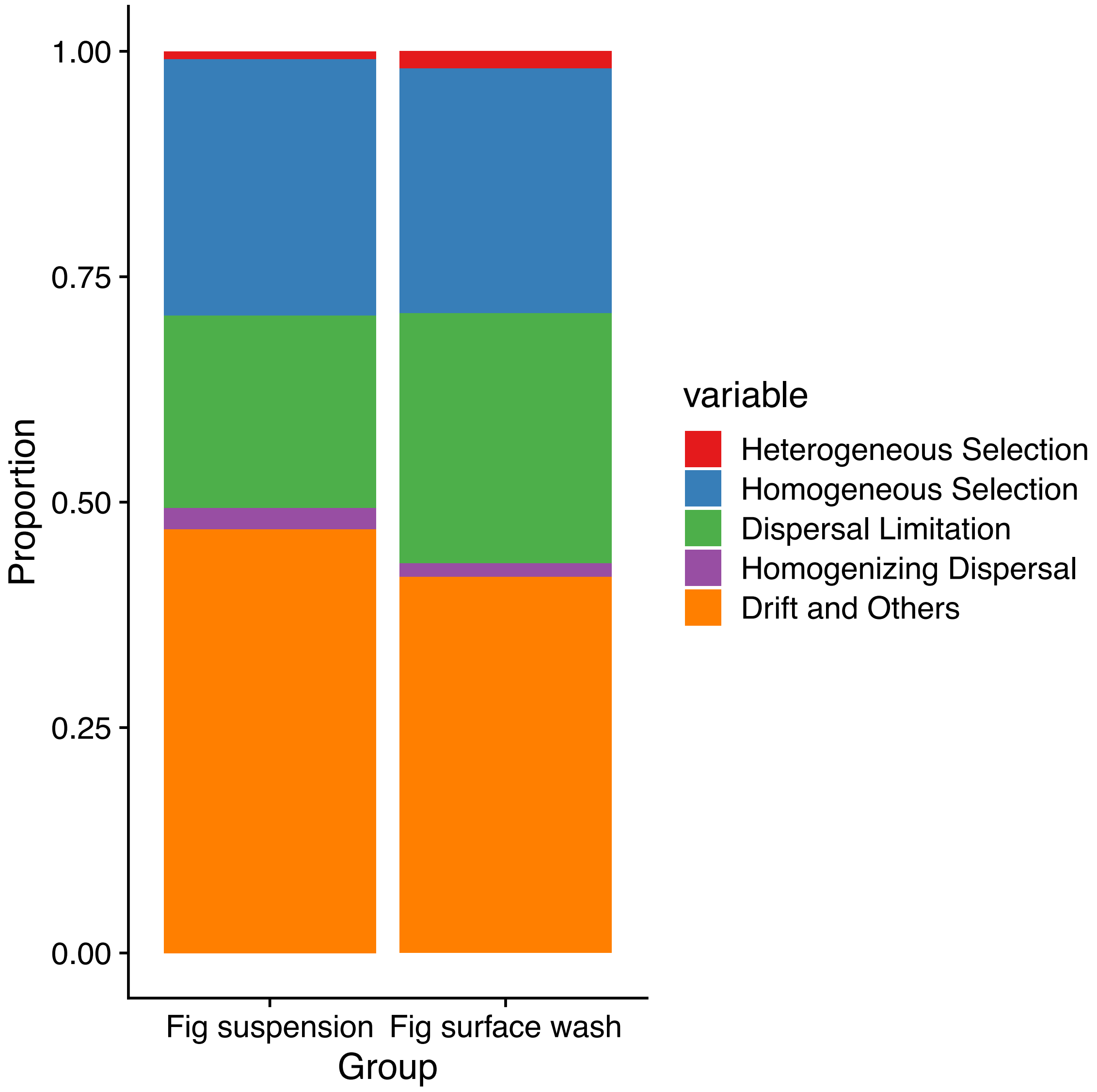

Supplemental Figure 19. iCAMP analysis reveals relative contributions of stochastic and deterministic processes of community assembly of fig microbiomes. Stochastic processes (Drift and others, dispersal limitation, and homogenizing dispersal) contribute more to community assembly than deterministic processes (homogenous and heterogenous selection).

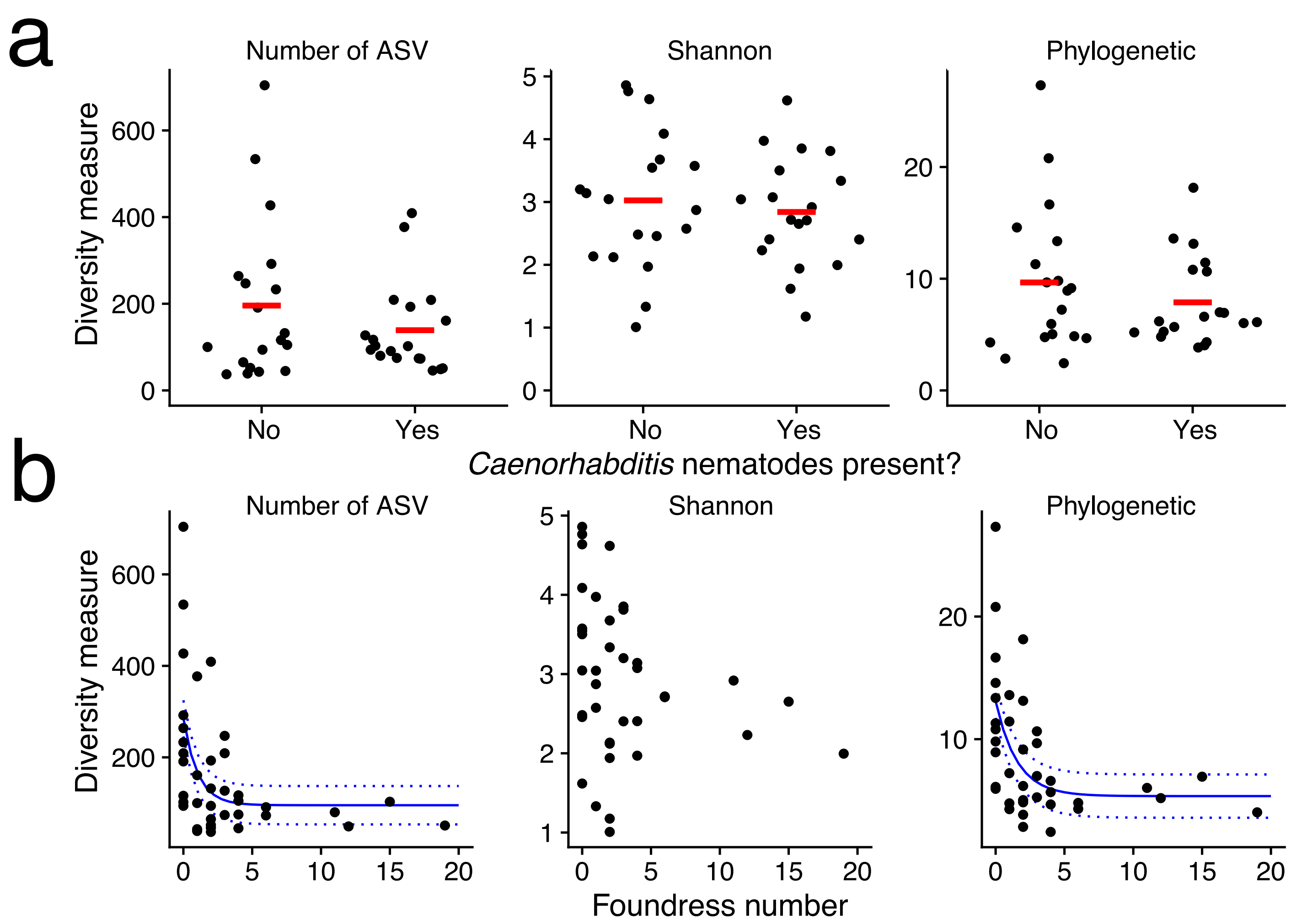

Supplemental Figure 20. Alpha diversity among figs with differing *Caenorhabditis* nematode occupancy (a) and foundress wasp number (b). Asymptotic models were fit with self-starting nonlinear least squares functions within the base R programming language (function *nls*(Diversity metric ~ *SSamp*(foundress number)). Dotted lines represent 95% confidence intervals. The data in the second panel of (b) did not yield a model that converged.

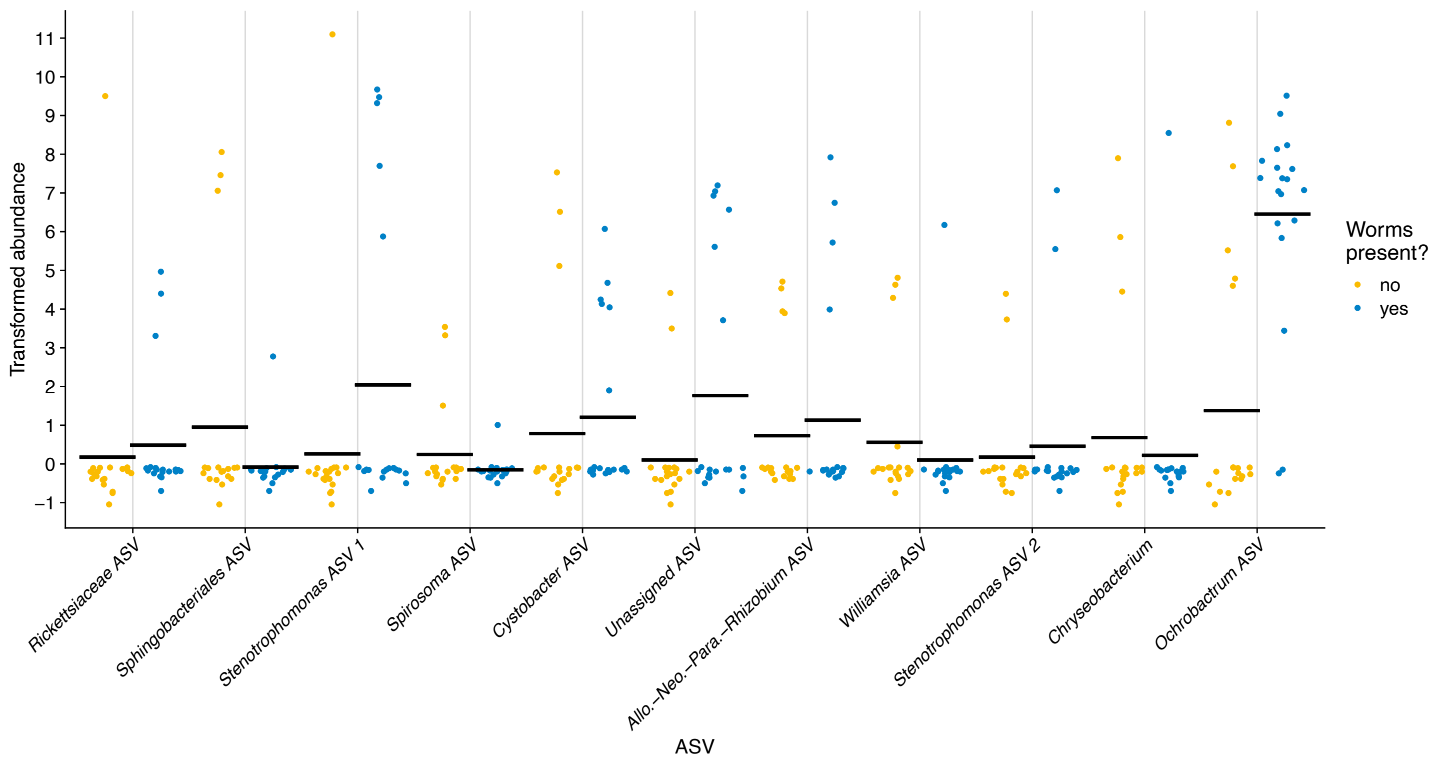

Supplemental Figure 21. ASVs differentially abundant among fig suspensions with and without nematodes. Plotted are centered log-ratio transformed abundances by sample type (fig suspensions or surface washes). The 11 ASV with significant FDR-adjusted Wald test *p*-values are plotted (Only the *Ochobactrum* ASV comparison passes the ANCOM-BC2 sensitivity analysis). Sina plots are strip charts that take the contours of a violin plot. Horizontal bars represent means.

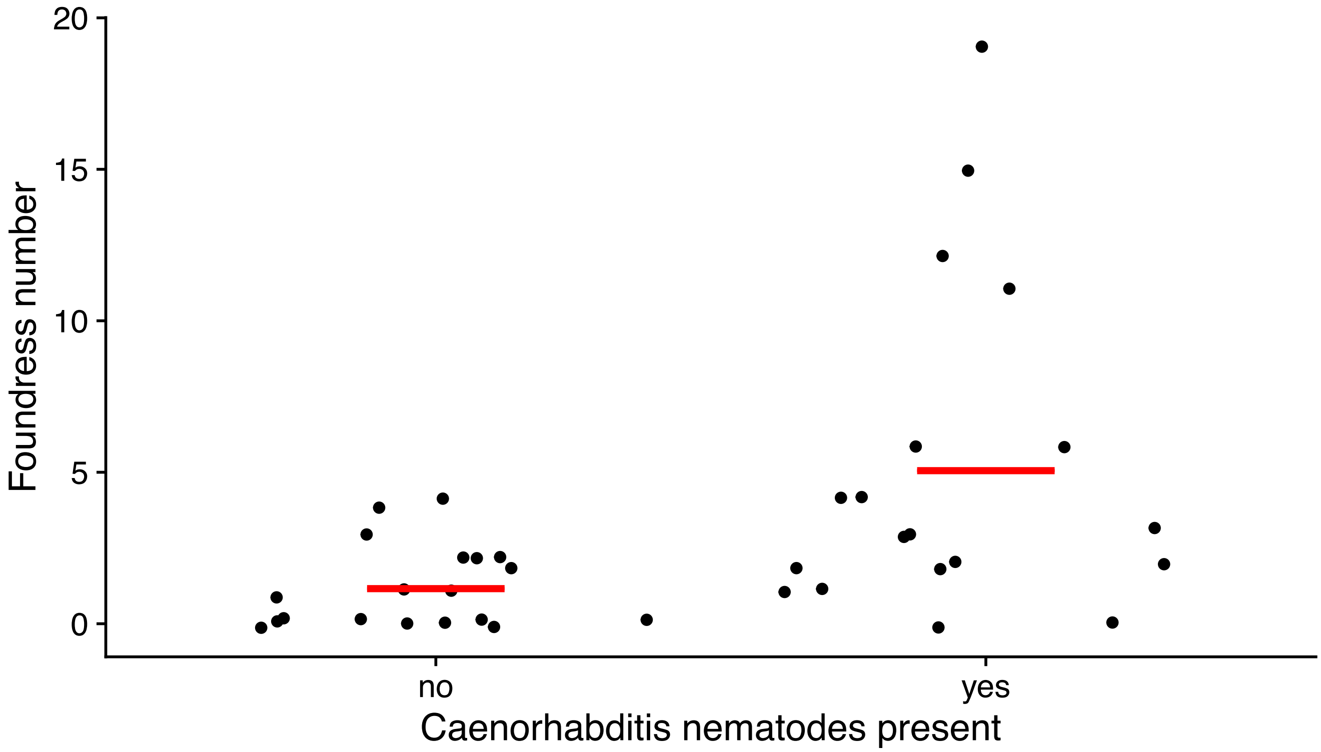

Supplemental Figure 22. Figs harboring nematodes have more foundress wasps.

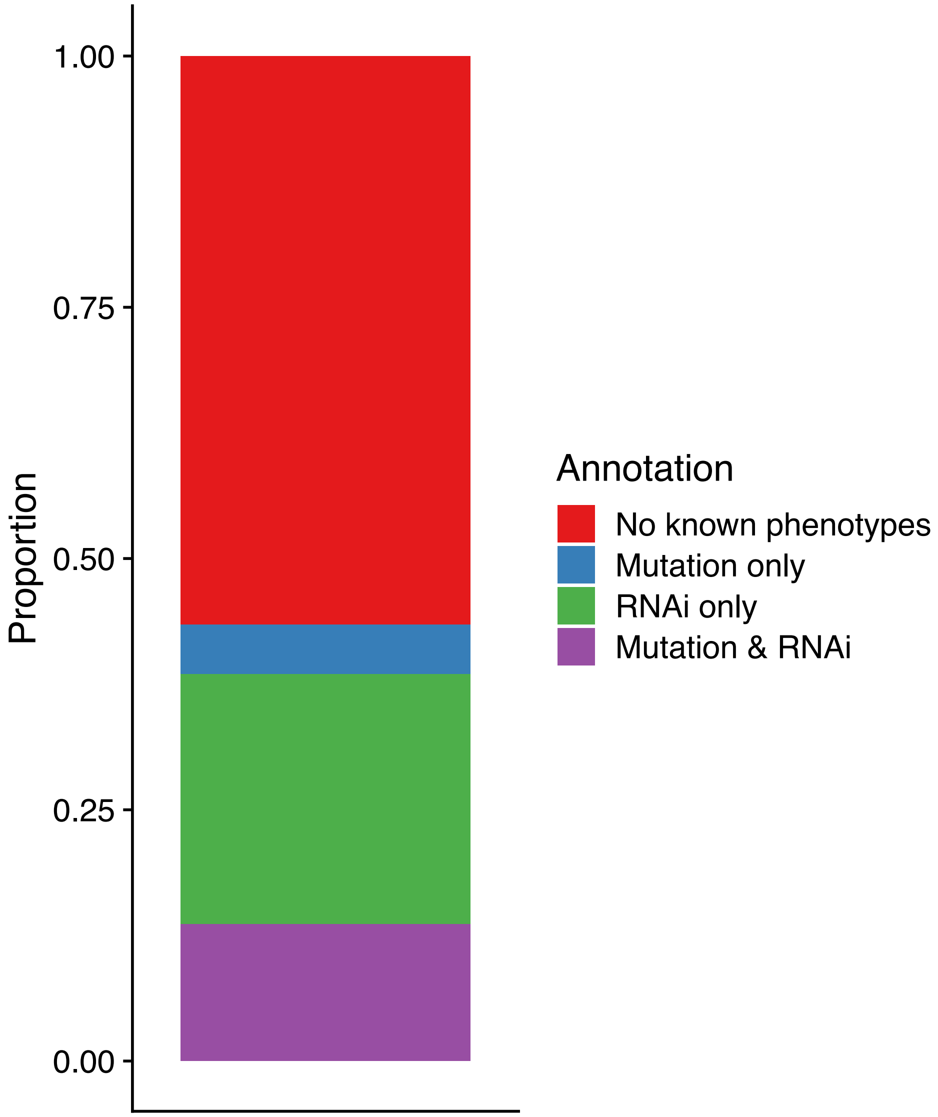

Supplemental Figure 23. More than half of *C. elegans* genes have no reported phenotypic function. Among 19,984 protein-coding genes, 11,309 (57%) have no reported RNAi- or mutation-associated perturbation phenotypes in the WormBase database. Briefly, the WormBase SimpleMine tool (https://wormbase.org/tools/mine/simplemine.cgi) was used to retrieve all *C. elegans* protein-coding gene annotations. Genes with and without RNAi and Allele Phenotype annotations were then counted and plotted. The data and code used to generate this plot have been deposited in Github (https://github.com/gcwoodruff/F_septica_16S_microbial_ecology_2024/revisions_1).

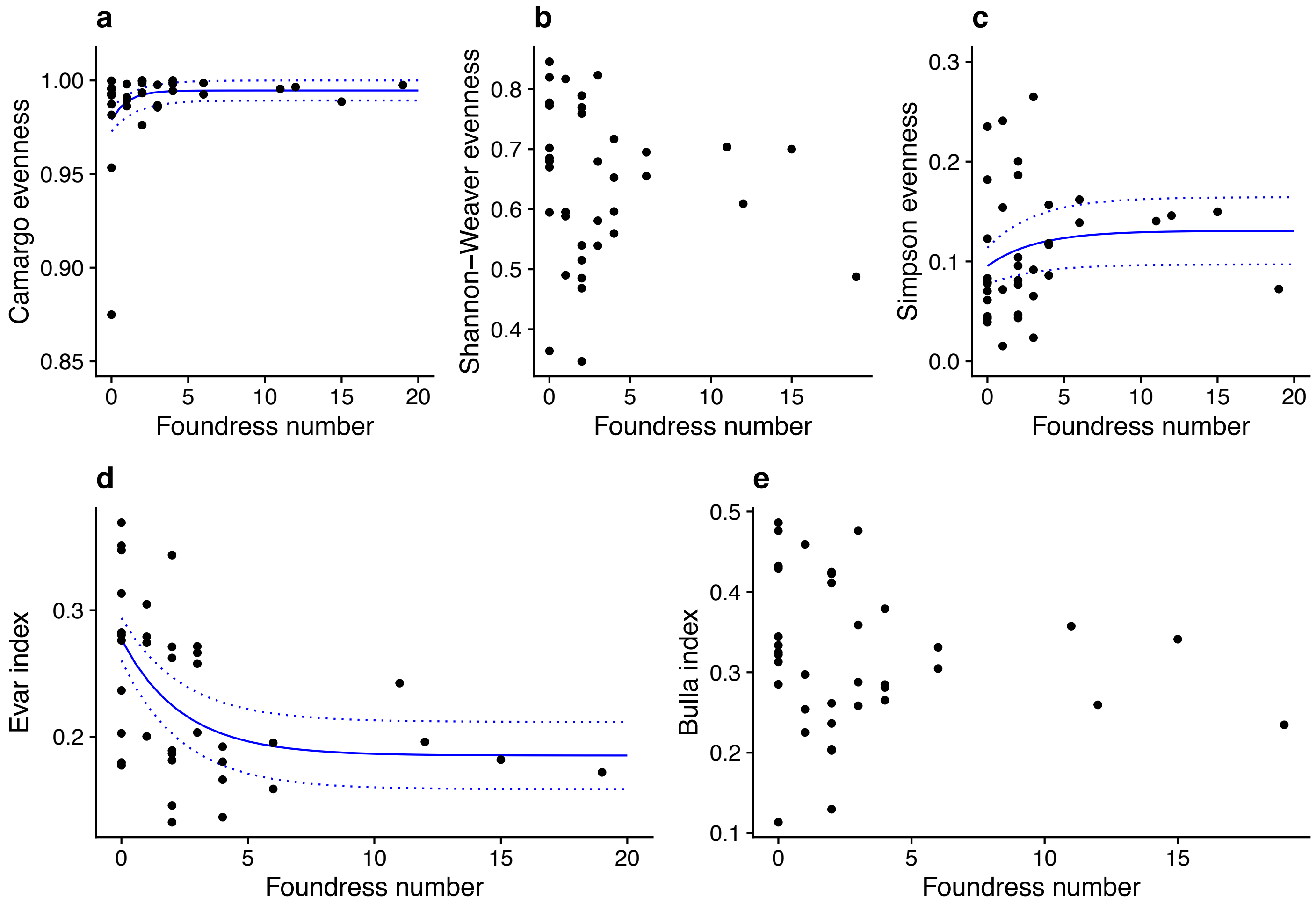

Supplemental Figure 24. The relationship between microbial community evenness and foundress number. (a) "carmago": Camargo's evenness. (b) Shannon-Weaver/Wiener evenness, H/ln(S). (c) Simpson's evenness (inverse Simpson diversity/S). (d) "evar": Smith and Wilson's Evar index. (e) Bulla's index. Asymptotic models were fit with self-starting nonlinear least squares functions within the base R programming language (function *nls*(Diversity metric ~ *SSamp*(foundress number)). Dotted lines represent 95% confidence intervals. Panels without fits did not yield models that converged.

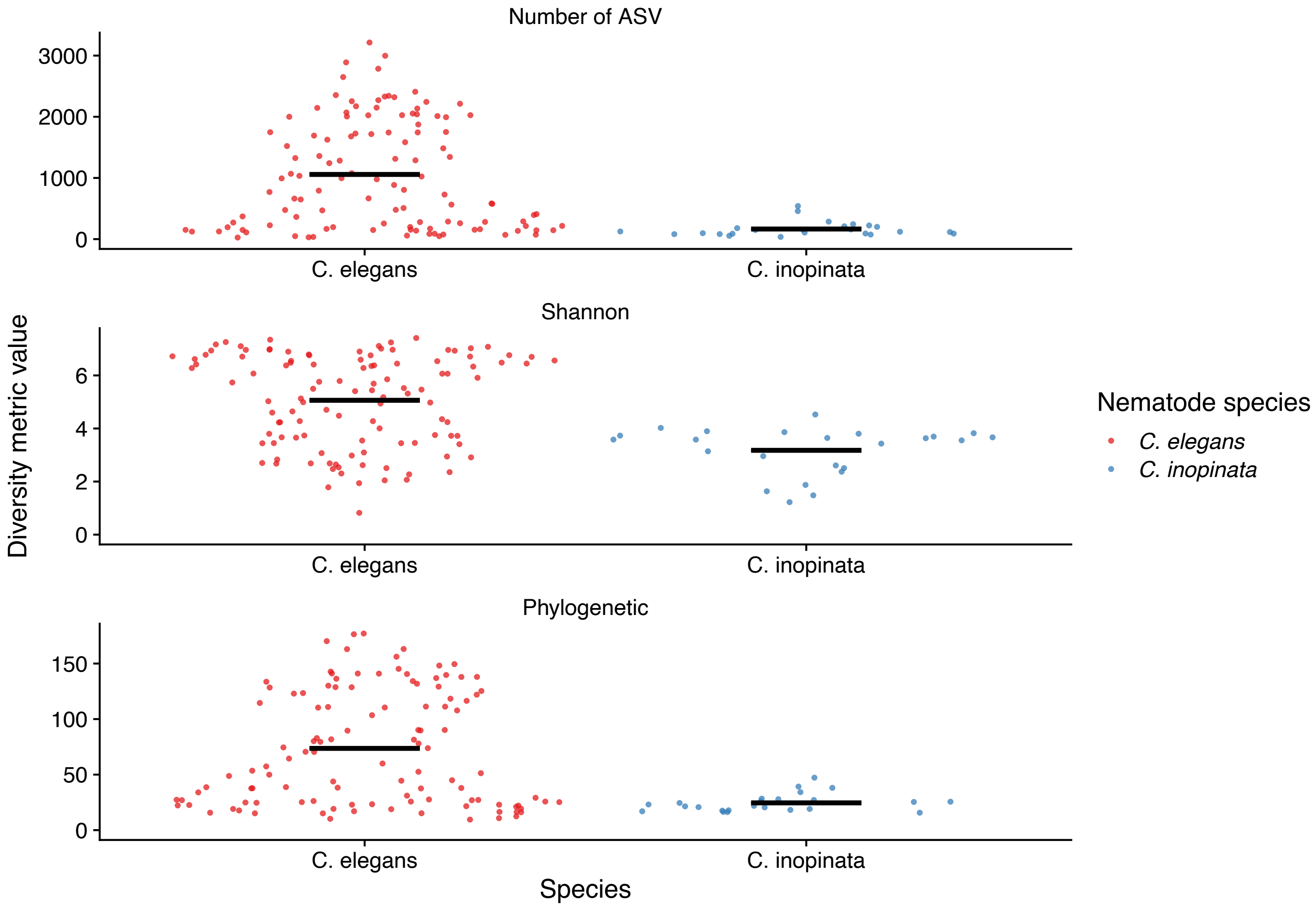

Supplemental Figure 25. α-diversity is higher in *C. elegans* substrates compared to *C. inopinata* substrates. These values are similar to those reported in Supplemental Figure 4, but here, within-plant means have been generated for all *C. inopinata* samples. Wilcoxon rank-sum test $p < 0.001$ for all diversity measures.

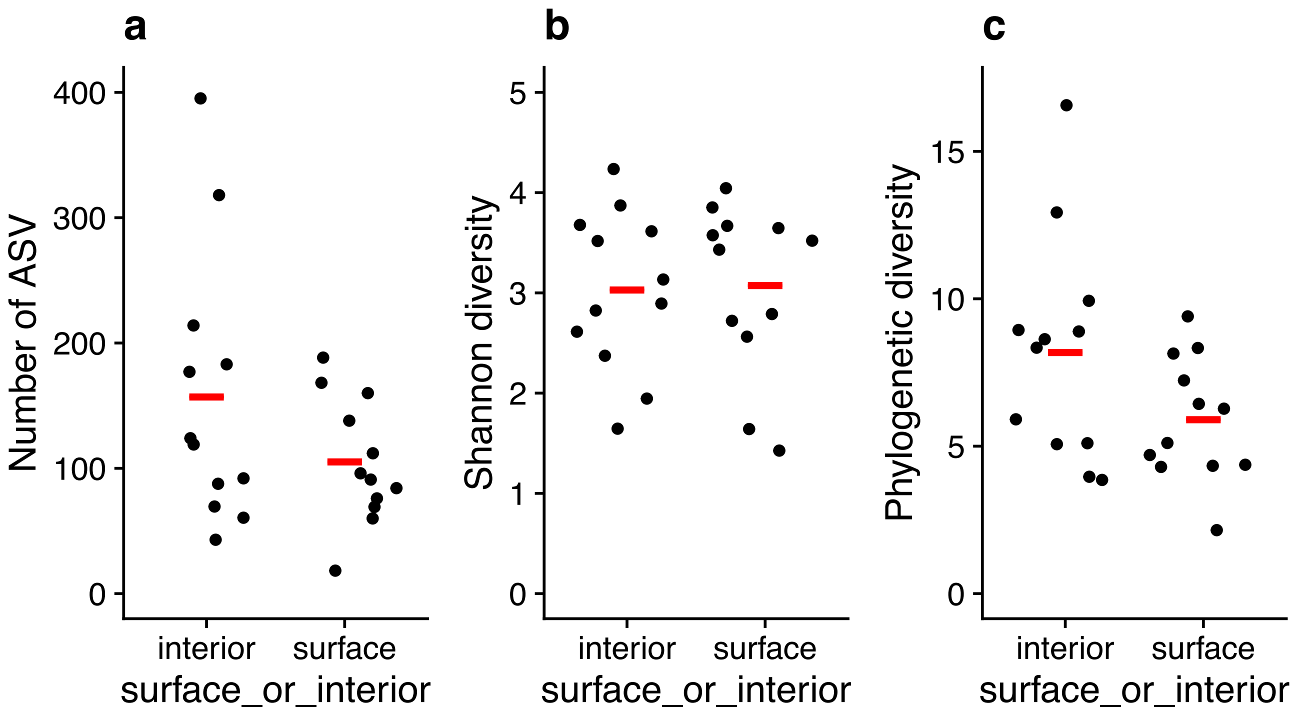

Supplemental Figure 26. Microbial α-diversity is comparable among fig suspensions and fig surface washes. These values are similar to those reported in Supplemental Figure 17, but here, within-plant means have been generated for all samples. "Interior"=Fig suspension; "surface"=Fig surface wash. Wilcoxon rank-sum test $p = 0.29$ (Number of ASV), 0.98 (Shannon diversity), and 0.14 (Phylogenetic diversity).

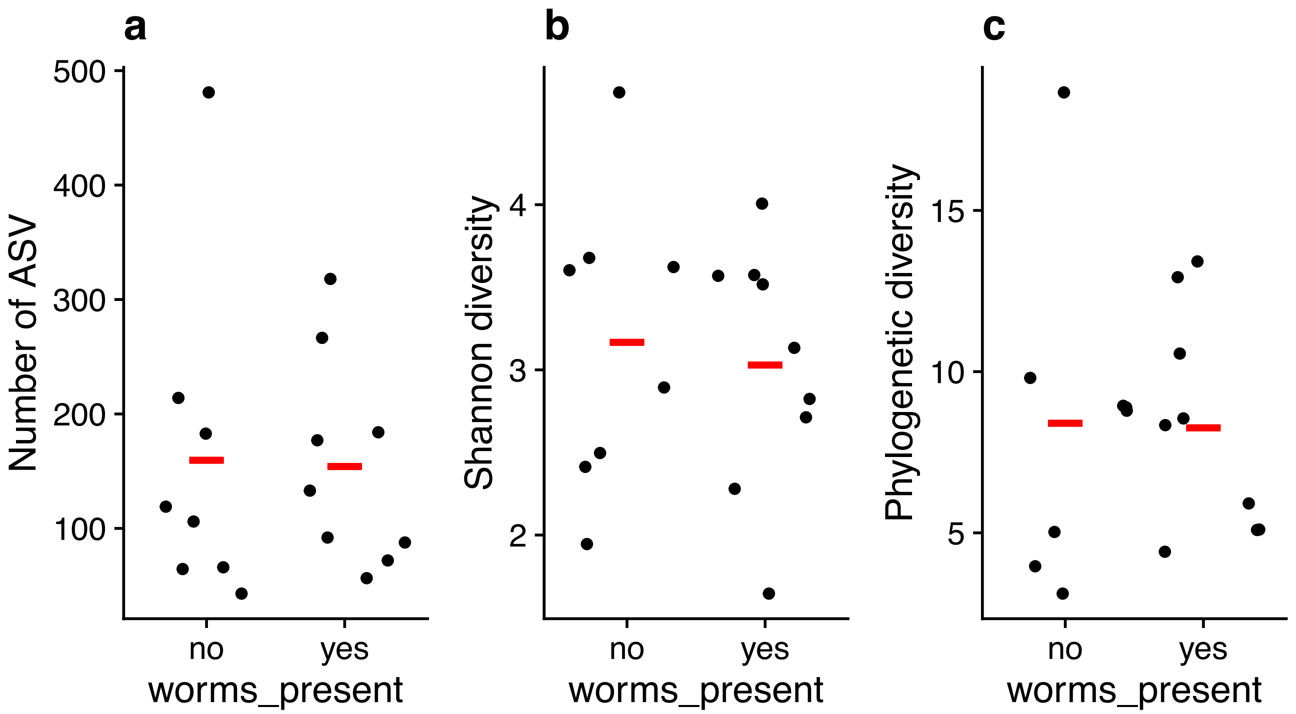

Supplemental Figure 27. Microbial α-diversity is comparable among fig suspensions with and without nematodes. These values are similar to those reported in Supplemental Figure 20a, but here, within-plant means have been generated for all samples. Wilcoxon rank-sum test $p = 0.74$ (Number of ASV), 0.61 (Shannon diversity), and 0.89 (Phylogenetic diversity).
